# Ames test study designs for nitrosamine mutagenicity testing: qualitative and quantitative analysis of key assay parameters

**DOI:** 10.1093/mutage/gead033

**Published:** 2023-12-19

**Authors:** Dean N Thomas, John W Wills, Helen Tracey, Sandy J Baldwin, Mark Burman, Abbie N Williams, Danielle S G Harte, Ruby A Buckley, Anthony M Lynch

**Affiliations:** GSK Research & Development, Genetic Toxicology and Photosafety, Stevenage SG1 2NY, United Kingdom; GSK Research & Development, Genetic Toxicology and Photosafety, Stevenage SG1 2NY, United Kingdom; GSK Research & Development, Genetic Toxicology and Photosafety, Stevenage SG1 2NY, United Kingdom; GSK Research & Development, Genetic Toxicology and Photosafety, Stevenage SG1 2NY, United Kingdom; GSK Research & Development, Genetic Toxicology and Photosafety, Stevenage SG1 2NY, United Kingdom; GSK Research & Development, Genetic Toxicology and Photosafety, Stevenage SG1 2NY, United Kingdom; GSK Research & Development, Genetic Toxicology and Photosafety, Stevenage SG1 2NY, United Kingdom; GSK Research & Development, Genetic Toxicology and Photosafety, Stevenage SG1 2NY, United Kingdom; GSK Research & Development, Genetic Toxicology and Photosafety, Stevenage SG1 2NY, United Kingdom; School of Medicine, Swansea University, Singleton Park, Swansea SA2 8PP, United Kingdom

**Keywords:** NDMA, NDEA, NMEA, bacterial reverse mutation test, bench mark dose, regulatory testing

## Abstract

The robust control of genotoxic *N*-nitrosamine (NA) impurities is an important safety consideration for the pharmaceutical industry, especially considering recent drug product withdrawals. NAs belong to the ‘cohort of concern’ list of genotoxic impurities (ICH M7) because of the mutagenic and carcinogenic potency of this chemical class. In addition, regulatory concerns exist regarding the capacity of the Ames test to predict the carcinogenic potential of NAs because of historically discordant results. The reasons postulated to explain these discordant data generally point to aspects of Ames test study design. These include vehicle solvent choice, liver S9 species, bacterial strain, compound concentration, and use of pre-incubation versus plate incorporation methods. Many of these concerns have their roots in historical data generated prior to the harmonization of Ames test guidelines. Therefore, we investigated various Ames test assay parameters and used qualitative analysis and quantitative benchmark dose modelling to identify which combinations provided the most sensitive conditions in terms of mutagenic potency. Two alkyl-nitrosamines, *N*-nitrosodimethylamine (NDMA) and *N*-nitrosodiethylamine (NDEA) were studied. NDMA and NDEA mutagenicity was readily detected in the Ames test and key assay parameters were identified that contributed to assay sensitivity rankings. The pre-incubation method (30-min incubation), appropriate vehicle (water or methanol), and hamster-induced liver S9, alongside *Salmonella typhimurium* strains TA100 and TA1535 and *Escherichia coli* strain WP2uvrA(pKM101) provide the most sensitive combination of assay parameters in terms of NDMA and NDEA mutagenic potency in the Ames test. Using these parameters and further quantitative benchmark dose modelling, we show that *N*-nitrosomethylethylamine (NMEA) is positive in Ames test and therefore should no longer be considered a historically discordant NA. The results presented herein define a sensitive Ames test design that can be deployed for the assessment of NAs to support robust impurity qualifications.

## Introduction

The most frequent assay used to assess chemically induced gene mutation is the bacterial reverse mutation test, developed by Bruce Ames in the early 1970s [1,2] and therefore commonly known as the Ames test. This is a short-term assay, which employs various *Salmonella typhimurium* and *Escherichia coli* bacterial strains, that are specifically designed to detect an extensive range of deoxyribonucleic acid (DNA) reactive chemicals, resulting in fixed gene mutation. The Ames test is used ubiquitously, worldwide, as a hazard screen to elucidate mutagenic capacity of new chemical entities, active pharmaceutical ingredients, [3] or potential genotoxic impurities in pharmaceutical products [4] and therefore, the potential for genotoxic carcinogenicity.

The Ames test was originally validated using 300 chemicals, many of which were known rodent carcinogens [5–8]. Subsequent reviews of assay performance [9] and further validation efforts were published, that is, Imperial Chemical Industries [10]; the National Cancer Center Research Institute in Tokyo [11]; the International Agency for Research on Cancer [12]. These studies addressed assay sensitivity and established a good correlation between mutagenicity in the Ames test and carcinogenicity in the animal (rodent) cancer bioassay. A mutagenic response in the Ames test is predictive of rodent carcinogenicity with high concordance, ranging from ~90% [6] to 77% [13], depending on ‘chemical space’, that is the ensemble of molecules tested. Based on these and other studies, recommendations were developed for the conduct of the Ames test [14]. These informed later regulatory guidelines, including those from the Organisation for Economic Co-operation and Development (OECD) and the International Council on Harmonisation of Technical Requirements for Pharmaceuticals for Human Use (ICH).

The Ames test relies on a simple yet elegant premise. Several bacterial strains containing defined mutations in their histidine (*S. typhimurium)* or tryptophan (*E. coli*) operons are exposed to a test agent, to determine if the compound can induce a further mutation that will directly reverse or suppress the original, strain-specific, and mutations. Consequently, the bacterial strains, which are auxotrophic and unable to grow in the absence of certain essential amino acids (histidine for *S. typhimurium* or tryptophan for *E.coli*), are restored to prototrophy by a ‘reverse mutation’ and mutant bacteria are able to grow and form colonies in the absence of histidine or tryptophan. These so-called ‘revertant colonies’ have ‘returned’ to their wild-type phenotype and counting the number of revertant colonies induced by a test agent provides investigators with an expedient endpoint to measure mutagenic capacity. This is because millions of bacteria are exposed per treatment concentration and therefore, even rare events, such as mutation can be determined with a high degree of sensitivity [9].

Several genetic traits have been intentionally selected in the bacterial strains used in the Ames test to increase their sensitivity to fix mutations. Many carcinogens and their subsequent metabolites are large molecules and would be unable to penetrate the cell walls of wild-type bacteria. This is because lipopolysaccharide (LPS) acts as a barrier to bulky hydrophobic molecules. To combat this, the *Salmonella* strains used in the Ames test contain a mutation in the *rfa* operon resulting in the ‘deep rough’ phenotype [15] that is associated with LPS deficiency leading to incomplete formation of the smooth outer membrane and surface capsule coating. The ‘deep rough’ phenotype represents the maximum permissible stripping of LPS from the bacterial wall without incurring lethality and enables the uptake of large and hydrophobic molecules into the bacterial cell [16]. Wild-type bacteria also possess several DNA repair pathways. The Ames test bacterial strains carry deletions of the *uvrB* gene [17] that eliminate error-free excision repair and result in more DNA lesions being repaired by an error-prone pathway. This leads to increased mutation and significantly increases the sensitivity of the *Salmonella* strains to chemically induced mutagenesis. Similarly, sensitivity to chemical and UV-induced mutagenesis is enhanced in strains carrying the plasmid pKM101. This plasmid contains the *UmuD* and *UmuC* genes, which are induced as part of the SOS response to DNA damage, and result in error-prone repair [18]. Lastly, post-mitochondrial S9 fractions from cytochrome P450-induced rodent liver preparations are used in the Ames test to provide an exogenous metabolic system. The liver S9-mix preparations contain increased phase-1 enzymic activation capacity but limited phase-2 enzyme capacity. As such, the metabolic system used in the Ames test is biased towards the activation of pro-mutagens rather than detoxification, which also enhances the sensitivity of the test for hazard identification.

In recent years there have been regulatory concerns regarding the capacity of the Ames test to predict the carcinogenic potential of a sizable proportion of chemicals that belong to the *N*-nitrosamine (NA) class of chemicals. This is because historically, several NA chemical structures have been deemed discordant in terms of Ames test predictivity of rodent carcinogenicity, that is, they are Ames test negative but rodent cancer bioassay positive. These concerns were first identified by Rao *et al*. [19]. In their work, various aliphatic NAs were assessed for bacterial mutagenicity with 9 out of 18 known rodent carcinogens negative in both plate incorporation and pre-incubation versions of the assay (when tested up to 2 mg/plate). Later, Andrews and Lijinsky [20] evaluated 45 NAs, again reporting a poor correlation between mutagenic potency and carcinogenic potency, with the most discrepancy among asymmetric NAs. For a contemporaneous review of the mutagenic and carcinogenic potencies of NAs, as understood at that time, the reader is referred to a series of commentaries in ‘Topics in Chemical Mutagenesis’ Vol. 1 Genotoxicology of *N*-nitroso compounds [21]. This volume lists various NA exemplars considered discordant for Ames test and rodent cancer bioassay predictivity.

Aspects of Ames test study design have, in part, been postulated as reasons to explain the discordant results with NAs. These include vehicle choice, liver S9 species selection, and other Ames test assay parameters (e.g. bacterial strain, compound concentration, and the use of the pre-incubation method versus plate incorporation). However, many of these concerns also have their roots in the historic mutagenicity data generated for NAs prior to the development (and later refinement) of Ames test guidelines, that is, bacterial reverse mutation test [22]. In these earlier studies, pivotal Ames test assay parameters regarding compound concentration and exposure (plate incorporation vs. pre-incubation), bacterial strain selection, vehicle selection, numbers of replicates, liver S9 species, and S9 concentration/incubation time were not universally agreed or consistently used. The resulting historical perspectives, therefore, rely on a compilation of data from variable assay designs, which could ultimately impact perceptions regarding assay sensitivity, data quality, and study design [14,23–26].

To address some of these concerns about the sensitivity of the Ames test to predict the carcinogenic potential of NAs, Trejo-Martin *et al*. [27] investigated the performance of historical Ames test studies for this chemical class that were deemed OECD TG-471 compliant. Data were extracted from curated proprietary databases containing a range of NA chemical structures (Vitic [*n* = 131] and Leadscope [*n* = 70], respectively). The analysis showed high sensitivity (93%–97%) for the Ames test to identify NAs that were positive in the rodent bioassay with reasonable specificity (55%–86%). The analysis showed the plate incorporation method had similar sensitivity to the preincubation method (i.e. 84%–89% vs. 82-%89%) and there were no significant differences when rat or hamster-induced liver S9 was used for metabolic activation (i.e. 80%–93% vs. 77%–96%). The authors noted the sensitivity of the Ames test remained high, overall, when DMSO was used as a vehicle. Therefore, they concluded that an OECD 471 compliant Ames test was highly sensitive for detecting the mutagenic potential of NAs, although pre-incubation and alternative vehicles were recommended for short-chain aliphatic NAs because of the reported inhibition of mutagenicity when DMSO was used. However, Tennant *et al*. [28] recently showed that hamster S9 generally produces positive results at lower concentrations than rat S9 and the latter is associated with more variable responses, sometimes leading to negative or conflicting calls at lower concentrations.

In the current study, we have extended this work by evaluating the impact of several key experimental assay parameters considered to affect the sensitivity of the Ames test for detecting the potential mutagenicity of NAs. Using OECD TG-471 compliant protocols, two model compounds, *N*-nitrosodimethylamine (NDMA) and *N*-nitrosodiethylamine (NDEA) were chosen based on carcinogenic potency. Plate incorporation and pre-incubation Ames test methods were investigated to assess whether test article exposure in the test system (before the addition of agar) affects assay sensitivity. In addition, the effect of a range of vehicles was investigated (water, DMSO, methanol, acetonitrile, NMP, DMF, acetone, and DHF), as there have been concerns that organic solvent vehicles can inhibit cytochrome P450 enzyme activity and reduce the sensitivity of the Ames test [29,30]. Finally, since the metabolic activation of NAs can be species-dependent [25,31,32] we investigated the use of rat or hamster-induced liver S9 to compare their effects on mutagenic potency, the mechanism of N-alkyl nitrosamine activation involves CYP2E1 metabolism, and this has been extensively described elsewhere [33]. The results of these studies are presented along with a combination of advanced qualitative and quantitative modelling approaches to evaluate the data and identify those Ames test assay parameters that contributed most to overall mutagenic potency.

An Ames test with the most sensitive assay parameters was subsequently used to evaluate *N*-nitrosomethylethylamine (NMEA), a historically discordant NA, that is, Ames test negative [19] and rodent cancer bioassay positive [34]. The results of this study are also presented, along with additional qualitative and quantitative modelling of the NMEA-data. The implications of this work are discussed in terms of the historical context of the performance of the Ames test with nitrosamines. In addition, by using qualitative and quantitative modelling of those assay parameters most associated with mutagenic potency we define a robust study design for the future assessment of NA mutagenicity. This enhanced Ames test design will be used to evaluate a series of historically discordant NA, and the results of this work will be the subject of a follow-on publication.

## Materials and methods

### Compounds and solvent vehicles

*N*-Nitrosodimethylamine (NDMA, CAS # 62-75-9), *N*-nitrosodiethylamine (NDEA, CAS # 55-18-5), and *N*-nitrosomethylethylamine (NMEA, CAS# 10595-95-6) were purchased from Enamine, Ukraine. Ultrapure water, dimethylsulfoxide (DMSO), methanol, acetonitrile, *N*-methyl-2-pyrrolidone (NMP), dimethylformamide (DMF), acetone, and dihydrofuran (DHF) were used as vehicles (Sigma–Aldrich, Haver Hill, UK). Table 1 lists the test chemicals, their purity, and sources/batches used, along with basic elements of study composition per test article.

**Table 1.** A summary of compound details and testing conditions.

Compound	Batch (purity, source)	Method (plate or pre)	Vehicle	Strains	S9 (species and inducer)	S9 %, source and batch number	Preincub time	Concentrations tested
NDMA (CAS 62-75-9)	2020-0369507 and 2020-0377826 (95.0%, enamine)	Plate incorporation and preincubation	DMSO, methanol, water, NMP, acetone, acetonitrile, DHF and DMF	*S. typhimurium* TA100, TA98, TA1535, TA1537, *E. coli* WP2 *uvrA* pKM101	Phenobarb/ß-naphthoflavone induced Sprague–Dawley rat liver S9-mix or Aroclor-induced golden Syrian hamster liver S9-mix (final volume 500 L S9 mix/plate)	10%, Moltox 4050	30 min	50–5000 µg/plate
NDEA (CAS 55-18-5)	2019-0305974 (95.0%, enamine)	Plate incorporation and preincubation	DMSO, methanol, water, NMP, acetone, acetonitrile, DHF and DMF	*S. typhimurium* TA100, TA98, TA1535, and TA1537, *E. coli* WP2 *uvrA* (pKM101)	Phenobarb/ß-naphthoflavone-induced Sprague-Dawley rat liver S9-mix or Aroclor-induced golden Syrian hamster liver S9-mix (final volume 500 l S9 mix/plate)	10%, Moltox 4050	30 minutes	50–5000 µg/plate
NMEA (CAS 10595-95-6)	2019-0321669 (95.0%, enamine)	Plate incorporation and preincubation	DMSO, methanol and water	*S. typhimurium* TA100, TA98, TA1535, TA1537, *E. coli* WP2 *uvrA* pKM101	Phenobarb/ß-naphthoflavone induced Sprague-Dawley rat liver S9-mix or Aroclor-induced golden Syrian hamster liver S9-mix (final volume 500 ; S9 mix/plate)	10%, Moltox 4050	30 min	50–5000 µg/plate

### Metabolic activation system

Phenobarbital and 5,6-benzoflavone induced Sprague–Dawley rat liver post-mitochondrial fraction (S9) or Aroclor-1254 induced Golden Syrian hamster liver post-mitochondrial fraction (S9) purchased from Molecular Toxicology Incorporated, NC, USA (MolTox™) was used as an exogenous metabolizing system. Sprague–Dawley rats were prepared using the treatment schedule described in Matsushima *et al*. [35], while Golden Syrian hamsters were prepared as described in ASTM E1687 [36]. The batches of S9 fraction were thawed immediately prior to use and prepared as detailed in Supplementary Table S1, with an NADPH generating system (which included NADP and glucose-6-phosphate). The final S9 mix contained 10% (v/v) of S9-liver fraction only, as a review of the NTP database did not reveal any uniquely positive NAs in the Ames test using 30% (v/v) S9 (data not presented).

Cytochrome P450 (CYP) 2E1 activity of S9 fractions (prepared from uninduced, phenobarbital, and Aroclor-1254 induced rat liver, and uninduced and Aroclor-1254 induced Golden Syrian hamster liver (Moltox™) was determined. An incubation mix consisting of S9 fraction (final protein concentration of 0.65 mg/ml) in 50 mM potassium phosphate buffer, pH 7.4 was pre-warmed with chlorzoxazone (CYP2E1 probe substrate, final concentration of 10 µM) prior to incubation at approximately 37°C, in triplicate, over a 30-min time-course in the presence of an NADPH-regenerating system (5.5 mM glucose-6-phosphate, 0.44 mM NADP and 1.2 units/ml of glucose-6-phosphate dehydrogenase in 2% sodium bicarbonate solution). Reactions were terminated after 0, 2, 6, 12, 18, and 30 min by removal of an aliquot of the incubation sample into ice-cold acetonitrile containing an internal standard ((^13^C_6_)-6-hydroxychlorzoxazone). Following centrifugation, supernatant was monitored for the 6-hydroxy metabolite of chlorzoxazone, against a calibration line, using a Linear Ion Trap Quadrupole LC/MS/MS mass spectrometer (AB Sciex, Toronto, Canada) and rate of metabolite formation plotted. Appropriate control incubations demonstrated the suitability of the assay to measure CYP2E1 activity.

The effect of vehicle on the activity of CYP2E1 of S9 fractions (prepared from phenobarbital and Aroclor-1254 induced rat liver, and Aroclor-1254 induced Golden Syrian hamster liver) was investigated. Incubations were prepared as described above, with the addition of vehicle [deionized water (control), methanol, DMSO, acetonitrile, or acetone], at final concentrations of 7.7% (v/v), prior to incubation at approximately 37°C for 30 min. Reactions were terminated after 0 and 30 min by removal of an aliquot of the incubation sample into ice-cold acetonitrile containing an internal standard ((^13^C_6_)-6-hydroxychlorzoxazone). Following centrifugation, supernatant was monitored for the 6-hydroxy metabolite of chlorzoxazone as described above, and percentage formation of metabolite compared to the control calculated.

### Bacterial strains

Five bacterial strains (*S. typhimurium* strains TA98, TA100, TA1535, TA1537, and *E. coli* strain WP2*uvrA* pKM101) were used to identify the mutagenic capacity of the selected NA compounds. The origin of the bacterial strains, along with strain genotype and type of mutation identified is summarised in Supplementary Table S2. Strains were maintained as frozen stocks with their genotype characteristics having been confirmed as described by Mortelmans and Zeiger [9]. Cultures used in the present investigation were prepared from these bacterial strain stock samples.

### Ames experimental conditions

The selective agar plates were purchased from E&O Laboratories, product number PP2037 (agar volume 24–26 ml). Bacteria were propagated from frozen stocks. The Ames test was conducted following the recommendations of the OECD 471 test guideline [22]. Bacteria were grown in nutrient broth with shaking (150 pm at 37°C) for 10 h until the exponential growth phase was reached and treatments were completed within 3 h from the end of incubation to ensure bacteria were in a stationary phase of growth. The ‘Ames’ plate incorporation and/or ‘Yahagi’ pre-incubation methods were selected for mutagenicity assessment, whereby bacterial suspension (100 µl), test compound or vehicle (100 µl for plate incorporation and 50 µl for pre-incubation) or positive control (100 µl) and S9 mix (or phosphate buffer when treated in the absence of S9-mix) (500 µl) were mixed and incubated. In the plate incorporation assay, these were mixed directly in 2 ml of histidine, biotin, and tryptophan-containing molten top agar (50°C) and the mixture was poured onto the selective agar plates and incubated for 3 days at 37°C. In the pre-incubation method, mixtures without top agar were placed into a shaking incubator for 30 min at 37°C. After the preincubation, 2 ml of histidine, biotin, and tryptophan-containing molten top agar were added, and the mixture was poured onto the selective agar plates. The plates were then incubated for 3 days at 37°C. Supplementary Table S3 lists the final volumes of each component for both the pre-incubation and plate incorporation assays. Counting of colonies was performed with the Ames Colony Counter and tables were generated using the Ames Study Manager software (Instem, UK). The experiments were conducted using three replicates for each concentration level, six replicates for vehicle control plates, and two replicates for positive control plates. Supplementary Table S4 lists positive controls for each bacterial strain (including concentrations), both in the presence and absence of S9-mix. Five to seven concentration levels were evaluated per study arm. The test concentrations ranged from 5 to 5000 μg/plate. For evaluation of treatment-related effects, a fold increase was defined as positive for mutagenicity, if a biologically relevant increase in the mean number of revertant colonies above a threshold of 2-fold (TA98, TA100, WP2 uvrA) or 3-fold (TA1535, TA1537) as compared to the concurrent negative controls was observed. Toxicity was assessed by eye at the end of the incubation period (72 h) and was determined by examination of the final bacterial population on the agar plates. Test articles were deemed toxic if there was ‘thinning’ or complete absence of the background lawn, or noteworthy reductions in revertant count, compared to the vehicle control.

### Benchmark dose (BMD) modelling

Benchmark dose analyses were carried out using the freely available PROAST R-package (version 70.3) (http://www.proast.nl). Ames test dose–response data (with cytotoxic test concentrations removed) were analysed using the exponential model family recommended by EFSA for the assessment of continuous (geno)toxicity dose–response data [37]. Where combined analyses were used, dose–response relationships were analysed using the assay conditions discriminating the subgroups (i.e. sub-study) as a covariate. To prevent overfitting, more complex models with additional parameters were only accepted if the goodness-of-fit exceeded the critical value at *P* < .05. PROAST outputs designate potency (i.e. the benchmark dose (BMD)) and its two-sided, 90% confidence interval (i.e. the BMDL and BMDU) as the ‘Critical Effect Dose’ (i.e. yielding a CED, CEDL, and CEDU), respectively, for each level of the covariate. Using the combined-covariate BMD approach [38], the model parameters that required estimation for each subgroup and those that could be considered constant across subgroups were established for the combined dataset. In general, the combined analyses presented here assumed that parameters for the maximum response (parameter *c*), log-steepness (parameter *d*) were equal for all subgroups, while the background response (parameter *a*), potency (parameter *b*), and within-group variation (*var*) were tested for subgroup dependence [39]. Model fits were used to visually evaluate the validity of the assumption of conserved shape. This approach was preferred to statistical testing as tests on shape parameters have been shown to be overly sensitive to non-random errors that are ubiquitous in experimental data [39]. Importantly, even minor non-random errors in the data could lead to the rejection of shape parameter constancy due to the relatively high statistical power in a combined dataset. However, minor differences among shape parameters between subgroups can only at most have a small impact on BMD confidence interval coverage. The benchmark response (BMR) (i.e. termed the critical effect size or CES in the PROAST software) used was 100% (i.e. a 2-fold increase in the revertant count relative to concurrent control). The BMDL and BMDU values represent the lower and upper bounds of the two-sided 90% confidence interval of the BMD, respectively, with the difference between the BMDU and the BMDL defining the width of the confidence interval, and therefore, its precision. Confidence interval plots, rank-ordered by BMD were employed to visually compare potencies across different study designs whilst taking estimation uncertainty into account [38]. Quantile–quantile plots and plots of residuals against dose were used to confirm approximate log-normality and variance homogeneity.

### Radar plotting maximum fold changes

To draw the radar plots, the maximum response (in terms of fold-change against concurrent control) per study design (i.e. compound, vehicle, S9 condition, and strain combination) was identified—regardless of the test concentration at which it occurred. Radar plots were then created using the MATLAB R2022b programming environment (MathWorks, MA, USA) using the freely available ‘radar plot’ function [https://uk.mathworks.com/matlabcentral/fileexchange/59561-spider_plot]. Close to the origin, dashed and solid red lines were added to represent the level of the 2 or 3-fold responses used to determine strain-specific positive calls. For each study design, coloured asterisks were added around the outside of plot to indicate experiment design combinations resulting in positive calls. A 2-fold or greater response with the TA100, TA98, or WP2uvrA (pKM101) strains was termed positive whereas a 3-fold or greater response was termed positive using strains TA1535 and TA1537.

## Results

### Impact of vehicle solvents on rat and Hamster liver S9 CYP2E1 activity

**Figure 1.**
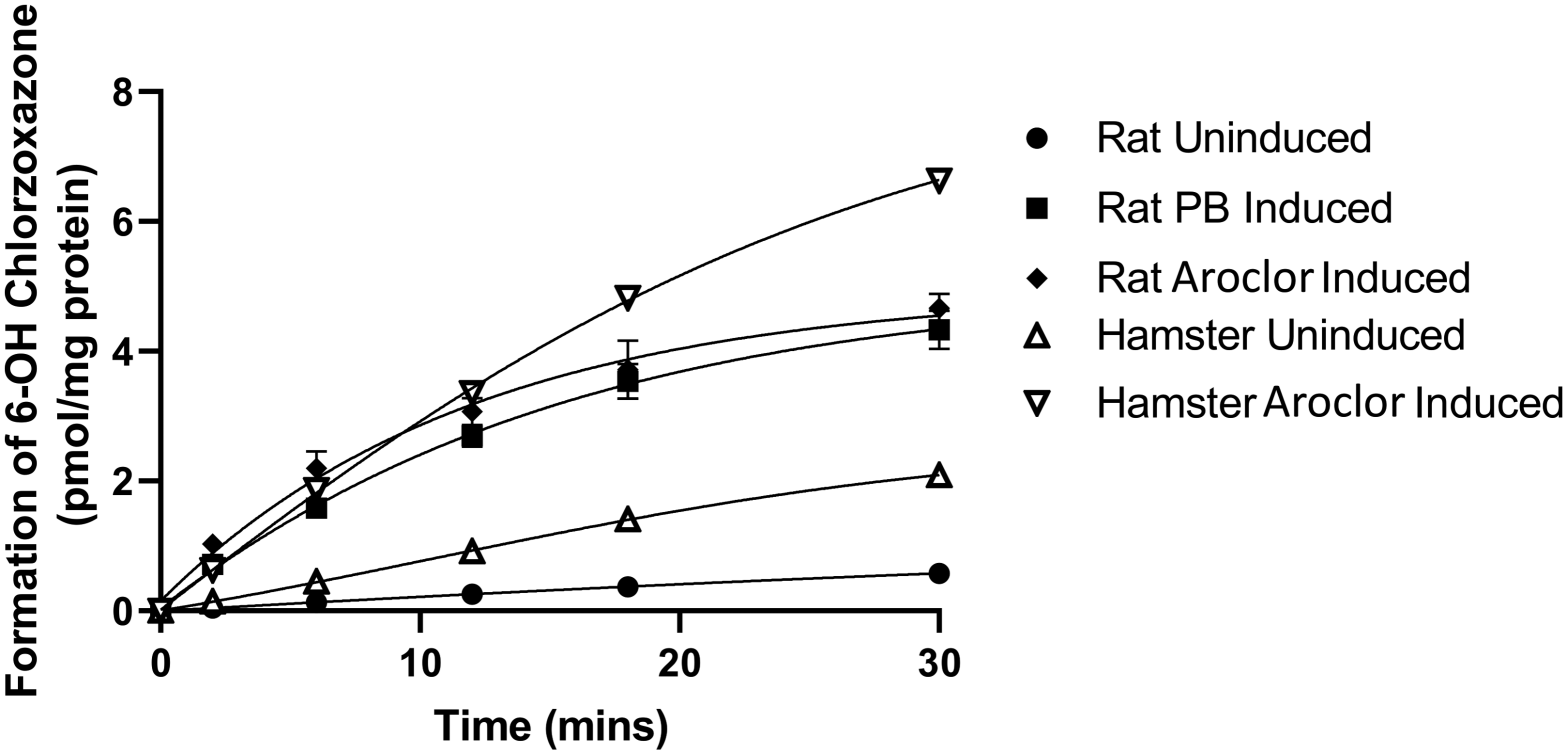
Metabolism of the CYP2E1 probe substrate chlorzoxazone to 6-OH chlorzoxazone by rat and hamster liver S9 fractions. Incubation of induced and uninduced rat and hamster liver S9 fractions with chlorzoxazone (CYP2E1 substrate). Formation of chlorzoxazone metabolite, 6-hydroxychlorzoxazone, was monitored by LC/MS/MS and measured over a time-course from 0 to 30 min.

**Figure 2.**
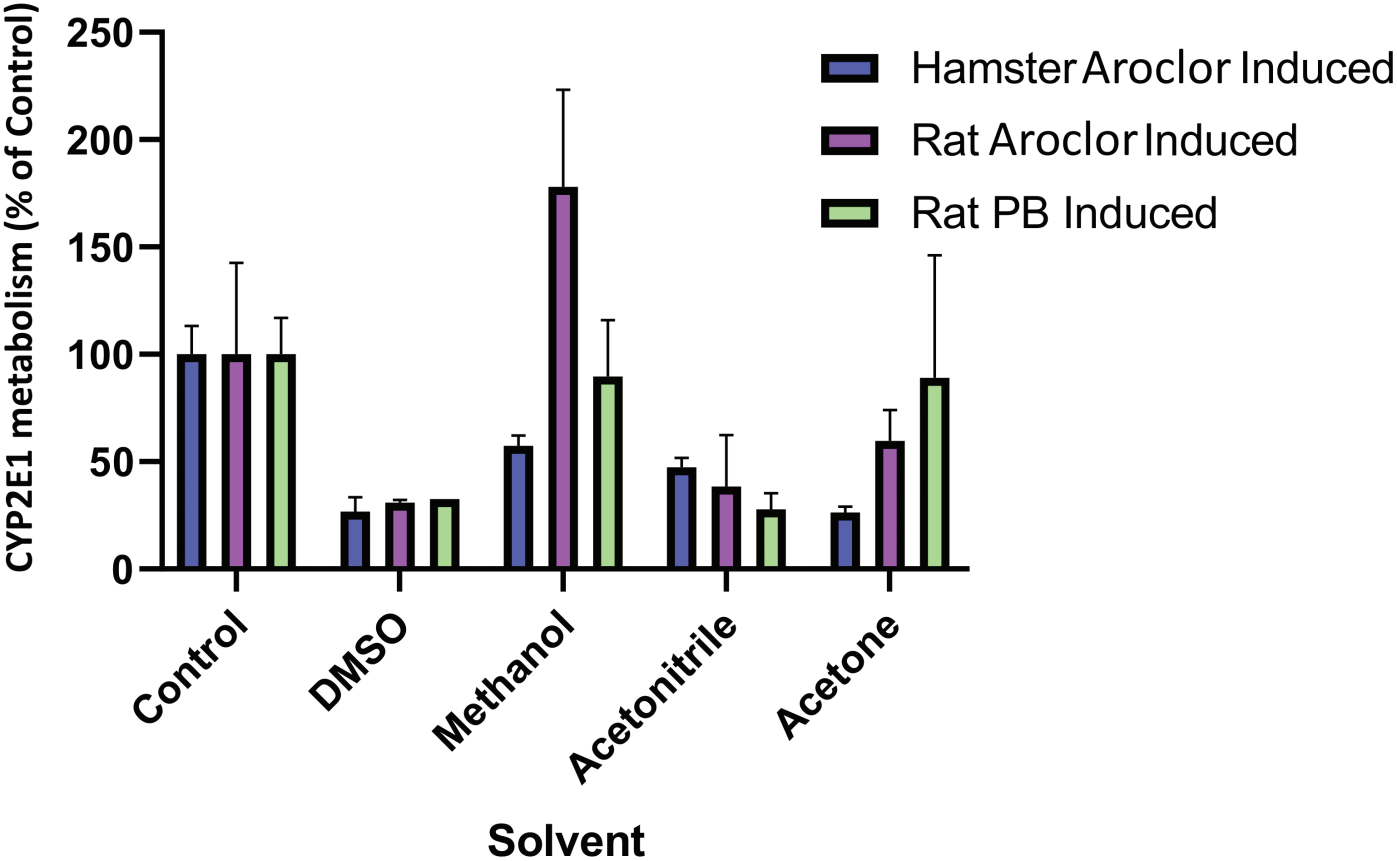
Effect of vehicle solvent on the metabolism of the CYP2E1 probe substrate chlorzoxazone to 6-OH chlorzoxazone by rat and hamster liver S9 fractions. Incubation of induced rat and hamster liver S9 fractions with chlorzoxazone (CYP2E1 substrate) and a final incubation concentration of 7.7% (v/v) of vehicle (DMSO, methanol, acetonitrile, and acetone). Formation of chlorzoxazone metabolite, 6-hydroxychlorzoxazone, was monitored by LC/MS/MS and the concentration formed determined as a percentage of concentration formed in control incubations.

Prior to mutagenesis testing, CYP2E1 activity was determined in induced rat and hamster liver S9 fractions typically used in the Ames test and compared with non-induced liver via the conversion of chlorzoxazone (a CYP2E1 substrate) to 6-hydroxychlorzoxazone. The formation of 6-hydroxychlorzoxazone (CYP2E1 substrate) was observed in all rat and hamster liver S9 fractions investigated and the rate of production of metabolite was significantly higher in induced S9 fractions compared with non-induced S9 in both species (Fig. 1). The addition of organic solvents DMSO and acetonitrile (final concentration 7.7%) was shown to inhibit the conversion of chlorzoxazone to 6-hydroxychlorzoxazone in rat and hamster-induced liver S9 compared with water. Methanol and acetone had negligible effect on phenobarbital and 5,6-benzoflavone induced rat liver S9 but reduced 6-hydroxychlorzoxazone formation by ~50% and ~75%, respectively, using Aroclor-induced hamster liver S9 (Fig. 2). This suggests CYP2E1 enzyme activity can be inhibited by various organic solvent vehicles in these liver preparations compared with water.

### The Ames test results

#### Plate incorporation method

The performance of the standard plate incorporation assay was investigated using NDMA and NDEA treatment. Standard five strain assays were performed with either rat or hamster-induced liver S9 and water, methanol, or DMSO as the vehicle. Negative (vehicle only) and positive control treatments resulted in strain-specific revertant colony frequencies consistent with the laboratory historical control values and thus confirmed assay validity. A high-level summary of the overall outcomes and Ames test conditions evaluated with NDMA and NDEA are presented in Table 2, whereas detailed study information and data, that is, revertant colony count per replicate plate (including treatment, vehicle, and positive controls) are available in the Supplementary Table S5.

**Table 2.** A summary of plate incorporation Ames test results with NDMA and NDEA.

Compound^a^ (CAS)	S9 species (induction)	Vehicle	Strain S typhimurium or E coli^*^					Result
			TA98	TA100	TA1537	TA1535	WP2*	
NDMA (62-75-9)	None	DMSO	−	−	−	−	−	Negative
		Methanol	−	−	−	−	−	Negative
		Water	−	−	−	−	−	Negative
	Rat	DMSO	−	−	−	−	−	Negative
		Methanol	−	−	−	−	+	Positive
		Water	−	−	−	−	+	Positive
	Hamster	DMSO	−	+	−	+	+	Positive
		Methanol	−	+	−	+	+	Positive
		Water	−	+	−	+	+	Positive
NDEA (55-18-5)	Rat	DMSO	−	−	−	−	+	Positive
		Methanol	−	−	−	−	+	Positive
		Water	−	−	−	−	+	Positive
	Hamster	DMSO	+	+	−	+	+	Positive
		Methanol	+	+	−	+	+	Positive
		Water	+	+	−	+	+	Positive

^a^Tested up to 5000 µg/plate, the maximum concentration in accordance with current guidelines.

^*^ *E. coli* strain WP2uvrA (pk101).

#### NDMA

In the absence of S9-mix, NDMA was not mutagenic in any bacterial strain in a plate incorporation assay when using water, methanol, or DMSO as vehicle, confirming metabolic activation is required to elicit a mutagenic response (Supplementary Fig. S1). Treatment with NDMA in the presence of rat liver S9-mix resulted in an increase in revertant colonies compared with vehicle controls, but only in *E.coli* WP2uvrA (pKM101) @ 1500 µg/plate and above using water as the vehicle, or with methanol at the limit concentration of 5000 µg/plate. When DMSO was used as the vehicle, NDMA was not positive in any strain at any concentration tested (Supplementary Fig. S2). In the presence of hamster liver S9-mix, NDMA induced positive increases in mutant frequency in *Salmonella* bacterial strains TA100, TA1535, and *E.coli* WP2uvrA (pKM101), with all three vehicles—water, methanol, or DMSO (Supplementary Fig. S3).

#### *N*-nitrosodiethylamine (NDEA)

Treatment with NDEA in the presence of rat liver S9-mix resulted in increased revertant colonies compared with vehicle controls, but again only in *E.coli* WP2uvrA (pKM101) with all three vehicles—water, methanol, or DMSO. In this strain, there were concentration-dependent increases in mutant frequency with all three vehicles from 1500 µg/plate and above, resulting in fold-change increases of similar magnitude at equivalent concentrations compared with the vehicle controls (Supplementary Fig, S4). Following the substitution of rat liver S9-mix with hamster liver S9-mix, NDEA-induced concentration-dependent increases in mutant frequency in *Salmonella* bacterial strains TA100, TA1535, TA98, and *E.coli* WP2uvrA (pKM101) with all three vehicles—water, methanol, or DMSO. The magnitude of mutant colony increases was dependent on strain, vehicle, and concentration of NDEA/plate (Supplementary Fig. S5).

**Figure 3.**
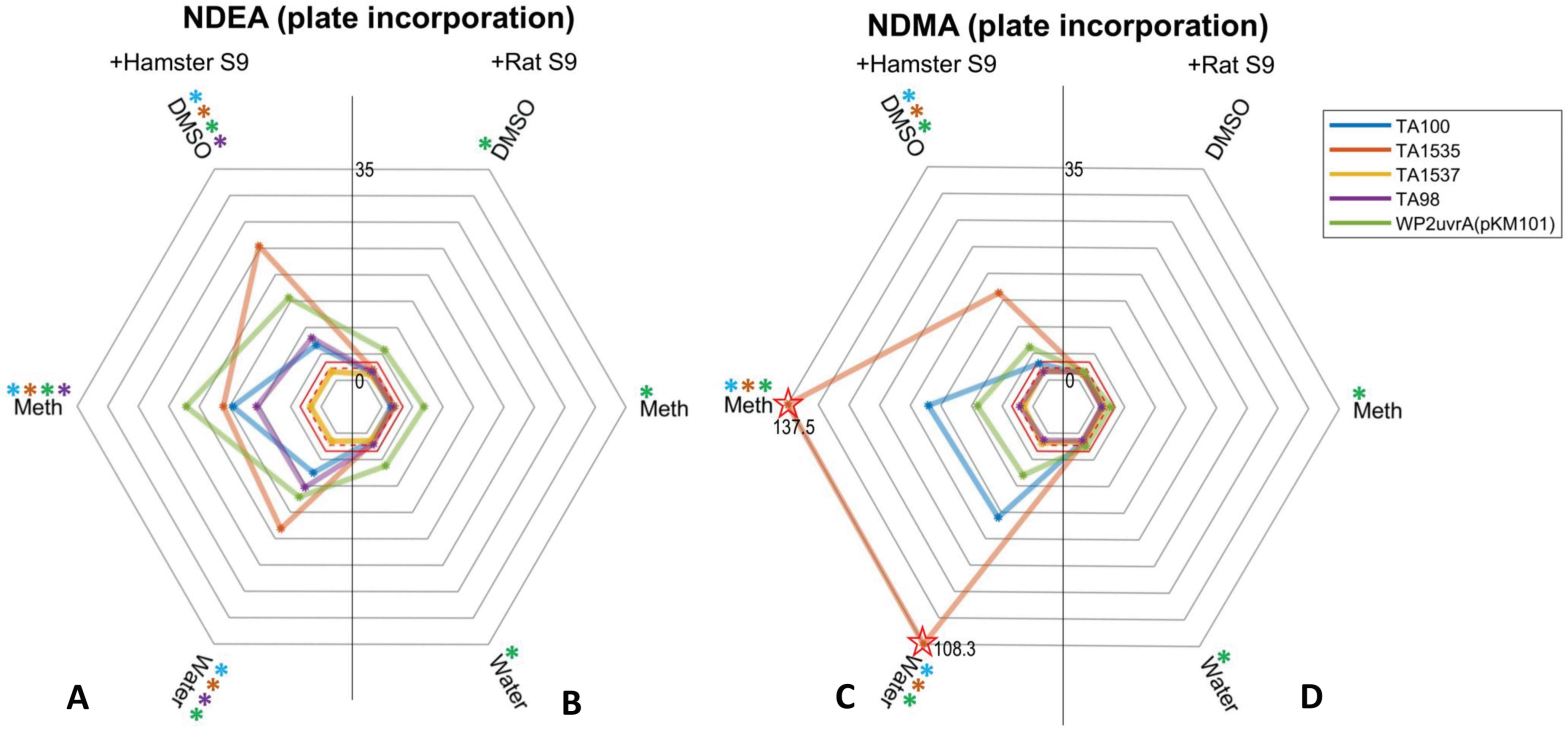
Plate incorporation maximum fold-change radar-plots of mutagenicity. (A–D) The plots show the highest revertant count ratio (i.e. treated response over vehicle control response) for every bacterial strain and vehicle combination for (A) NDEA and hamster liver S9, (B) NDEA and rat liver S9, (C) NDMA and hamster liver S9, or (D) NDMA and rat liver S9. An asterisk (*) represents a positive response when using that vehicle with the corresponding strain. Dotted and dashed lines indicate points of a 2 or 3-fold increases, respectively. Starred datapoints indicate where broken axes have been used to accommodate fold-change values above 35. In these instances, the fold-change is indicated on the plot (Meth = methanol).

To compare the impact of bacterial strain, vehicle, and S9 species selection in the plate incorporation method following treatment with NDMA and NDEA the maximum revertant count ratios for each combination were visualized using radar-plots (Figure 3). This figure demonstrates the superiority of hamster liver S9 over rat liver S9 in the detection of NDMA and NDEA mutagenicity, both in terms of magnitude of response and number of bacterial strains that are positive (represented by coloured asterisks above the vehicle type in the figure). When hamster liver S9 is used, the radar plots also indicate the magnitude of responses are similar when either water or methanol are used as vehicles with NDMA, and that these vehicles are superior to DMSO, whereas with NDEA, the magnitude of responses are similar for all three vehicles. The data confirm that an OECD-compliant standard plate incorporation method of the Ames test detects NDEA and NDMA mutagenicity, consistent with the conclusions of Trejo-Martin *et al*. [27], and that hamster liver S9 is superior to rat liver S9-mix for these small aliphatic NAs.

#### Pre-incubation method

To further investigate the impact of rat versus hamster-induced liver S9 metabolizing systems, NDEA, and NDMA were also tested in the pre-incubation (Yahagi) version of the Ames test [40]. In addition, because of the potential for organic solvents to inhibit cytochrome P450 metabolism the effect of solvent vehicle selection on the ability of the pre-incubation Ames test method to detect NDEA and NDMA mutagenicity was also investigated using DMSO, methanol, water, NMP, acetone, acetonitrile, DHF, and DMF as vehicle. Negative (vehicle only) and positive controls resulted in strain-specific revertant colony frequencies consistent with the laboratory historical control values and thus confirmed assay validity. A summary of the overall outcomes and test conditions evaluated are presented in Table 3, with complete study information and data provided in Supplementary Table S5.

**Table 3.** A summary of pre−incubation Ames test results with NDMA and NDEA.

Compound^a^ (CAS)	S9 species (induction)	Vehicle	Strain S typhimurium or E coli*					Result
			TA98	TA100	TA1537	TA1535	WP2*	
NDMA (62-75-9)	Rat	DMSO	−	−	−	−	−	Negative
		Methanol	−	−	−	−	+	Positive
		Water	−	−	−	−	+	Positive
		NMP	−	−	−	−	−	Negative
		Acetone	−	−	−	−	−	Negative
		Acetonitrile	−	−	−	−	−	Negative
		DHF	−	−	−	−	−	Negative
		DMF	−	−	−	−	−	Negative
	Hamster	DMSO	−	+	−	−	+	Positive
		Methanol	−	+	−	+	+	Positive
		Water	−	+	−	+	+	Positive
		NMP	−	−	−	−	−	Negative
		Acetone	−	+	−	+	+	Positive
		Acetonitrile	−	+	−	+	+	Positive
		DHF	−	+	−	−	−	Positive
		DMF	−	−	−	−	−	Negative
NDEA (55-18-5)	Rat	DMSO	−	−	−	−	+	Positive
		Methanol	−	−	−	−	+	Positive
		Water	−	−	−	−	+	Positive
		NMP	−	−	−	−	−	Negative
		Acetone	−	−	−	+	−	Positive
		Acetonitrile	Toxic	−	−	−	+	Positive
		DHF	−	+	−	−	−	Positive
		DMF	−	−	−	−	−	Negative
	Hamster	DMSO	+	+	−	+	+	Positive
		Methanol	+	+	−	+	+	Positive
		Water	+	+	−	+	+	Positive
		NMP	−	−	−	−	+	Positive
		Acetone	−	+	−	+	+	Positive
		Acetonitrile	Toxic	−	Toxic	−	+	Positive
		DHF	−	+	−	−	−	Positive
		DMF	−	+	−	+	+	Positive

^a^Tested up to 5000 µg/plate, the maximum concentration in accordance with current guidelines.

^*^ *E. coli* strain WP2uvrA (pk101).

### *N*-nitrosodimethylamine (NDMA)

Using rat liver S9-metabolic activation, positive results were observed only in bacterial strain *E. coli* WP2uvrA (pKM101), when either water or methanol was used as the vehicle (Supplementary Fig. S6). At the limit concentration (5000 µg/plate), NDMA treatment resulted in a 2.6-fold (water) and 2-fold (methanol) increase in mutant frequency, respectively, compared with vehicle controls. However, in the presence of hamster liver S9-mix, significant concentration-related increases in mutant frequency were noted in *Salmonella* strains TA100, TA1535, and *E. coli* WP2uvrA (pKM101) using water and methanol as vehicles. Positive responses were also seen with other organic solvent vehicles, including DMSO, acetone, acetonitrile, and DHF (Supplementary Fig. S7).

### *N*-nitrosodiethylamine (NDEA)

NDEA was positive using rat liver S9-mix, with *E. coli* WP2uvrA (pKM101) as the most sensitive strain (when using DMSO, methanol, water, or acetonitrile as vehicles), however, positive responses were also observed using *Salmonella* strains TA1535 (acetone) and TA100 (DHF) (Supplementary Fig. S8). In the presence of hamster liver S9-mix, NDEA was positive irrespective of vehicle (Supplementary Fig. S9). Once again, *E. coli* WP2uvrA (pKM101) was the most sensitive strain and was positive with every vehicle except DHF. However, NDEA was positive in *Salmonella* strain TA100 when using DHF as the vehicle. NDEA was also positive in other *Salmonella* strains (TA100, TA1535, and TA98) and across a range of organic solvent vehicles.

**Figure 4.**
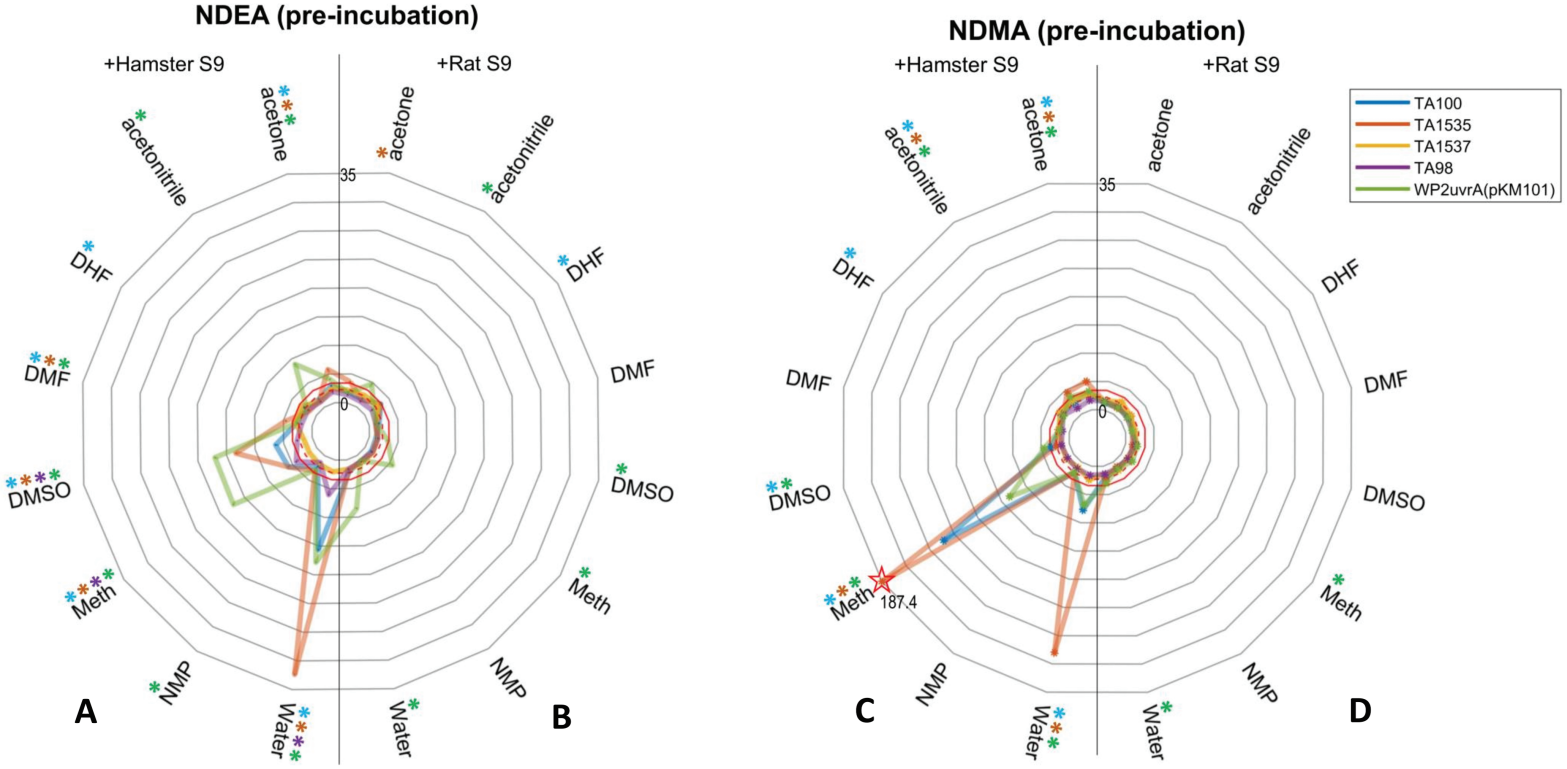
Pre-incubation maximum fold-change radar-plots. (A–D) The plots show the highest revertant count ratio (i.e. treated response over vehicle control response) for every bacterial strain and vehicle combination for (A) NDEA and hamster liver S9, (B) NDEA and liver rat S9, (C) NDMA and liver hamster S9, or (D) NDMA and rat liver S9. An asterisk (*) represents a positive response when using that vehicle with the corresponding strain. Dotted and dashed lines indicate points of a 2 or 3-fold increase, respectively. Starred datapoints indicate where broken axes have been used to accommodate fold-change values above 35. In these instances, the fold-change is indicated on the plot (Meth = methanol).

Again, Fig. 4 shows the maximum induced mean revertant count ratios for NDMA and NDEA for each combination of test conditions. Once more, this demonstrated the superiority of induced hamster liver S9 over induced rat liver S9 for the generation of NDMA and NDEA mutagenicity, both in terms of magnitude of response and number of bacterial strains that are positive. There were noteworthy potency differences with hamster liver S9, compared to rat liver S9-mix, especially when water or methanol was used as the vehicle with NDMA, and there was little to differentiate among water, methanol, or DMSO with NDEA.

Overall, the results from the pre-incubation assay qualitatively demonstrate the superiority of hamster liver S9-mix to detect the mutagenicity of NDEA and NDMA compared with rat liver S9-mix. In addition, the data indicate that mutagenic activity is detected with a range of organic solvent vehicles and that there is little to differentiate water and methanol in terms of mutagenic potency.

### Bench-mark dose derived potency rankings

To further understand the impact of different test parameters on test outcomes, BMD-derived potency/sensitivity rankings [38] were generated from the dose–response relationships for NDMA and NDEA. In this approach, the ‘benchmark dose’ that can be expected to elicit a predetermined change in response (the benchmark response or critical effect size; here a 2-fold doubling of the response in the vehicle controls) is estimated and plotted as a confidence interval. Put simply, the dose that can be expected to elicit this response most likely lies within the confidence interval and so in turn, the confidence intervals can be ‘potency ranked’ from lowest to highest BMD (i.e. most-to-least potent/sensitive). Where BMD confidence intervals do not overlap, we can reliably conclude that differences in potency are significant. In contrast, where confidence intervals do overlap, the conclusion is that the underlying dose–response data do not contain sufficient information to resolve differences in potency. Indeed at some point where intervals overlap and occupy a narrow range, the approach may support conclusions of equi-potency/equi-sensitivity, etc. At this point, BMD-derived potency rankings have been widely used to compare potencies across different covariates (e.g. combinations of assay conditions) for many endpoints and the approach is considered ‘ready now’ by GTTC/IWGT [41–43]. In proceeding in this way with Ames test data, it should be noted that we do not propose the use of the BMD method to make positive or negative calls on the dose–response data. Similarly, we are not suggesting these values have utility as points-of-departure metrics (because the Ames test is primarily a bacterial hazard identification system). Instead, the purpose is to *quantitatively* provide a means to compare the potency/sensitivity of different assay conditions, that is, bacterial strain, S9 source, vehicle, and plate/pre-incubation methods, to inform on optimal study designs for robust/sensitive mutagenicity calls. In this way, dose–response data were only taken forward for BMD-modelling for conditions yielding a positive call, without overt bacterial toxicity under standard assay criteria.

**Figure 5.**
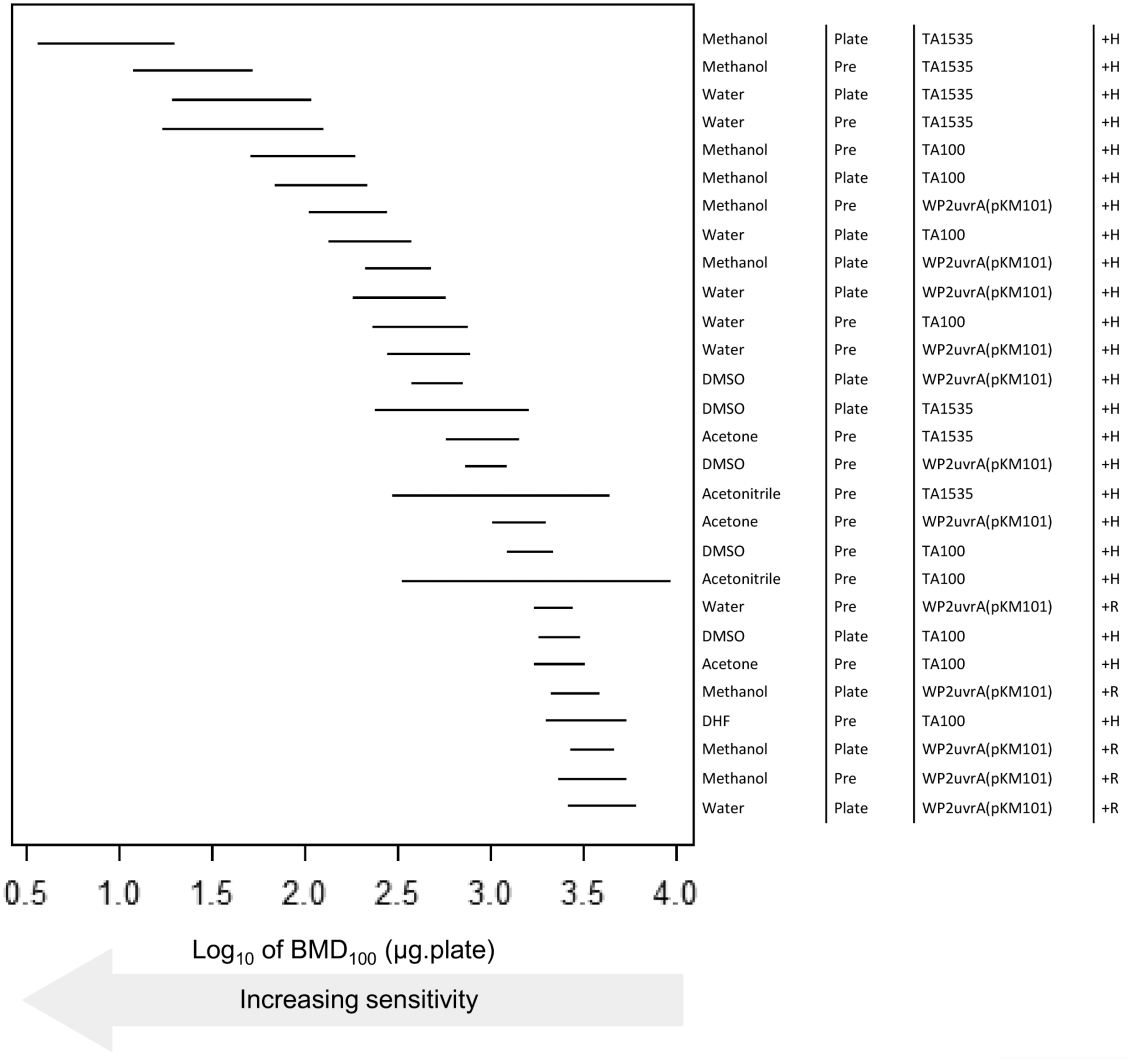
BMD-derived sensitivity rankings for NDMA derived using Ames test dose–response data collected under different experimental conditions. The left-panel shows the two-sided 90% confidence interval (CI) for the BMD_100_ for each combination of vehicle, method (i.e. pre or plate incorporation), strain, and metabolic activation (S9) source (described, right-hand side). In essence, the lines describe the dose–range mostly likely to cause a 2-fold doubling of the response in the vehicle controls, and therefore the lower (i.e. increasingly left-shifted) the confidence interval the more potent (i.e. sensitive) the response. The upper portion of the ranking is dominated by tests using water or methanol as vehicle, with TA1535, TA100, or WP2uvrA(pKM101) strains and metabolic activation using Hamster S9. The BMD analyses, underlying dose–response data and fitted model curves are shown in Supplementary Fig. S10 (A–D).

For NDMA, 28 positive dose–response datasets were analysed. The results suggest these positive studies exhibit in the region of a ~1000-fold range (three log units) in terms of sensitivity/potency (Fig. 5) The datasets with the highest sensitivity/potency (i.e. lowest BMDs) share specific similarities in study parameter composition. They always use hamster-induced liver S9 for metabolic activation and methanol or water was invariably the vehicle. In terms of bacterial strain, TA1535 was the most sensitive (occupying the four highest rankings), although TA100 and WP2uvrA (pKM101) were also present in the top quartile. Taken together, for NDMA, the combination of hamster liver S9 and methanol as a vehicle in *Salmonella* strain TA1535 exhibit the greatest sensitivity. When using these assay parameters, there appeared to be little difference in terms of the plate incorporation versus pre-incubation methods—which ranked #1 and #2, respectively, and exhibited overlapping BMD confidence intervals. The use of rat liver S9 in the plate incorporation method with *E. coli* strain WP2uvrA (pKM101) and water as the vehicle was the least sensitive combination, but nevertheless, still yielded a positive Ames test response. The modelling suggests that both plate incorporation and pre-incubation Ames test assay methods can achieve high sensitivity when paired with hamster liver S9 and the right choice of vehicle and bacterial strain.

**Figure 6.**
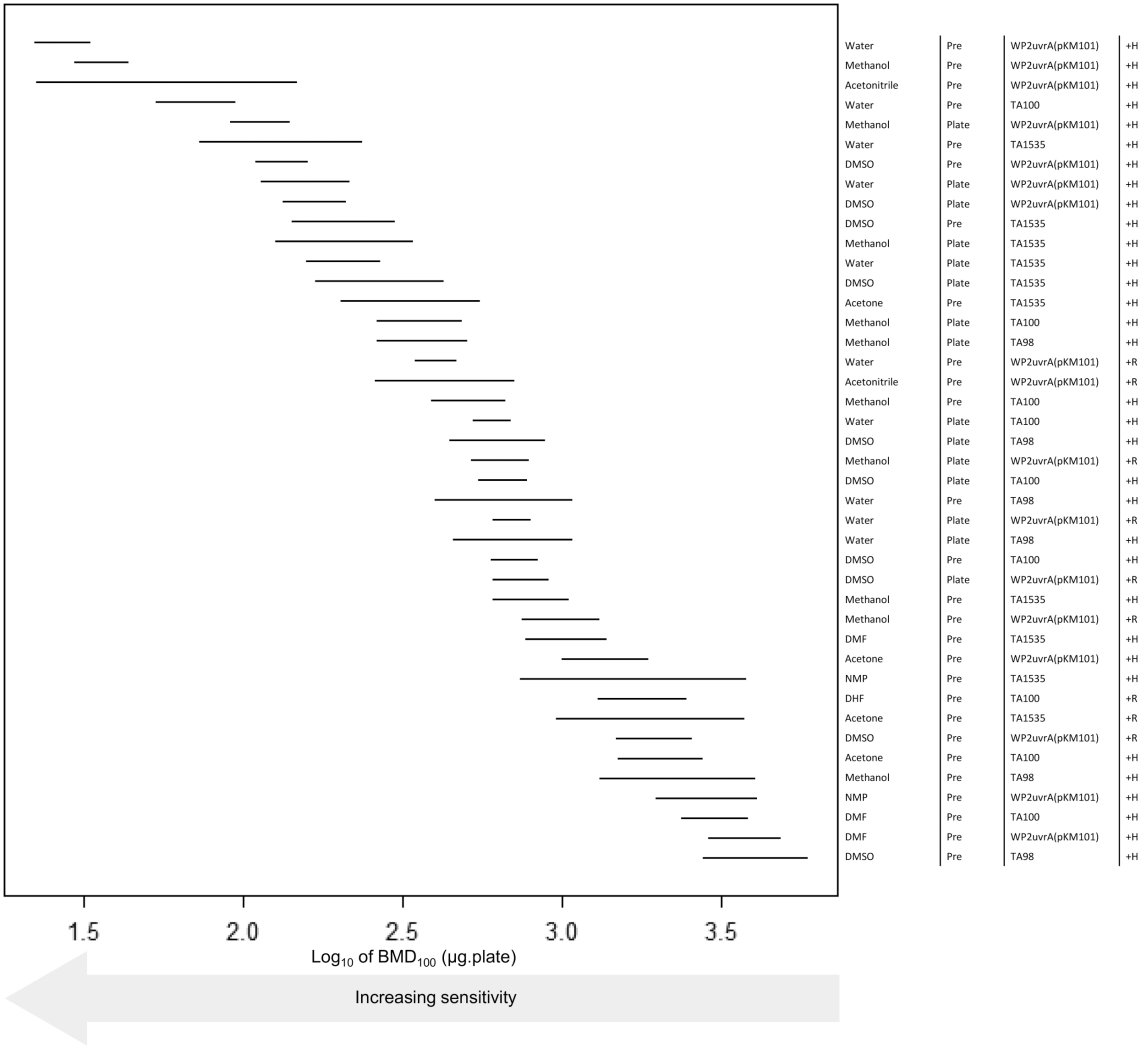
BMD-derived sensitivity rankings for NDEA derived using Ames test dose–response data collected under different experimental conditions. The left-panel shows the two-sided 90% confidence interval (CI) for the BMD_100_ for each combination of vehicle, method (i.e. pre or plate incorporation), strain, and metabolic activation (S9) source (described, right-hand side). The CIs describe the dose–range mostly likely to cause a 2-fold doubling of the response in the vehicle controls, and therefore the lower (i.e. increasingly left-shifted) the confidence interval the more potent (i.e. sensitive) the response. The upper portion of the ranking is occupied by tests using WP2uvrA(pKM101) strains with metabolic activation using Hamster S9. The BMD analyses, underlying dose–response data and fitted model curves are shown in Supplementary Fig, S11 (A-D).

For NDEA, 42 positive datasets were analysed. Here, the BMD confidence intervals occupied a ~100-fold range (~2 log units) in sensitivity/potency (Fig. 6) and there were more overlapping experimental conditions with similar mutagenic potency (i.e. overlapping BMD confidence intervals). In the upper quarter of the ranking, induced Hamster liver S9 was the only metabolic activation system represented, and water (4/11), methanol (3/11), and DMSO (3/11) were the most common vehicles. *E. coli* WP2uvrA (pKM101) was consistently the most sensitive bacterial strain (7/11) and occupied the three highest rankings, although *Salmonella* strains TA1535 (3/11) and TA100 (1/11) were also present in the top quartile. In terms of plate incorporation versus pre-incubation methods, the pre-incubation method was the most prevalent (7/11) and occupied the top four rankings overall. Taken together, for NDEA, the combination of hamster liver S9, *E. coli* WP2uvrA (pKM101), and pre-incubation provided the greatest sensitivity, and there appeared to be minor difference in terms of vehicle (with acetonitrile also in the top #3 rankings). Interestingly, acetonitrile and acetone were among the vehicles identified in the top half of the BMD sensitivity/potency rankings with NDEA, but it was noted that acetonitrile also induced toxicity in some arms of the Ames test study (Table 3).

The use of hamster liver S9 significantly increased sensitivity (i.e. lower test concentrations were required to elicit a 2-fold response), whilst rat liver S9 only appears in the top half of the potency rankings twice, based on the BMD confidence intervals, and even then, their position was lower down in the ‘pecking order’ (#17 and #18, respectively). Both instances were associated with the use of the pre-incubation method and *E. coli* WP2uvrA (pKM101) strain, but differed in the vehicle used, that is, water and acetonitrile, respectively. Taken together, for NDEA, hamster liver S9 with a pre-incubation method and *E. coli* strain WP2uvrA (pKM101) exhibited the greatest sensitivity, whilst hamster liver S9, in the pre-incubation method with, DMSO and *Salmonella* strain TA98 represented the least sensitive set of assay conditions still yielding a positive response.

Based on the BMD analysis with NDMA and NDEA, the following assay conditions consistently yielded the most potent responses: induced hamster liver S9, Ames test pre-incubation method, the inclusion of *Salmonella* strains TA1535 and TA100 in addition to the *E.coli* strain WP2uvrA (pKM101). Importantly, the choice of vehicle did not appear to be such a significant determinant. Water or methanol was generally superior to DMSO in terms of highest potency/sensitivity but not in terms of the overall detection of a positive result if paired with hamster liver S9 and the three sensitive bacterial strains (TA1535, TA100, and WP2uvrA (pKM101)) and using a pre-incubation method.

### NMEA

To confirm the performance of the Ames test using the most sensitive assay parameters identified, a further compound, *N*-nitrosomethylethylamine (NMEA) was evaluated. NMEA was chosen because it was identified as a discordant NA (i.e. one with a negative legacy Ames test and a positive rodent cancer bioassay). In this arm of the study, NMEA was tested in an expanded Ames test protocol using all five bacterial strains in the plate incorporation method (in the presence and absence of rat liver S9-mix) and in the pre-incubation (Yahagi) method (in the presence of rat liver S9-mix or hamster liver S9-mix). All study arms used DMSO, water, or methanol as vehicles, except for pre-incubation with Hamster liver S9, which was only evaluated with water or methanol as vehicles. A summary of the overall outcomes and test conditions evaluated are presented in Table 4, with full study information and data available in Supplementary Table S5.

**Table 4.** Summary of Ames test results for NMEA.

Compound^a^ (CAS)	Method	S9 species (induction)	Vehicle	Strain S typhimurium or E coli*					Result
				TA98	TA100	TA1537	TA1535	WP2*	
NMEA (10595-95-6)	Plate	None	Water	−	−	−	−	−	Negative
			DMSO	−	−	−	−	−	Negative
			Methanol	−	−	−	−	−	Negative
		Rat	Water	−	−	−	−	+	Positive
			DMSO	−	−	−	−	−	Negative
			Methanol	−	−	−	−	+	Positive
	Pre-inc	Rat	Water	−	−	−	−	+	Positive
			Methanol	−	+	−	−	+	Positive
			DMSO	−	−	−	−	+	Positive
		Hamster	Water	−	+	−	+	+	Positive
			Methanol	−	+	−	+	+	Positive

^a^The maximum concentration tested was 5000 µg/plate, the maximum concentration in accordance with current guidelines.

^*^ *E. coli* strain WP2uvrA (pk101).

#### Plate incorporation method

In the absence of S9-mix, NMEA was not mutagenic in any bacterial strain or combination of vehicle (Supplementary Fig. S12). In the presence of rat liver S9-mix, NMEA treatment resulted in a concentration-related increase in the numbers of revertant colonies per plate compared with vehicle controls, but only in *E. coli* WP2uvrA (pKM101), when either water or methanol was used as a vehicle (Supplementary Fig. S13). In methanol, NMEA produced a dose-related increase in mutant frequency, from 1500 µg/plate and above, reaching 4.3-fold at the limit concentration tested (5000 µg/plate), while in water, NMEA produced a dose–related increase from 2500 µg/plate, reaching 2.7-fold at the limit concentration.

#### Pre-incubation method

In the presence of rat liver S9-mix, NMEA treatment resulted in a concentration-related increase in revertant colonies compared with vehicle controls, in *E. coli* strain WP2uvrA (pKM101) when water or DMSO was used as a vehicle. NMEA was also positive at the limit concentration (5000 µg/plate) in methanol in *E. coli* strain WP2uvrA (pKM101) and resulted in concentration-related increase in *Salmonella* strain TA100 from 1500 µg/plate. In this arm of the study, fold-change increases were generally small (2–4-fold) and of similar magnitude at equivalent concentrations, irrespective of the vehicle used (Supplementary Fig. S14). When hamster liver S9-mix was used, NMEA induced robust, highly positive responses in *Salmonella* strains TA100 and TA1535, and was also positive in *E. coli* strain WP2uvrA (pKM101) when water was used as the vehicle. The fold-increases at the limit concentration of NMEA (5000 µg/plate) increased by 275-fold in water in *Salmonella* strain TA1535 and 32-fold in strain TA100. When water was substituted for methanol, NMEA still induced positive responses in *Salmonella* strains TA1535, TA100, and *E.coli* strain WP2uvrA (pKM101), although the magnitude of the responses was reduced (Supplementary Fig. S15).

**Figure 7.**
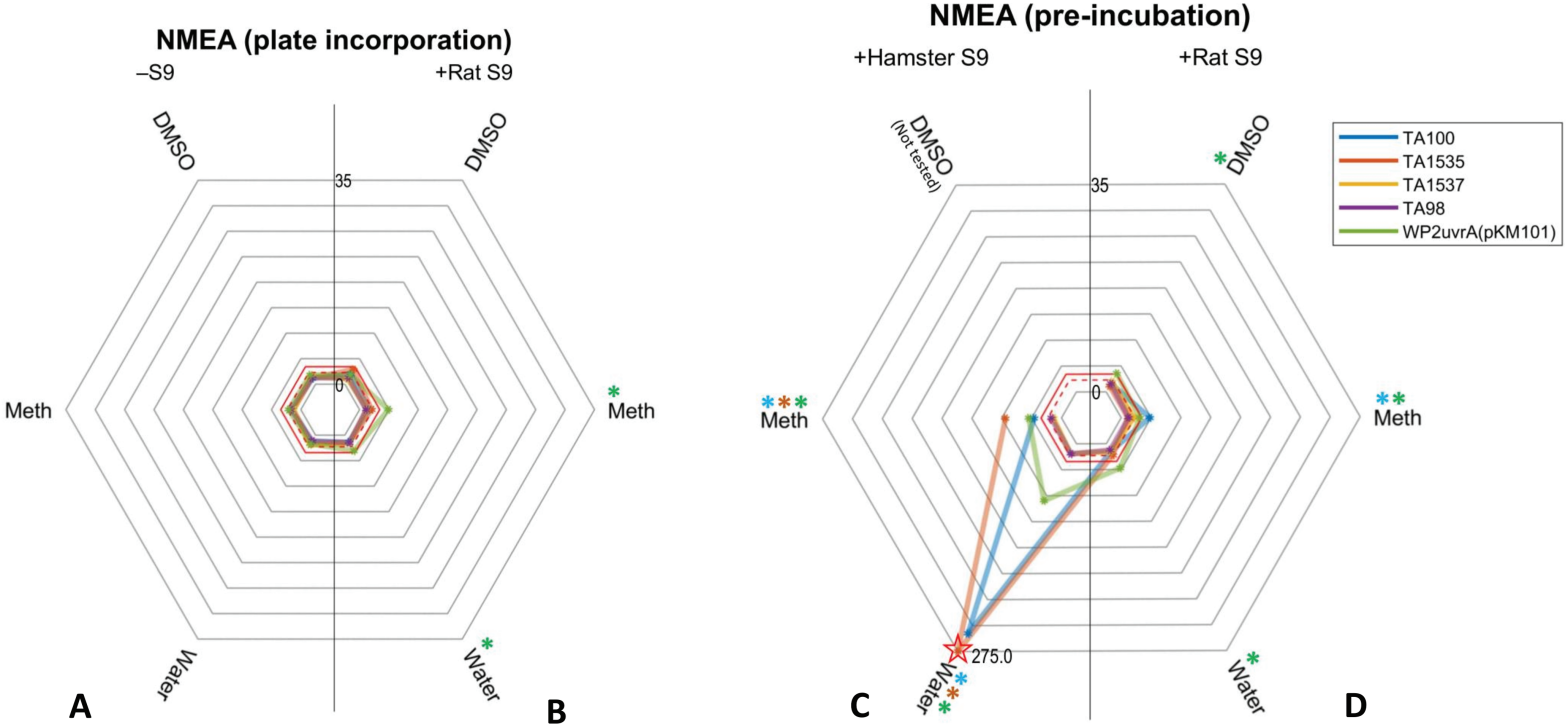
Plate incubation and pre-incubation maximum fold-change radar-plots of mutagenicity for NMEA. (A–D) The plots show the highest revertant count ratio (i.e. treated response over vehicle control response) for every bacterial strain and vehicle combination in the (A) absence of S9; (B) presence of rat liver S9, plate incorporation; (C) presence of hamster liver S9, pre-incubation or; (D) presence of rat liver S9, pre-incubation. An asterisk (*) represents a positive response when using that vehicle with the corresponding strain. Dotted and dashed lines indicate points of a 2 or 3-fold increase, respectively. Starred datapoints indicate where broken axes have been used to accommodate fold-change values above 35. In these instances, the fold-change is indicated on the plot (Meth = methanol).

**Figure 8.**
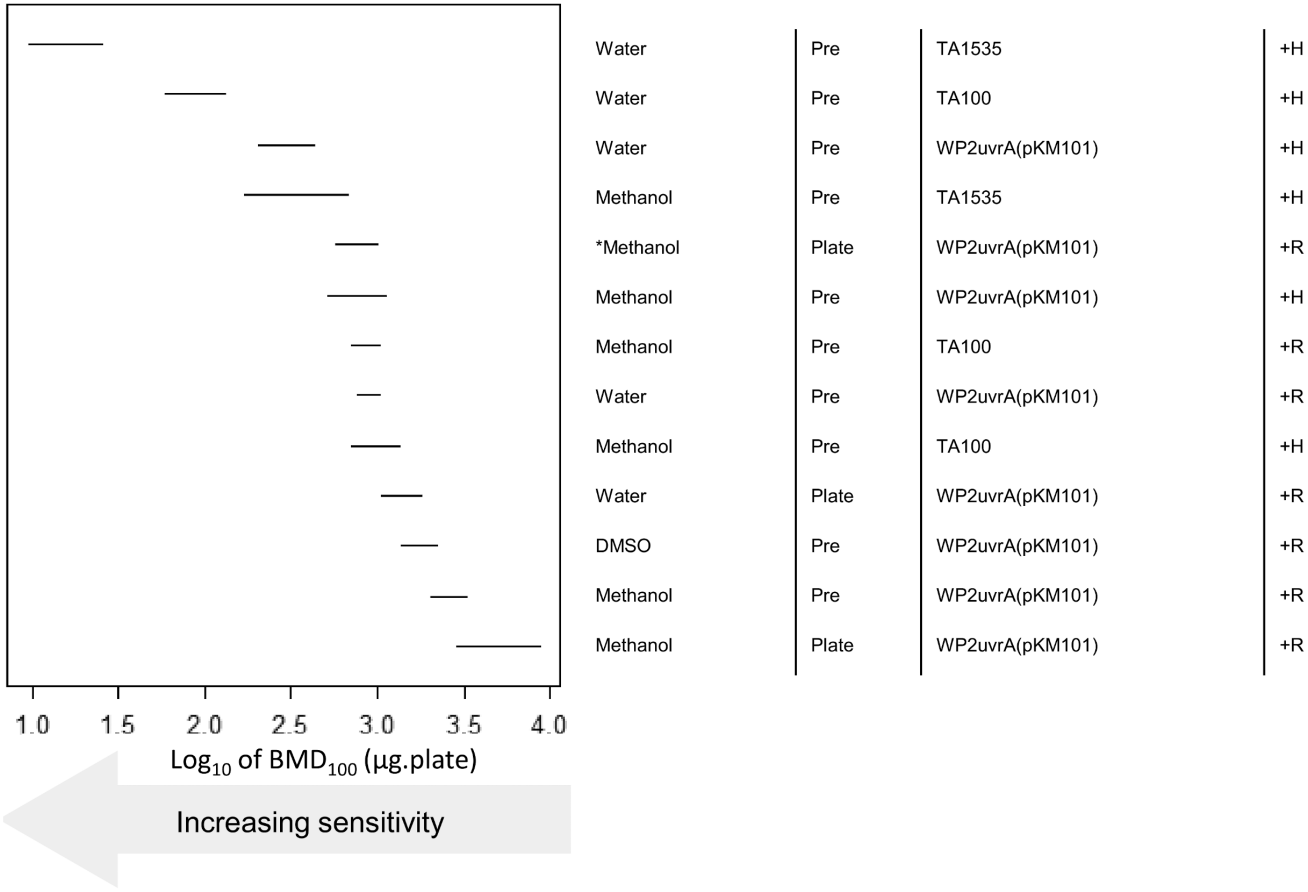
BMD-derived sensitivity rankings for NMEA derived using Ames test dose–response data collected under different experimental conditions. The left-panel shows the two-sided 90% confidence interval (CI) for the BMD_100_ for each combination of vehicle, method (i.e. pre or plate incorporation), strain, and metabolic activation (S9) source (described, right-hand side). The CIs describe the dose–range mostly likely to cause a 2-fold doubling of the response in the vehicle controls, and therefore the lower (i.e. increasingly left-shifted) the confidence interval the more potent (i.e. sensitive) the response. The upper portion of the ranking is predominantly occupied by tests using methanol or water as vehicle, the pre-incubation method and metabolic activation using Hamster S9. The BMD analyses, underlying dose-response data and fitted model curves are shown in Supplementary Fig. S16 (A–D). The asterisk (*) represents where testing was repeated (with the same conditions) to clarify the mutagenic response seen.

As before, the maximum NMEA-induced revertant count ratios for each of the test combinations were visualized using radar plots (Fig. 7). Again, the results demonstrate the superiority of hamster liver S9 over rat liver S9, and pre-incubation over plate incorporation, for the induction of mutagenicity in terms of magnitude of response and number of bacterial strains that are positive. There were significant potency differences when water was used as a vehicle, although methanol also produced positive results. BMD modelling of the positive NMEA datasets revealed a 1000-fold range in potency/sensitivity ranking (Fig. 8) with the lowest BMD confidence intervals (reflecting the most potent/sensitive responses) demonstrating similarities in several study parameters, that is, pre-incubation with hamster liver S9 and water occupying rankings #1 to #3. The use of methanol as a vehicle yielded responses that were not quite as potent as water yet methanol dominated the central quartile of the ranking demonstrating its suitability as a vehicle with NMEA. With respect to bacterial strain, *Salmonella* TA1535, TA100, and *E.coli* WP2uvrA (pKM101) were among the top three potency rankings, which was consistent with the observations uncovered with NDMA and NDEA.

## Discussion

NAs are generally considered potent genotoxic carcinogens in animals [44] and the majority require metabolic activation to their ultimate mutagenic species [45–47]. Due to their potency, NAs are among the ‘cohort of concern’ of chemicals in terms of regulatory control of DNA reactive (mutagenic) impurities in pharmaceuticals [4]. The reports of certain NAs as process impurities in some commercial drug products [48–50] have not only resulted in the withdrawal of several medicinal products from the market but also the publication of guidance regarding the detection and prevention of unacceptable levels of NA impurities in pharmaceutical products [51,52].

Under ICH M7, the Ames test is considered a ‘cornerstone’ assay for the assessment and control of DNA reactive (mutagenic) impurities in pharmaceuticals, because a negative test result has widely been used to discharge concern regarding the potential for genotoxic carcinogenesis. However, historical concerns regarding so-called discordant Nas, that is, Ames test negative and rodent cancer bioassay positive compounds [53] have undermined the use of the Ames test as part of the control strategy for this class of compound. The consequence is negative Ames test data alone are no longer considered sufficient by regulatory authorities to discharge the human safety concern regarding the carcinogenic potential of NA impurities. Therefore, the industry is faced with the situation whereby extensive follow-up testing is now required by default to support the risk assessment of NA impurities in pharmaceuticals in the absence of a NA-specific rodent cancer bioassay, and when the impurity cannot be purged during manufacture and/or adequately controlled to levels of ng per day in drug product. Whether the combination of a negative Ames test and rodent transgenic gene mutation assay can help to discharge concerns regarding the potential for genotoxic carcinogenicity in the absence of NA-specific rodent cancer bioassay is currently the topic of considerable research.

Generally, the Ames test is regarded as a sensitive and specific assay for predicting mutagenic rodent carcinogens [54], however, it has long been understood the assay is not always appropriate for certain substances, for example, antibiotics, or those compounds that are known (or thought) to interfere specifically with mammalian cell replication, for example, topoisomerase inhibitors [22]. In addition, there are some chemical classes, such as short-chain aliphatic NAs, divalent metals, aldehydes, azo-dyes, and diazo compounds, etc., which are known to exhibit lower potency when using the standard plate-incorporation Ames test [3,22] and the pre-incubation method has been recommended as an alternative [14]. The legacy performance of the Ames test with NAs has been cited as one of the reasons for regulatory authority concern. As a ‘cornerstone’ assay in pre-clinical testing, it is important for all stakeholders (regulatory authorities, industry, academia, and the public) to have confidence in the Ames test and trust negative assay outcomes for robust decision-making. Therefore, we have investigated the impact of various Ames test assay parameters, that is, bacterial strain, S9 species (rat vs. hamster liver) used for metabolic activation, plate incorporation and pre-incubation methods, and vehicle choice to investigate which conditions contribute the most to mutagenic potency, using NDMA and NDEA as exemplars.

We investigated the use of water, methanol, or DMSO as a vehicle in both the plate incorporation and pre-incubation methods, and a wider selection of organic solvent vehicles in the pre-incubation method. Qualitative and quantitative approaches were used to compare assay parameters and identify those that provided the most sensitive combination in terms of overall mutagenic potency. Based on the results with NDMA and NDEA, we used a selection of the most sensitive combination of Ames test assay parameters to re-investigate the mutagenicity of NMEA, a legacy Ames test negative dialkyl-NA [19] previously identified as a liver carcinogen in the rat, [34] that is, a discordant NA in terms of rodent cancer bioassay predictivity. Our results show that NMEA is positive in an OECD TG-471 compliant Ames test using an appropriate combination of assay parameters, and therefore should no longer be considered discordant. Our results are consistent with those published by Phillipson and Ioannides [32], using hamster or rat S9 in a preincubation assay and water as solvent, and the discordant results reported by Rao *et al*. [19] may simply reflect the limited bacterial strains and test concentration used on their study.

Using the results generated with NDMA, NDEA, and NMEA we considered each of the main Ames test assay parameters. CYP2E1 is an obligatory enzyme for the activation of small-alkyl-NAs [55] and prior to any Ames test work, we showed that rat and hamster liver S9 can convert the CYP2E1 probe substrate chlorzoxazone to 6-hydroxychlorzoxazone, confirming these liver fractions express functional CYP2E1. We also show that phenobarbital and benzoflavone or Aroclor-1254-induced rodent livers have considerably higher metabolic activity compared with non-induced liver. The rates of metabolic conversion of chlorzoxazone to 6-hydroxychlorzoxazone were higher in hamsters compared with rat, irrespective of liver induction—consistent with the idea that hamster liver has more CYP2E1 enzyme capacity than rat [32,56]. Benchmark dose modelling demonstrated induced hamster liver S9 activation was the assay parameter most singularly associated with high, overall mutagenic potency, and was clearly superior to induced rat liver S9 for the activation of NDMA, NDEA, and NMEA (most likely because of higher CYP2E1 metabolic capacity). These results are consistent with the published literature, which consistently demonstrates hamster liver S9 is more effective and generally superior in terms of the activation of many NAs to proximate mutagens, including NDMA and NDEA [25,26,32,57–59]. Despite this, our results confirm the Ames test using rat liver S9 is still capable of detecting mutagenicity induced by short alkyl-NAs, consistent with the observations of Bringezu and Simon [60]. However, positive results were observed only under certain assay conditions, and in all cases, the mutagenic potency was inferior to hamster activation, and ranked outside the top quartile for overall mutagenic potencies for all three compounds. Based on these results, the inclusion of hamster liver S9 in the evaluation of NAs in the Ames test is recommended to ensure increased assay sensitivity.

Historically, the pre-incubation method has been considered to have certain advantages over the plate-incorporation method. The close contact and interaction between the test compound, bacterial cells, and the metabolic milieu during the initial incubation period has been thought to reduce the possibility of non-specific binding with agar (as used in the plate-incorporation method) and result in higher bacterial exposures to ‘activated’ mutagens [61]. Furthermore, in the pre-incubation method, the bacterial growth lag-phase is limited (1–2 h), due to the nourishing effects of S9 protein [62], whereas in the plate-incorporation assay the lag-phase can exceed 5 h [63]. This is considered an advantage because exposure to relatively unstable and short-lived ‘reactive species’ occurs in the exponential phase of bacterial growth, thereby promoting mutation fixation of DNA damage. Theoretically, this is relevant because the half-life of α-hydroxylation metabolites (i.e. the first step in CYP2E1 metabolic activation) is exceedingly short, lasting only minutes in solutions with a pH of 6 or less and no more than ~10 seconds at physiological pH [64]. Moreover, the half-life of diazonium ions, the ultimate mutagenic species, is even shorter.

BMD-modelling of NDMA and NDEA mutagenic potencies does not support this view, since both pre-incubation and plate incorporation Ames test methods resulted in top-quartile rankings, suggesting both Ames test methods have comparable sensitivities since they were among the assay conditions associated with the highest mutagenic potencies. With NDEA, the pre-incubation method was more sensitive in terms of mutagenic potency and in combination with hamster liver S9, accounted for the top four rankings in the BMD-modelling. Positive Ames test results were also observed with rat liver S9 activation (in plate- and pre-incubation methods), however, overall BMD mutagenic potencies were generally inferior. As expected, in the absence of liver S9-activation, neither NDMA nor NMEA were mutagenic in the Ames test. NDEA is also non-mutagenic in the absence of metabolic activation [60], although such conditions were not tested in the current study. Our findings are consistent with those reported for other alkyl NAs [60], and those reported in a wider analysis of OECD 471 compliant studies conducted with an extensive range of NA structures [27].

The work reported here consistently identified *Salmonella typhimurium* strains TA1535 and TA100 and *Escherichia coli* strain WP2uvrA (pKM101) as the most sensitive bacterial tester strains for NDMA, NDEA, and NMEA-induced mutagenicity. The sensitivity of these three bacterial strains for NAs has been widely reported in the literature [26,60,65]. This is not surprising, given the primary mechanism of mutagenicity is DNA alkylation, which can result in DNA replication errors if not repaired, and in turn, cause base-pair substitution mutations [47,66]. These bacterial strains have been engineered to detect base-pair substitutions and contain modifications to their DNA repair processes, which contribute to their sensitivity towards NAs. Importantly, *S. typhimurium* TA1535 & TA100 and *E. coli* WP2uvrA (pKM101) are among the combination of strains recommended in OECD 471 guideline [22] and therefore they should be included in the standard 5-strain testing strategy for testing NAs.

Vehicle choice is another important Ames test assay parameter, which can significantly impact performance in several ways. Some vehicles are toxic, particularly at higher concentrations, which can affect bacterial growth, and therefore the ability of certain chemicals to manifest mutagenicity. This can be an issue when using the pre-incubation method, since bacteria are exposed to a higher percentage of solvent vehicle, for longer periods with the test agent, compared with the plate incorporation assay. In our experience, certain vehicles such as tetrahydrofuran and acetonitrile exhibit higher toxicity in the pre-incubation assay (data not shown) and these vehicles are generally avoided with this method. Assay adaptations to minimize the potential for toxicity can include adjustments to vehicle volume—for example, the standard 100 µl volume (7.7% vehicle) used in the plate incorporation method can be reduced to 50 µl (3.7% vehicle) in the pre-incubation method. However, test compound solubility in a particular vehicle is also a key factor, some compounds are insoluble in aqueous solutions and necessitate the use of polar organic vehicles. Another layer, which adds further complexity to vehicle choice, is the need to obtain a minimum formulation to achieve the limit concentration (5 mg/plate) mandated by OECD TG-471 [22]. These considerations may necessitate a trade-off, both for vehicle selection and the final volume added to the test system, and this is often a case-by-case decision.

A further concern is that certain organic solvent vehicles can be substrates for cytochrome P450 enzymes, or they may inhibit cytochrome P450-dependent mixed-function oxidase activity. For example, organic solvents such as methanol, acetonitrile, dimethyl sulfoxide (DMSO), acetone, and ethanol have been shown to inhibit CYP1A, CYP2C, CYP2D, CYP2E, and CYP3A-mediated metabolism in a concentration-dependent manner in non-induced rat liver microsomes [67]. Therefore, there is a concern that some solvent vehicles can reduce and/or inhibit the metabolic-activation of pro-mutagens in the Ames test. CYP2E1 catalyzed metabolism is a necessary and critical step in the metabolic activation of alkyl-NAs, via α-hydroxylation [68], and ultimately results in the formation of the reactive diazonium ion responsible for DNA alkylation [69]. Our data show that various solvent vehicles impact the rates of conversion of the CYP2E1 substrate, chlorzoxazone to 6-hydroxychlorzoxazone, in both rat and hamster-induced liver S9. The amount of inhibition was vehicle-dependent, with DMSO among the most potent inhibitors of chlorzoxazone metabolism. DMSO has previously been shown to inhibit rat CYP2E1 metabolism at low percentage volume concentrations in non-induced liver microsomes [67], and can diminish the mutagenicity of pro-mutagens in the Ames test [29], including *N*-nitrosodialkylamines [23,30]. Therefore, there have been specific regulatory concerns about the use of DMSO as a vehicle for the assessment of NAs in the Ames test.

The mutagenicity of NDMA and NDEA (dissolved in DMSO) was readily detected in both the plate incorporation and pre-incubation versions of the Ames test using hamster liver S9 activation (in strains TA100, TA1535, and WP2uvrA (pKM101)). With NDEA, there appeared to be little qualitative differences when DMSO, methanol, or water was used as the vehicle, although responses with NDMA in DMSO were less potent compared with water or methanol. This data supports the findings from Trejo-Martin *et al*. [27] which state a non-DMSO solvent as preferable for certain small-alkyl NAs. Positive Ames test results were also observed using other vehicles, that is, with acetonitrile, acetone, and DMF for NDMA; and acetonitrile, acetone, DMF, and NMP for NDEA, respectively, although again these tended to be far less potent compared with water or methanol. In contrast, the use of induced rat-liver S9 was clearly inferior for NDMA or NDEA activation, irrespective of the vehicle used. These results suggest the overall mutagenic response is less dependent on the vehicle, per se, rather than the species of liver metabolic activation used. Furthermore, at face value anyway, the vehicle-specific mutagenic potencies do not appear to correlate with their corresponding vehicle-induced CYP2E1 enzyme inhibition, as determined by chlorzoxazone conversion to 6-hydroxychlorzoxazone.

This suggests concerns about the potential inhibition of metabolic activation by organic solvent vehicles and their impact on Ames test performance may be overstated. The adaptations inherent to the assay, that is, (i) the use of cytochrome P450 induced livers shifting the balance in favour of metabolic activation; (ii) bacterial genetic traits disposed to increased chemical exposure and mutagenesis; and (iii) mandated high test concentrations (up to 5000 μg/plate) with options for pre-incubation (and co-exposure of bacteria and test chemical within the milieu of the metabolic system), all contribute to increasing Ames test sensitivity, and likely off-set the potential for detrimental solvent vehicle mediated enzyme inhibition. As such, the sensitivity of the Ames test is heavily biased to elicit a positive response if sufficient DNA reactive metabolites are generated, and moreover, this is clearly influenced by the species of liver metabolism used. Given these considerations, and based on the results of the current study, in our view, water or methanol should be considered an appropriate vehicle for the assessment of alkyl-NAs, although, if aqueous solubility is a limiting factor, then more polar organic solvent vehicles can be considered, and this can include DMSO.

## Conclusions

The current study confirms the mutagenicity of alkyl-nitrosamines can be readily detected in the bacterial reverse mutation test (Ames test). Using advanced data analytics and modelling we show the assay parameters most sensitive for this class of compound including the pre-incubation method (with a 30-min incubation period) compared with plate-incorporation. Either water or methanol was suitable as vehicles, and hamster-induced liver S9 was the superior exogenous metabolic system, although rat liver S9 also elicited a positive response, albeit with less potency. Since there are examples of nitrosamines which are Ames test positive with rat liver S9 and negative with hamster liver S9 [27], we recommend both systems be used in parallel at the present time. *S. typhimurium* TA1535 and TA100 and *E. coli* WP2uvrA (pKM101), were the most sensitive bacterial strains, and these strains should be included as part of the 5-strain Ames test when evaluating the mutagenicity of nitrosamines. To further evaluate the performance of the Ames test under these assay parameters, we have tested a range of discordant NAs, of varying chemical structure, and the results of this work will be the subject of a follow-on publication.

## Supplementary Material

gead033_suppl_Supplementary_Tables_S1-S4_Figures_S1-S16

gead033_suppl_Supplementary_Tables_S5

## Data Availability

The data underlying this article are available in the article and in its online supplementary material.
