# Supplementary material for: Ames test study designs for nitrosamine mutagenicity testing: qualitative and quantitative analysis of key assay parameters
Source: Mutagenesis. 2023 Dec 19;39(2):78–95. doi: 10.1093/mutage/gead033 (PMC10928841; doi:10.1093/mutage/gead033)
Supplement: gead033_suppl_Supplementary_Tables_S1-S4_Figures_S1-S16 [file gead033_suppl_supplementary_tables_s1-s4_figures_s1-s16.pdf]

Ames Test study designs for nitrosamine mutagenicity testing: qualitative and quantitative analysis of key assay parameters – Supplementary Tables

Authors:

Dean N. Thomas, John W. Wills, Helen Tracey, Sandy J. Baldwin, Mark Burman, Abbie N. Williams, Dannii S.G. Harte, Ruby A. Buckley and Anthony M. Lynch

Constituent	Volume/Weight	Final Concentration mM
**NADP**	31.5 mg	4
**Glucose-6-phosphate**	50 µL	5
**Salt Solution**	0.2 mL	
Magnesium chloride solution (0.4M)		8
Potassium chloride solution (1.65M)		33
**0.2M Phosphate buffer**	5.0 mL	100
**Sterile Water**	3.75 mL	Not applicable
**S9 Fraction (Rat or Hamster)**	1.0 mL	Not applicable

Table 1 (Suppl.). Summary of S9 composition (with a NADPH generating system) per 10 mL of S9-mix, the final S9 mix contained 10% (v/v) of S9-liver fraction.

Strain	Origin	Genotype	Type of reverse mutation
**TA100**	ECACC (NCTC 12116)	his G 46, uvrB−, rfa−, + R-factor (pKM101)	Base pair substitution
**TA1535**	ECACC (NCTC 12117)	his G 46, uvrB−, rfa−	Base pair substitution
**TA1537**	ECACC (NCTC 12118)	his C 3076, uvrB−, rfa	Frameshift mutation
**TA98**	ECACC (NCTC 12115)	his D 3052, uvrB−, rfa−, + R-factor (pKM101)	Frameshift mutation
**WP2uvrA (pKM101)**	National Collection of Type Cultures (NCIMB 11703)	trp−, uvrA, + R-factor (pKM101)	Base pair substitution ans small deletions

Table 2 (Suppl.). Origin and Genotype of Bacterial Strains

	Plate Incorporation	Pre-Incubation
**Vehicle/Test article**	100 µL	50 µL
**Vehicle percentage**	7.7%	3.7%
**Positive control**	100 µL	100 µL
**Bacterial Strain**	100 µL	100 µL
**S9-mix/Buffer**	500 µL	500 µL

Table 3 (Suppl.). Summary of Incubation constituents

Strain	In the presence of liver S9-mix	Concentration (vehicle)	In the absence of liver S9-mix	Concentration (vehicle)
**TA100**	2-Aminoanthracene (2-AAN)	5 µg/plate (DMSO)	Sodium Azide (NaN3)	2 µg/plate (water)
**TA1535**	2-Aminoanthracene (2-AAN)	5 µg/plate (DMSO)	Sodium Azide (NaN3)	2 µg/plate (water)
**TA1537**	2-Aminoanthracene (2-AAN)	5 µg/plate (DMSO)	9-Amino Acridine (9-AAC)	1 µg/plate (DMSO)
**TA98**	Benzo[a]pyrene (BAP)	10 µg/plate (DMSO)	2-Nitrofluorene (2NF)	1 µg/plate (DMSO)
**WP2uvrA (pKM101)**	2-Aminoanthracene (2-AAN)	10 µg/plate (DMSO)	4-Nitroquinoline-1-oxide (4NQO)	2 µg/plate (DMSO)

Table 4 (Suppl.). Positive controls per bacterial strain

Ames Test study designs for nitrosamine mutagenicity testing: qualitative and quantitative analysis of key assay parameters – Supplementary Data

NDMA and NDEA plate incorporation mean ratio treated/vehicle control data.

Authors:

Dean N. Thomas, John W. Wills, Helen Tracey, Sandy J. Baldwin, Mark Burman, Abbie N. Williams, Dannii S.G. Harte, Ruby A. Buckley and Anthony M. Lynch

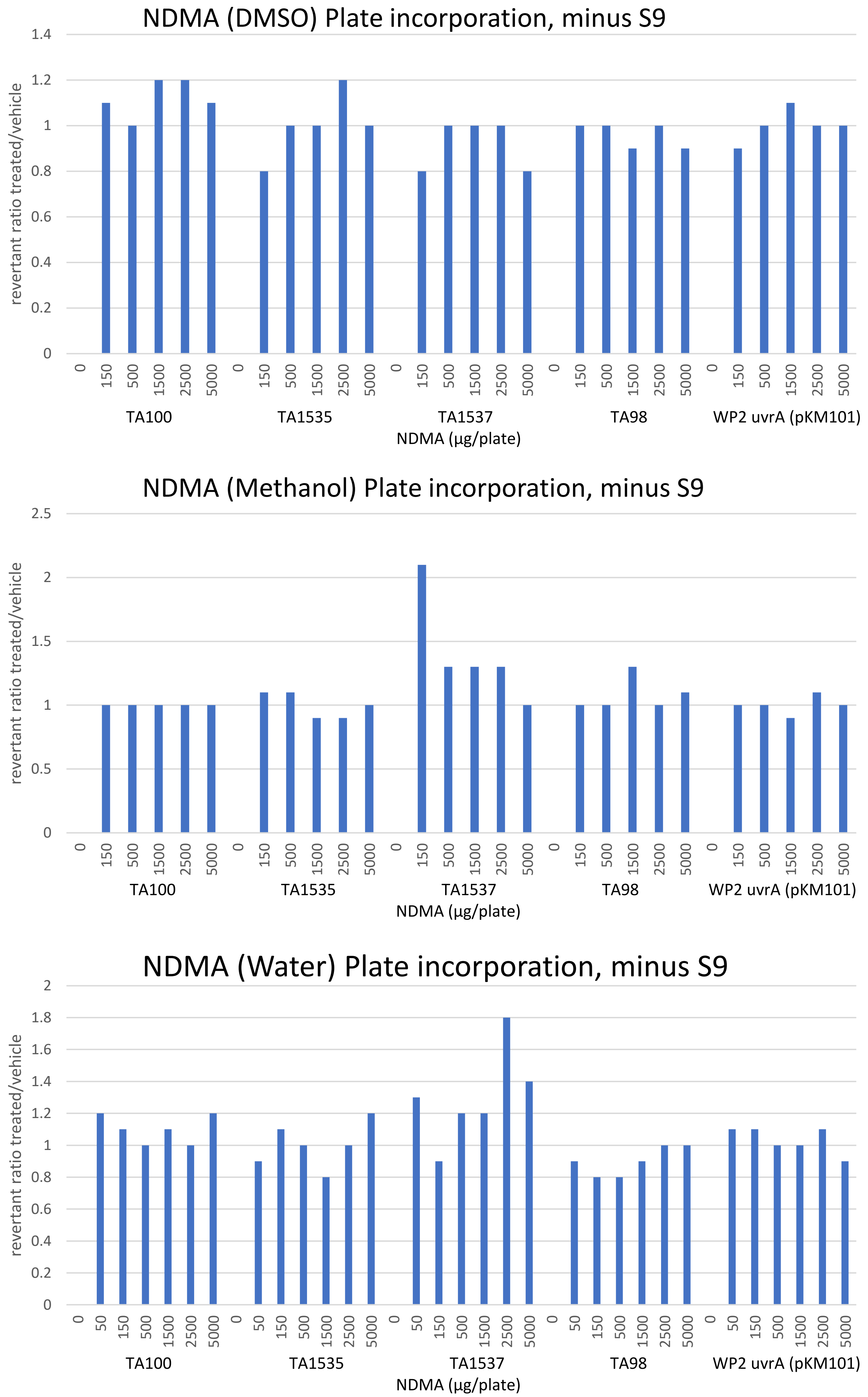

Figure 1 (Suppl.) Bacterial reverse mutation plate incorporation mean revertant ratio treated/vehicle control data with NDMA, using solvent vehicles DMSO (top), methanol (middle) and purified water (bottom), in the absence of S9-mix (Y axis representative of mean revertant ratio treated/vehicle, X axis representative of test article concentration (µg/plate) per bacterial strain). Blue bars refer to concentrations where mean revertant ratio treated/vehicle is lower than 2-fold for TA100, TA98 and WP2uvrA (pKM101) and 3-fold for TA1535 and TA1537. The maximum concentration tested was 5000 ug/plate, the maximum concentration in accordance with current guidelines.

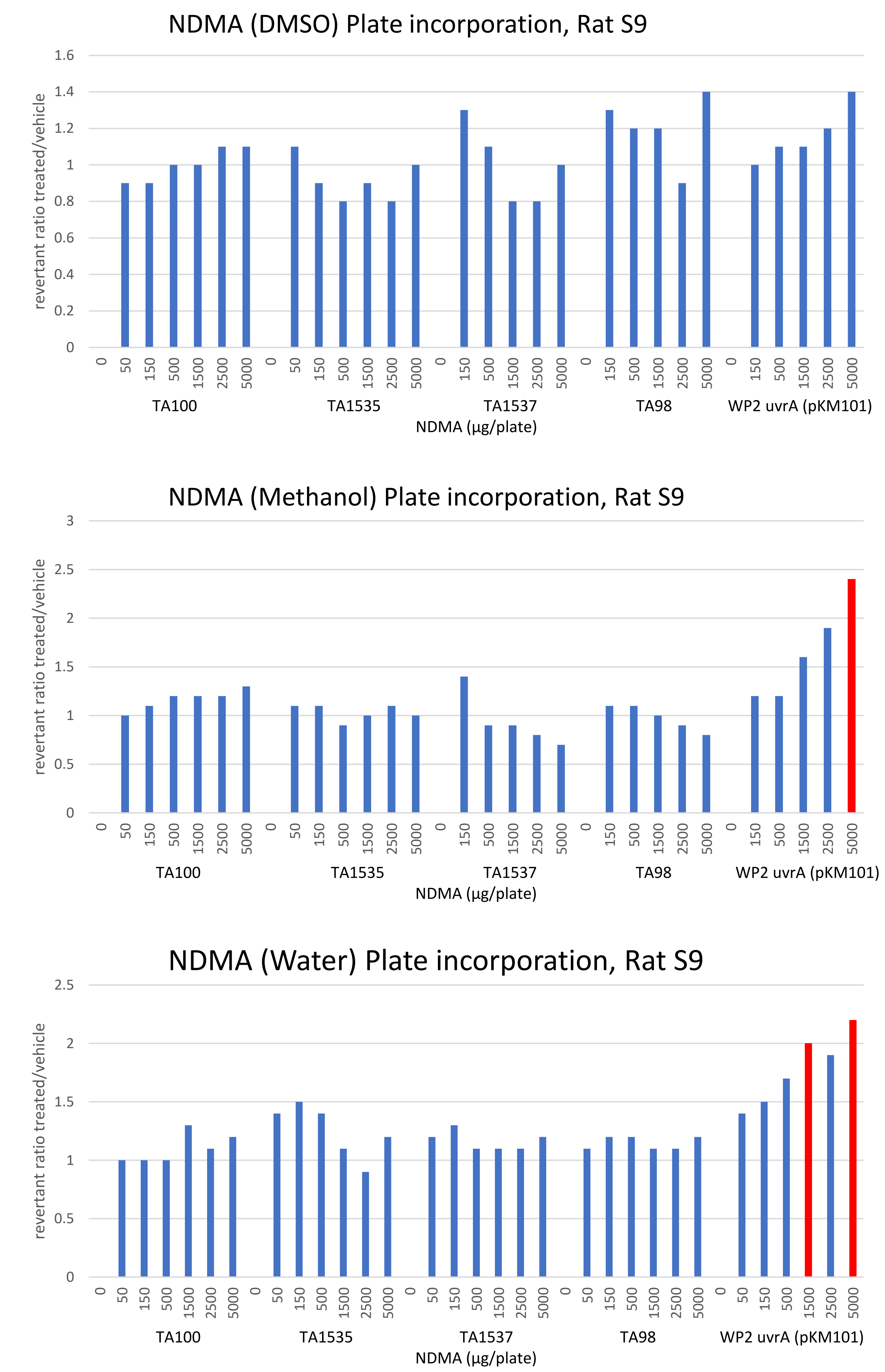

Figure 2 (Suppl.) Bacterial reverse mutation plate incorporation mean revertant ratio treated/vehicle control data with NDMA, using solvent vehicles DMSO (top), methanol (middle) and purified water (bottom), in the presence of Rat liver S9-mix (Y axis representative of mean revertant ratio treated/vehicle, X axis representative of test article concentration (µg/plate) per bacterial strain). Bars represent concentrations where mean revertant ratio treated/vehicle is less than (blue) or exceed (red) the 2-fold for TA100, TA98 and WP2uvrA (pKM101) and 3-fold for TA1535 and TA1537. The maximum concentration tested was 5000 ug/plate, the maximum concentration in accordance with current guidelines.

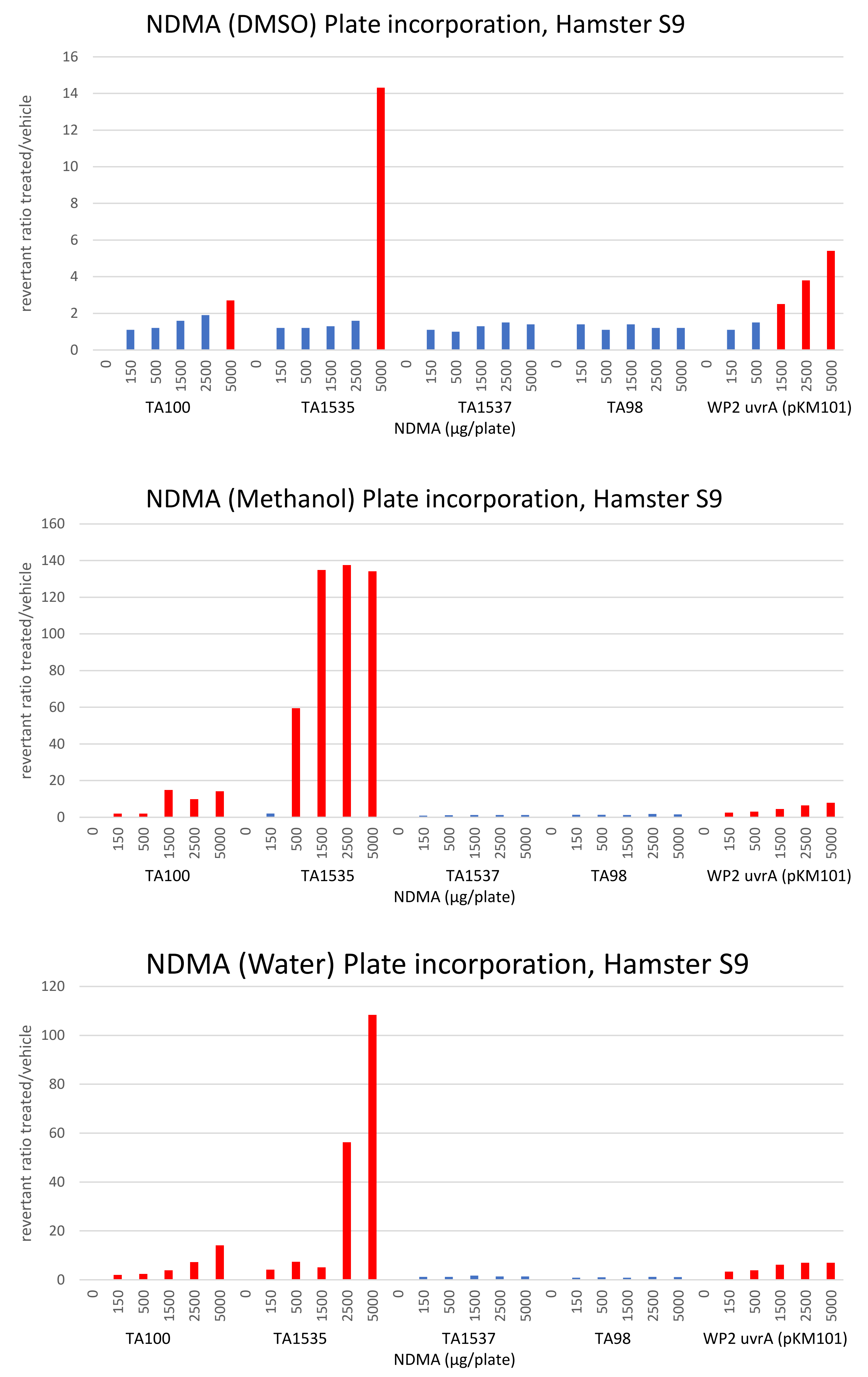

Figure 3 (Suppl.) Bacterial reverse mutation plate incorporation mean revertant ratio treated/vehicle control data with NDMA, using solvent vehicles DMSO (top), methanol (middle) and purified water (bottom), in the presence of Hamster liver S9-mix (Y axis representative of mean revertant ratio treated/vehicle, X axis representative of test article concentration (µg/plate) per bacterial strain). Bars represent concentrations where mean revertant ratio treated/vehicle is less than (blue) or exceed (red) the 2-fold for TA100, TA98 and WP2uvrA (pKM101) and 3-fold for TA1535 and TA1537. The maximum concentration tested was 5000 ug/plate, the maximum concentration in accordance with current guidelines.

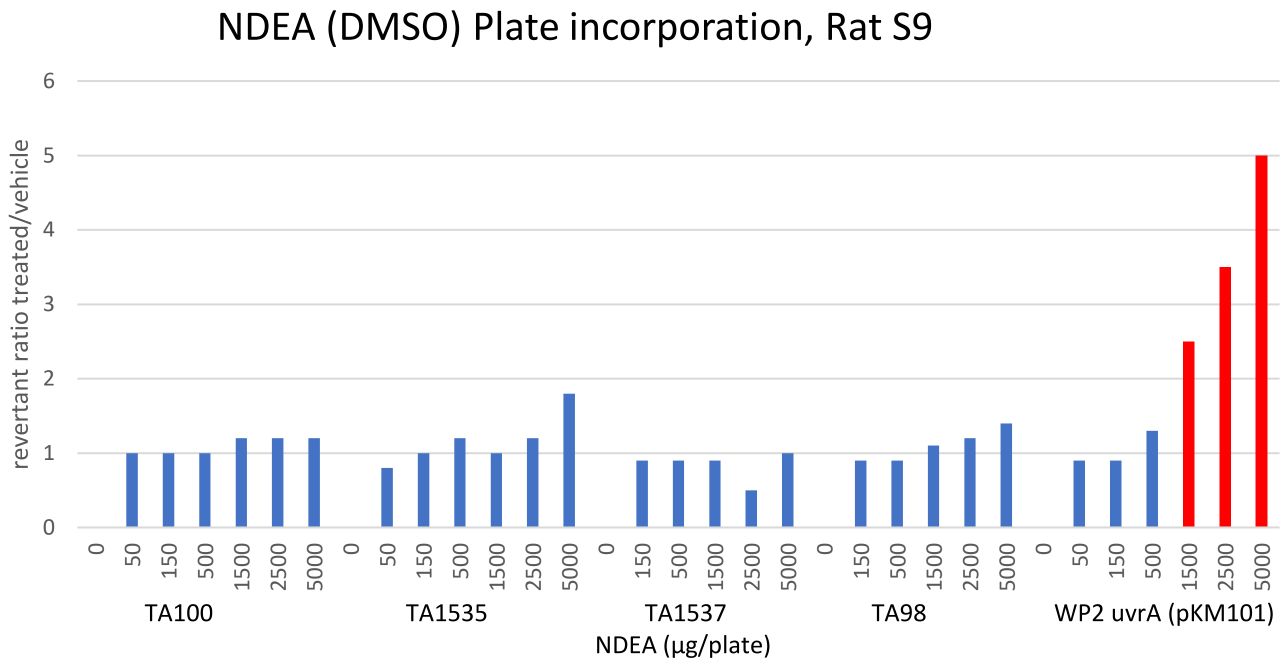

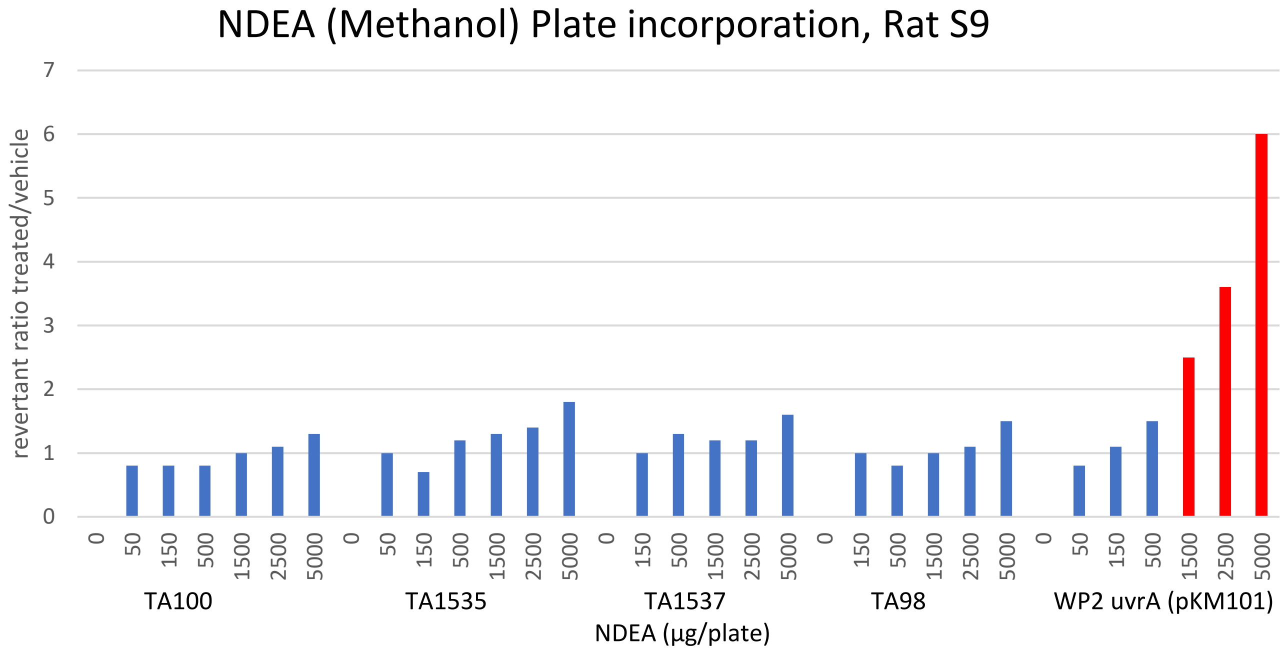

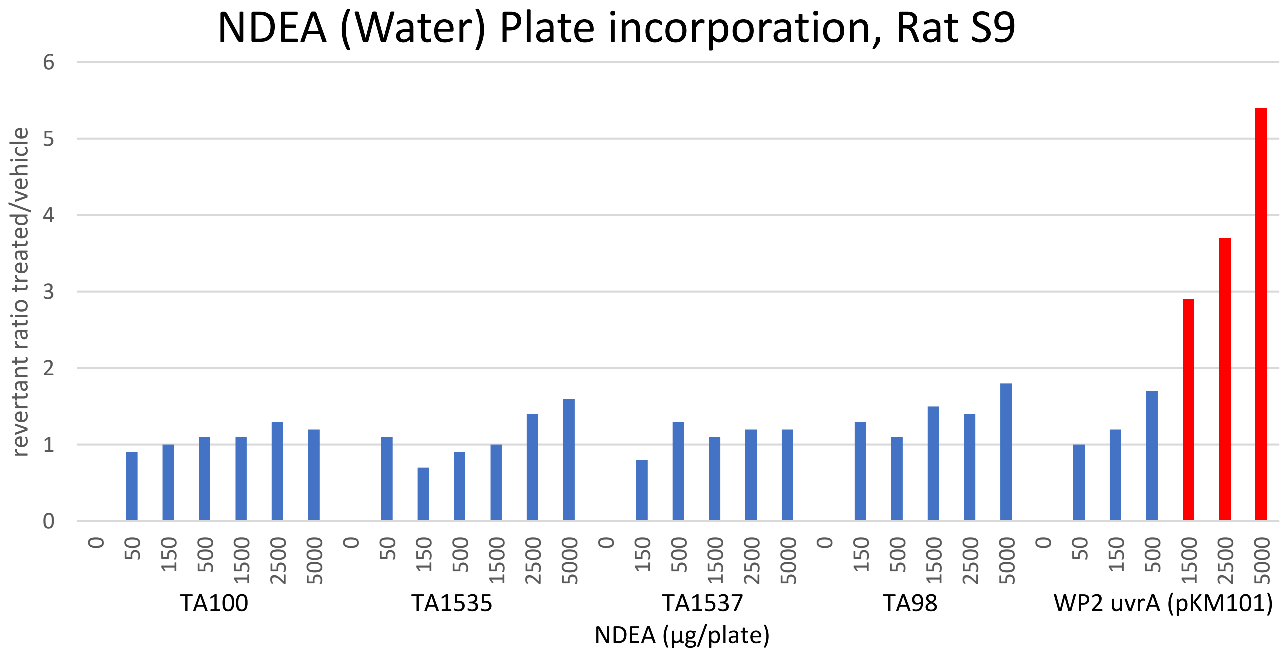

Figure 4 (Suppl.) Bacterial reverse mutation plate incorporation mean revertant ratio treated/vehicle control data with NDEA, using solvent vehicles DMSO (top), methanol (middle) and purified water (bottom), in the presence of Rat liver S9-mix (Y axis representative of mean revertant ratio treated/vehicle, X axis representative of test article concentration (µg/plate) per bacterial strain). Bars represent concentrations where mean revertant ratio treated/vehicle is less than (blue) or exceed (red) the 2-fold for TA100, TA98 and WP2uvrA (pKM101) and 3-fold for TA1535 and TA1537. The maximum concentration tested was 5000 ug/plate, the maximum concentration in accordance with current guidelines.

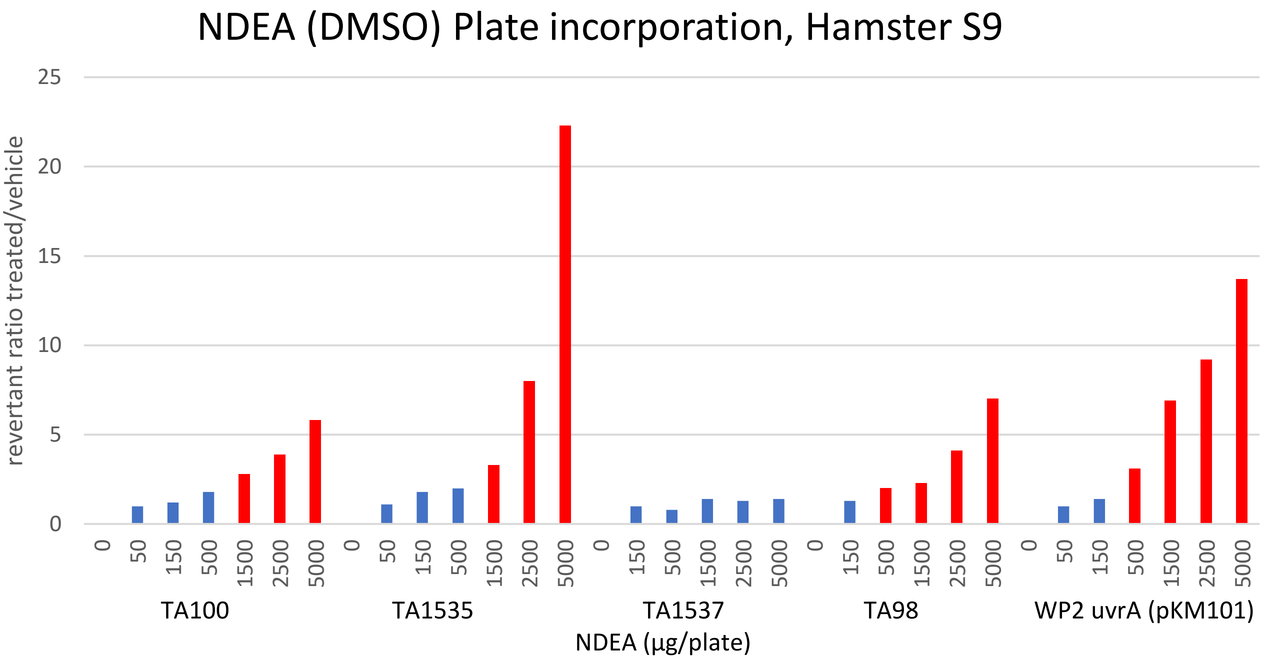

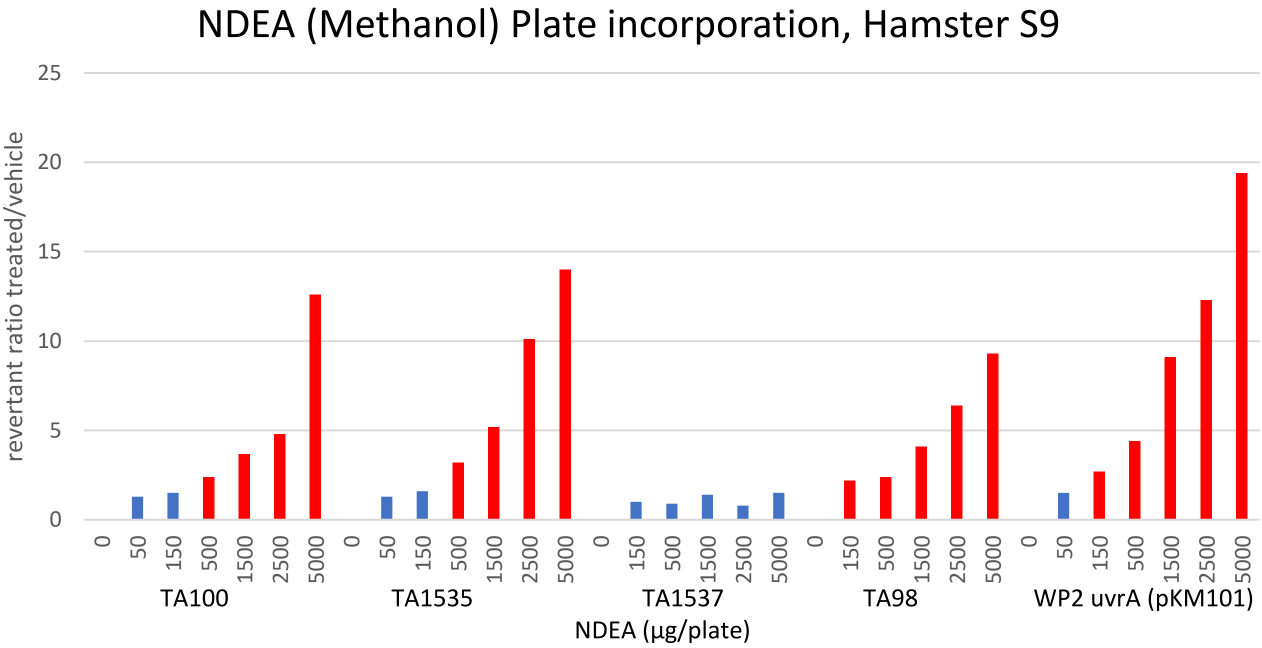

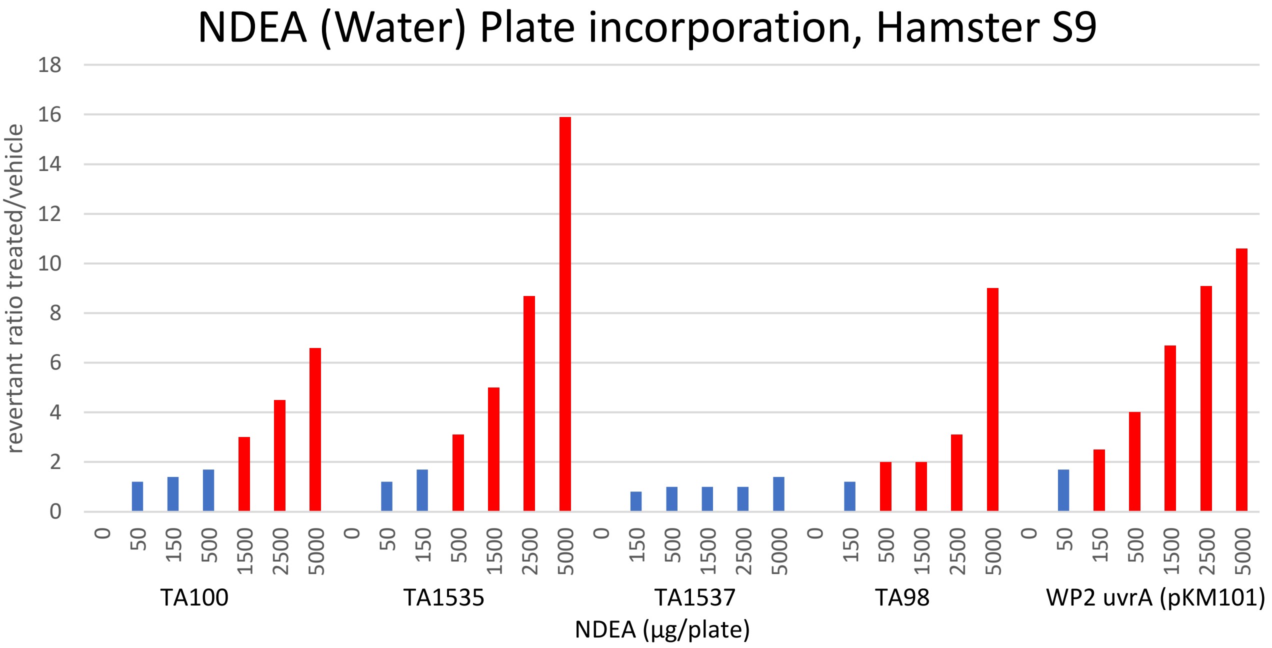

Figure 5 (Suppl.) Bacterial reverse mutation plate incorporation mean revertant ratio treated/vehicle control data with NDEA, using solvent vehicles DMSO (top), methanol (middle) and purified water (bottom), in the presence of Hamster liver S9-mix (Y axis representative of mean revertant ratio treated/vehicle, X axis representative of test article concentration (µg/plate) per bacterial strain). Bars represent concentrations where mean revertant ratio treated/vehicle is less than (blue) or exceed (red) the 2-fold for TA100, TA98 and WP2uvrA (pKM101) and 3-fold for TA1535 and TA1537. The maximum concentration tested was 5000 ug/plate, the maximum concentration in accordance with current guidelines.

Ames Test study designs for nitrosamine mutagenicity testing: qualitative and quantitative analysis of key assay parameters – Supplementary Data

NDMA pre-incubation mean ratio treated/vehicle control data.

Authors:

Dean N. Thomas, John W. Wills, Helen Tracey, Sandy J. Baldwin, Mark Burman, Abbie N. Williams, Dannii S.G. Harte, Ruby A. Buckley and Anthony M. Lynch

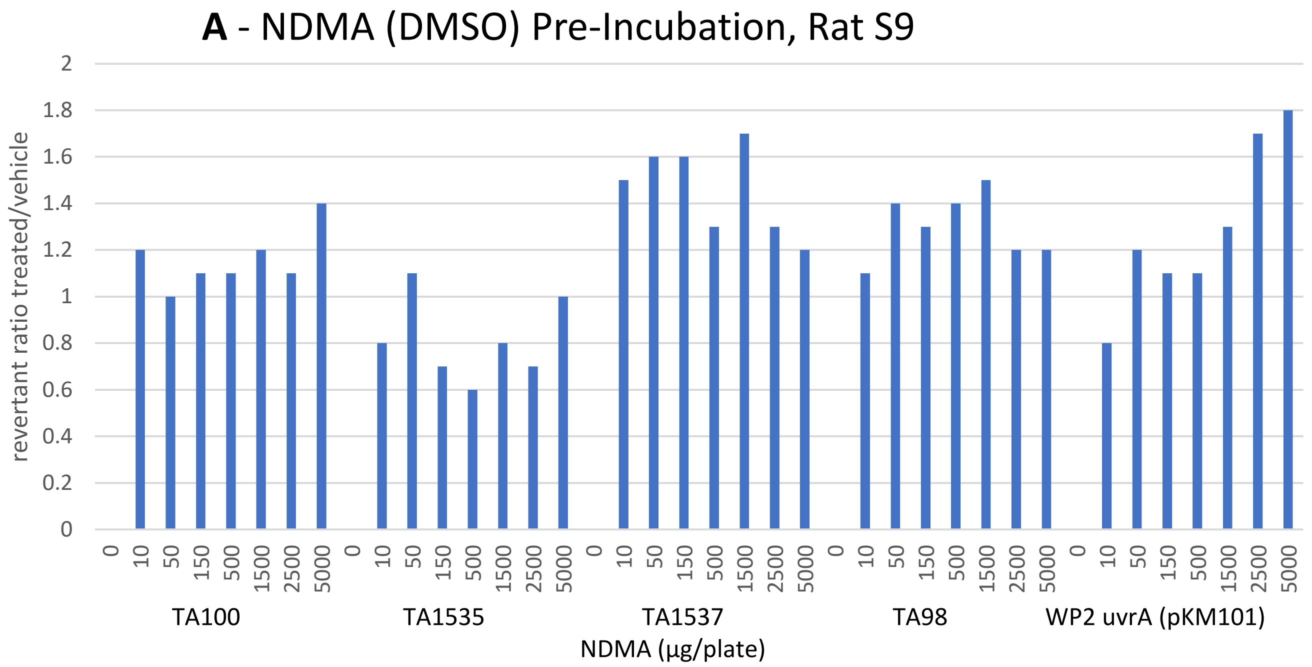
A - NDMA (DMSO) Pre-Incubation, Rat S9
revertant ratio treated/vehicle
0
0.2
0.4
0.6
0.8
1
1.2
1.4
1.6
1.8
2
0 10 50 150 500 1500 2500 5000
TA100
TA1535
TA1537
TA98
WP2 uvrA (pKM101)
NDMA (µg/plate)

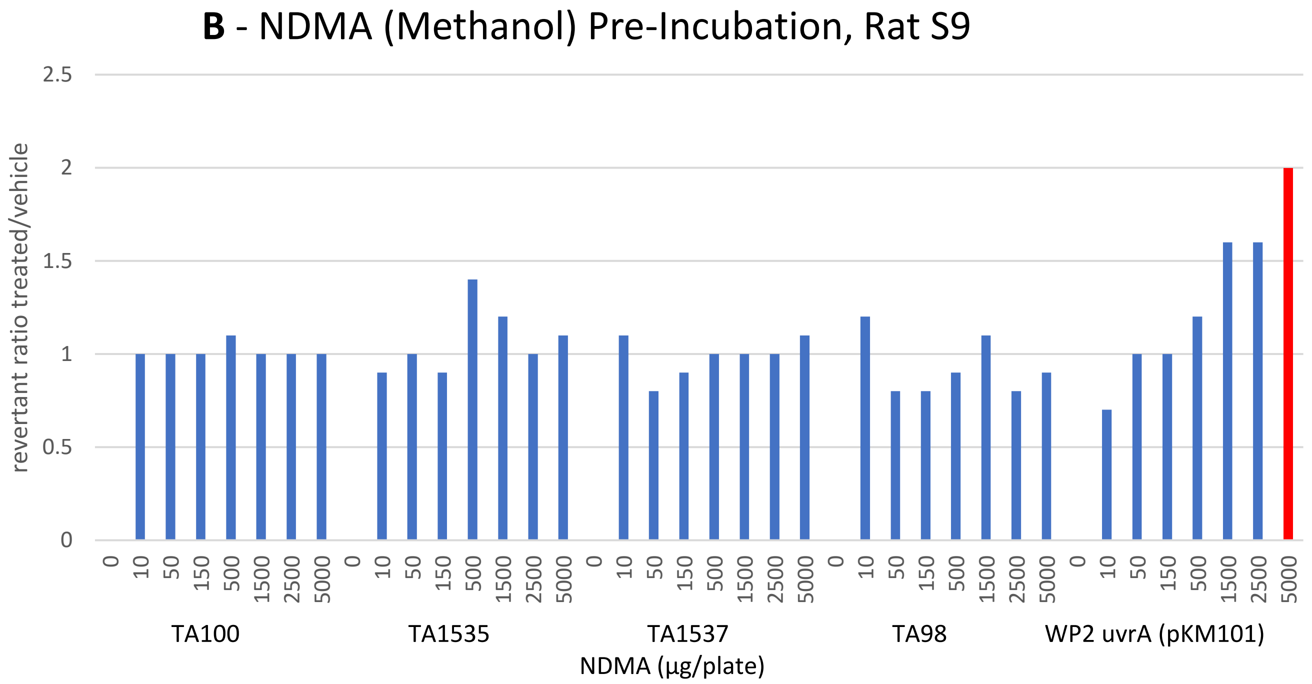
B - NDMA (Methanol) Pre-Incubation, Rat S9
revertant ratio treated/vehicle
0
0.5
1
1.5
2
2.5
0 10 50 150 500 1500 2500 5000
TA100
TA1535
TA1537
TA98
WP2 uvrA (pKM101)
NDMA (µg/plate)

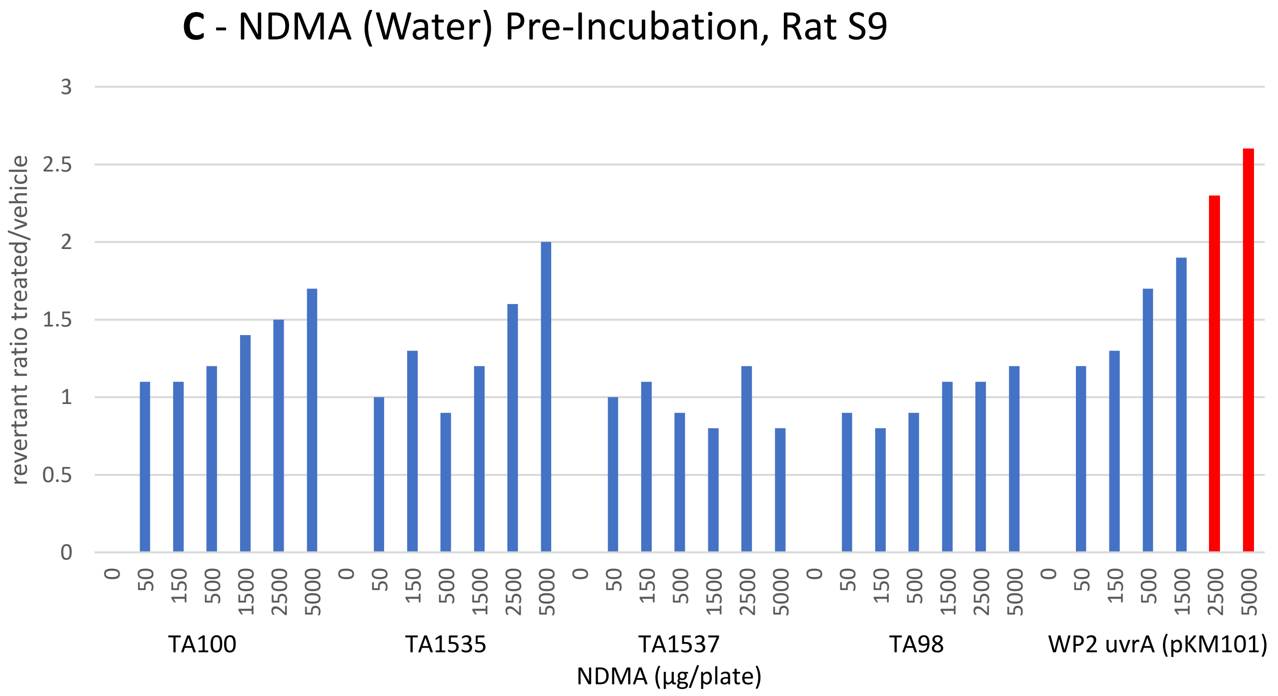
C - NDMA (Water) Pre-Incubation, Rat S9
revertant ratio treated/vehicle
0
0.5
1
1.5
2
2.5
3
0 50 150 500 1500 2500 5000
TA100
TA1535
TA1537
TA98
WP2 uvrA (pKM101)
NDMA (µg/plate)

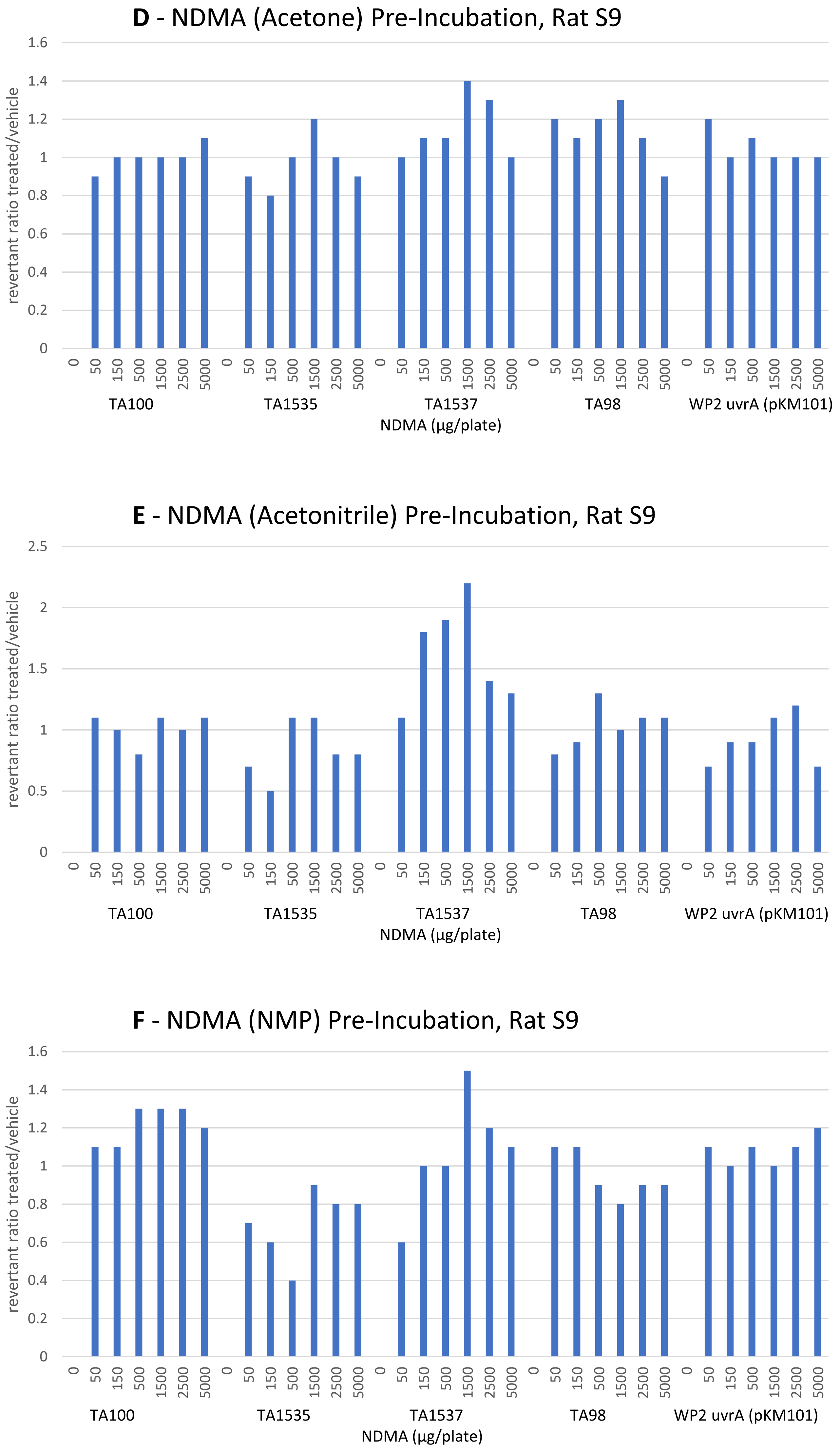
D - NDMA (Acetone) Pre-Incubation, Rat S9
revertant ratio treated/vehicle
TA100
TA1535
TA1537
TA98
WP2 uvrA (pKM101)
NDMA (µg/plate)
E - NDMA (Acetonitrile) Pre-Incubation, Rat S9
revertant ratio treated/vehicle
TA100
TA1535
TA1537
TA98
WP2 uvrA (pKM101)
NDMA (µg/plate)
F - NDMA (NMP) Pre-Incubation, Rat S9
revertant ratio treated/vehicle
TA100
TA1535
TA1537
TA98
WP2 uvrA (pKM101)
NDMA (µg/plate)

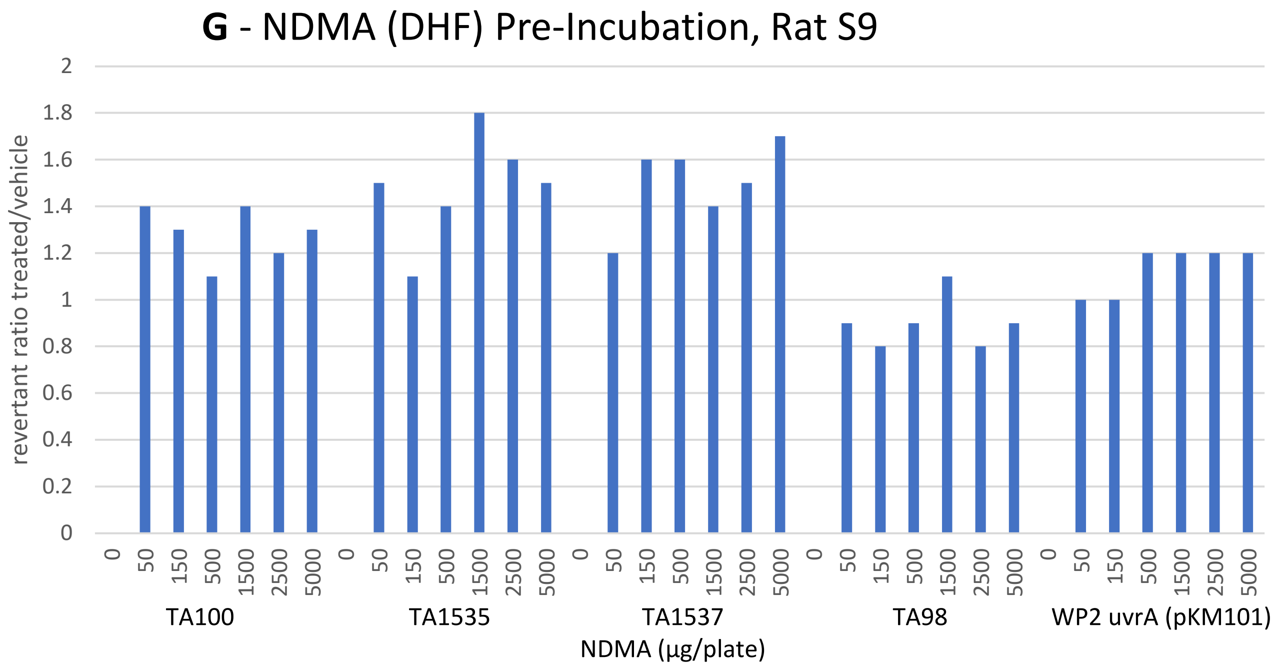

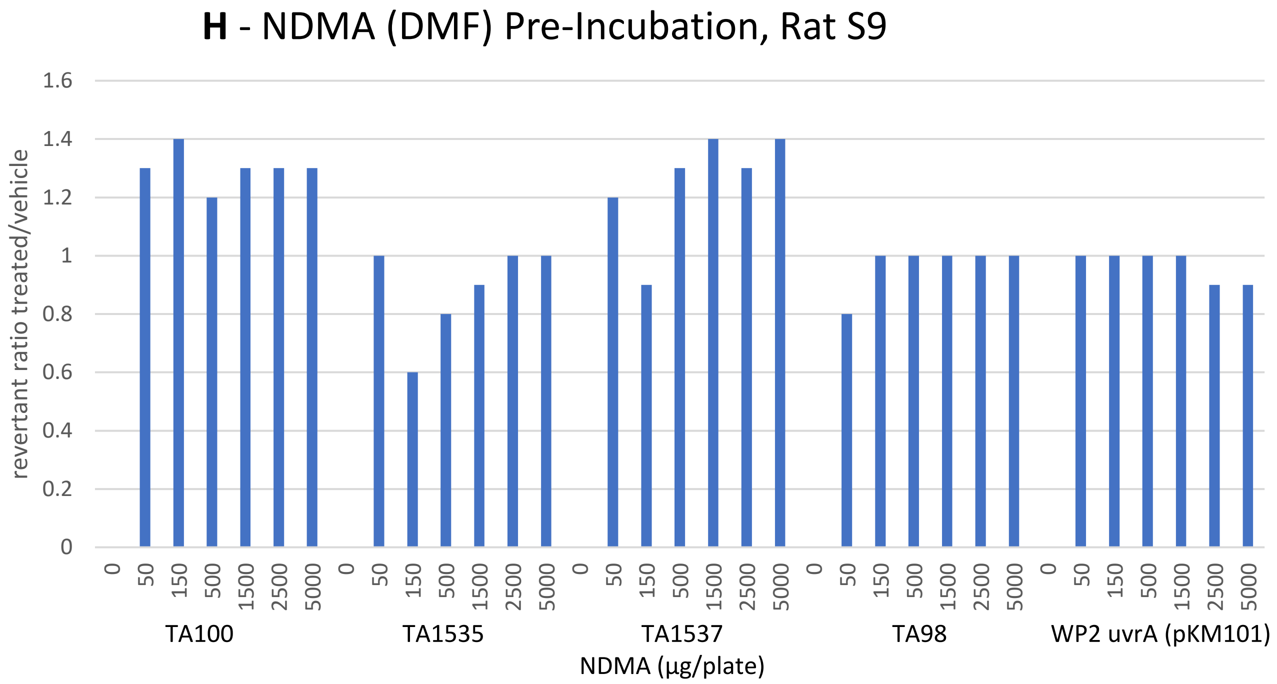

Figure 6 (Suppl.) Bacterial reverse mutation pre-incubation mean revertant ratio treated/vehicle control data with NDMA, using solvent vehicles DMSO (**A**), Methanol (**B**), Water (**C**), Acetone (**D**), Acetonitrile (**E**), NMP (**F**), DHF (**G**) and DMF (**H**), in the presence of Rat liver S9-mix (Y axis representative of mean revertant ratio treated/vehicle, X axis representative of test article concentration (µg/plate) per bacterial strain). Bars represent concentrations where mean revertant ratio treated/vehicle is less than (blue) or exceed (red) the 2-fold for TA100, TA98 and WP2uvrA (pKM101) and 3-fold for TA1535 and TA1537. The maximum concentration tested was 5000 ug/plate, the maximum concentration in accordance with current guidelines.

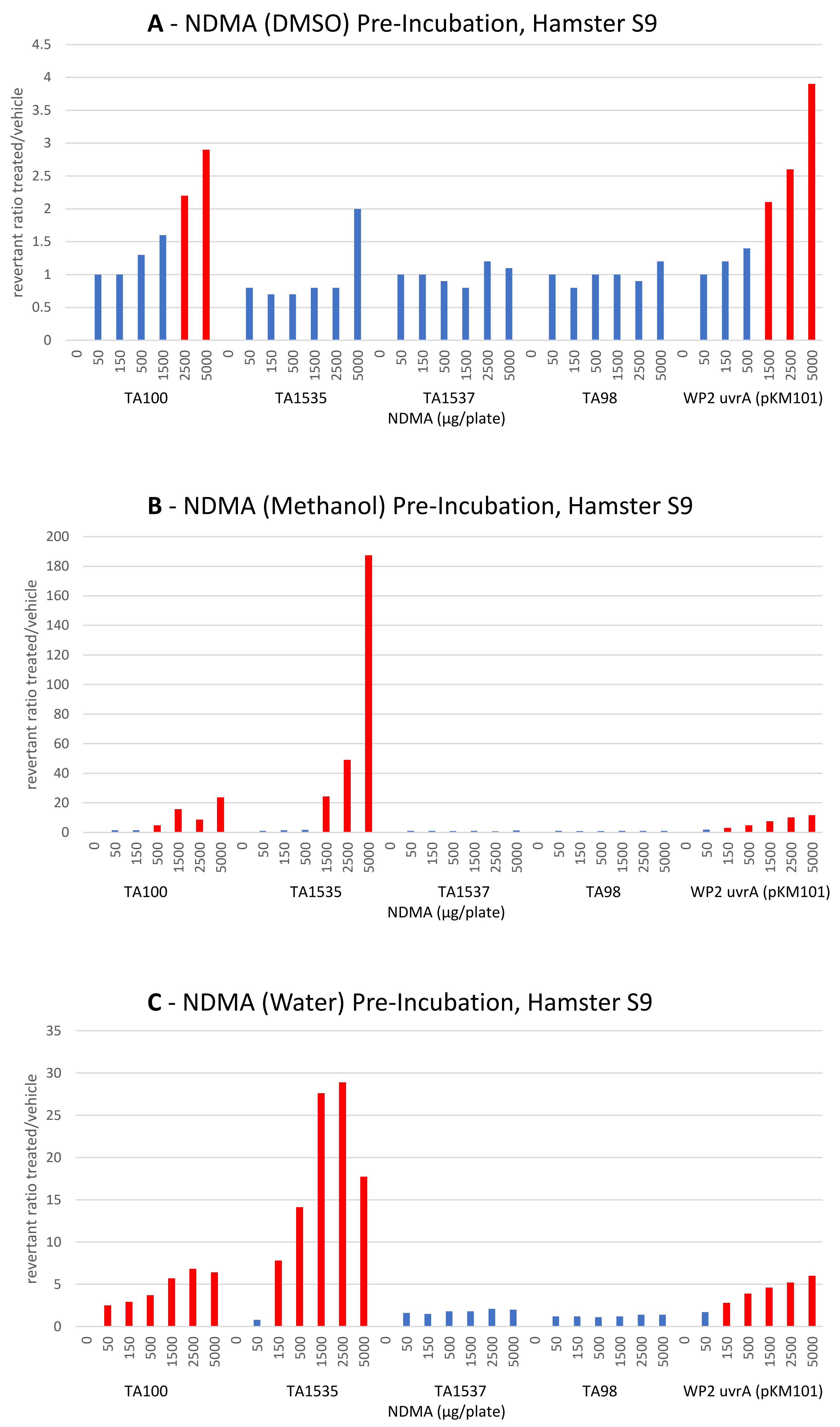
A - NDMA (DMSO) Pre-Incubation, Hamster S9
revertant ratio treated/vehicle
4.5
4
3.5
3
2.5
2
1.5
1
0.5
0
0 50 150 500 1500 2500 5000
TA100
TA1535
TA1537
TA98
WP2 uvrA (pKM101)
NDMA (µg/plate)
B - NDMA (Methanol) Pre-Incubation, Hamster S9
revertant ratio treated/vehicle
200
180
160
140
120
100
80
60
40
20
0
0 50 150 500 1500 2500 5000
TA100
TA1535
TA1537
TA98
WP2 uvrA (pKM101)
NDMA (µg/plate)
C - NDMA (Water) Pre-Incubation, Hamster S9
revertant ratio treated/vehicle
35
30
25
20
15
10
5
0
0 50 150 500 1500 2500 5000
TA100
TA1535
TA1537
TA98
WP2 uvrA (pKM101)
NDMA (µg/plate)

D - NDMA (Acetone) Pre-Incubation, Hamster S9

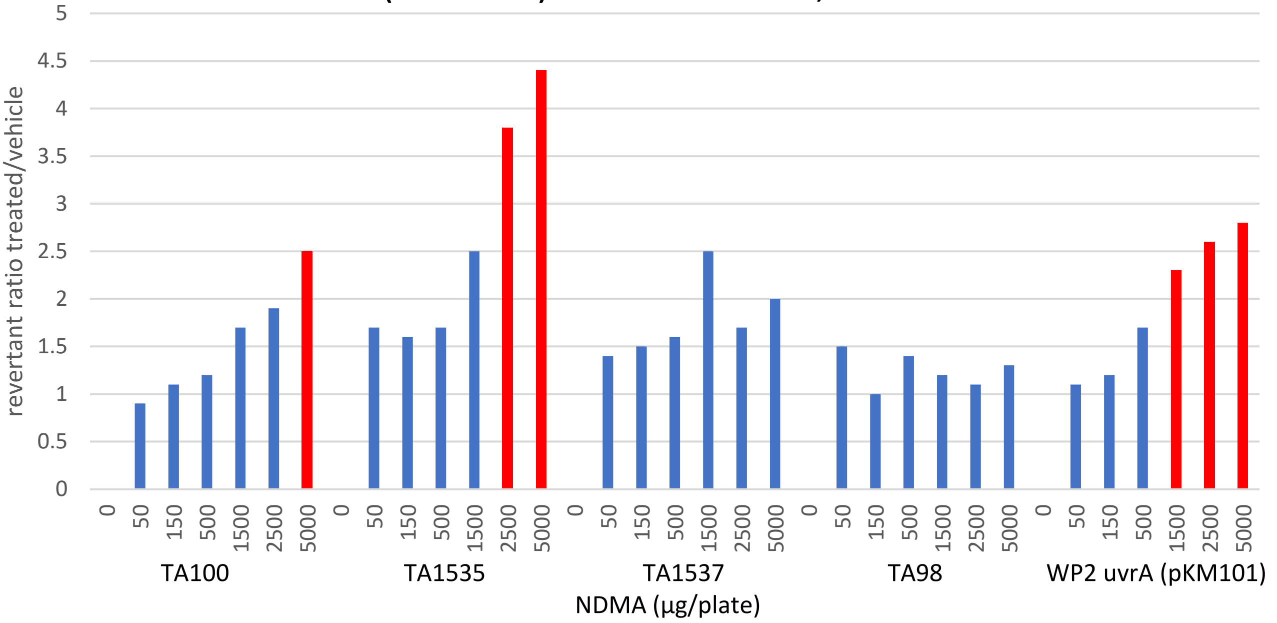

E - NDMA (Acetonitrile) Pre-Incubation, Hamster S9

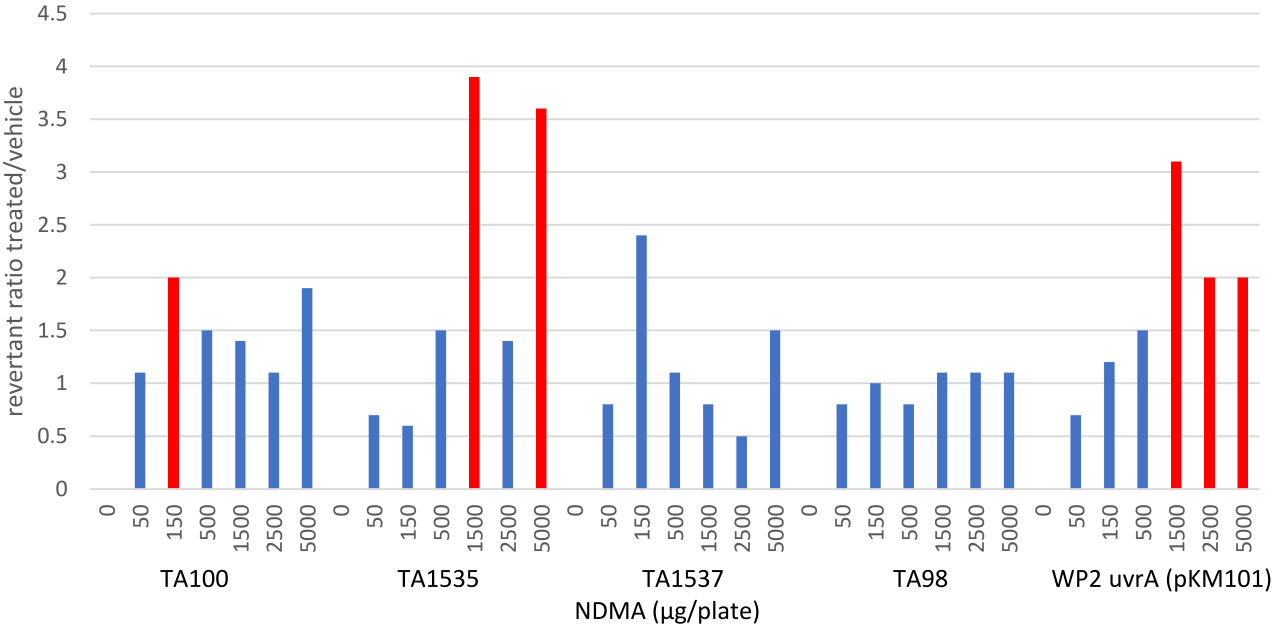

F - NDMA (NMP) Pre-Incubation, Hamster S9

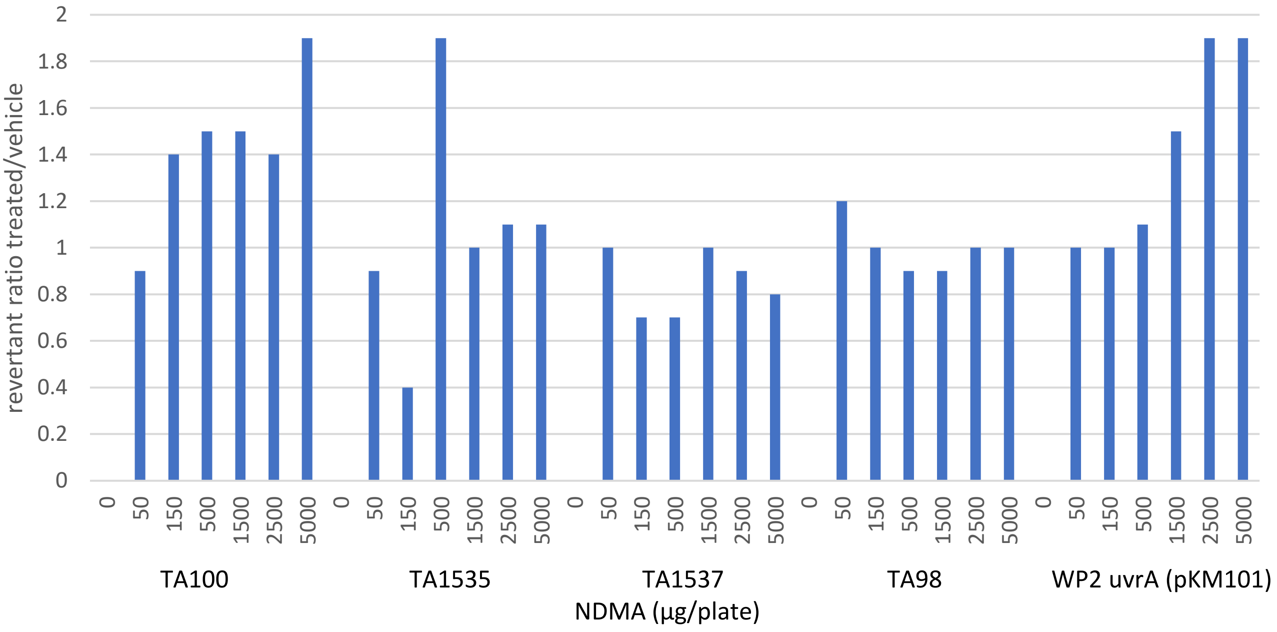

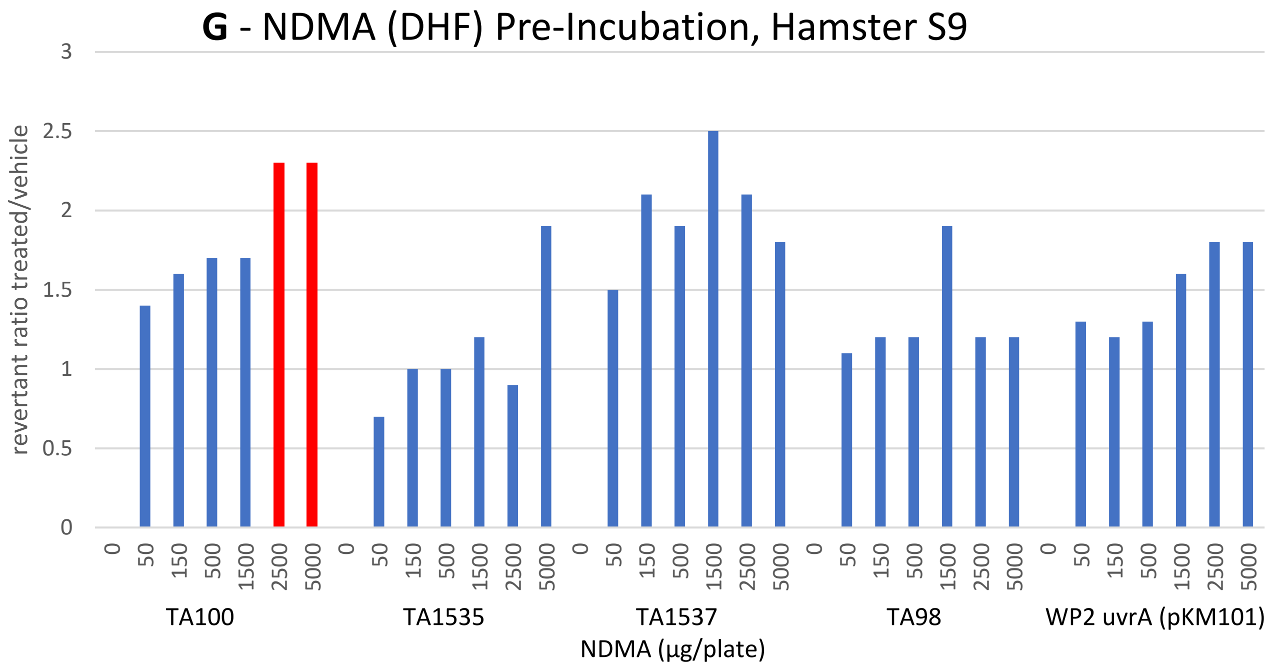

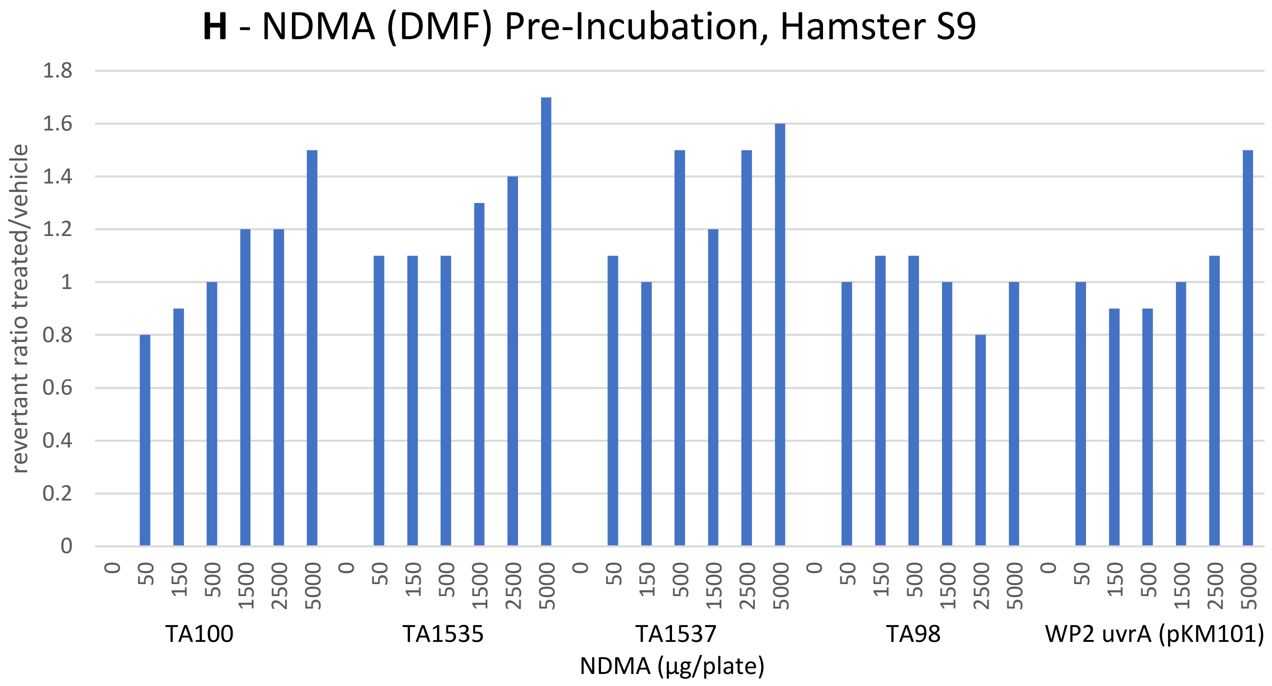

Figure 7 (Suppl.) Bacterial reverse mutation pre-incubation mean revertant ratio treated/vehicle control data with NDMA, using solvent vehicles DMSO (**A**), Methanol (**B**), Water (**C**), Acetone (**D**), Acetonitrile (**E**), NMP (**F**), DHF (**G**) and DMF (**H**), in the presence of Hamster liver S9-mix (Y axis representative of mean revertant ratio treated/vehicle, X axis representative of test article concentration (µg/plate) per bacterial strain). Bars represent concentrations where mean revertant ratios treated/vehicle is less than (blue) or exceed (red) the 2-fold for TA100, TA98 and WP2uvrA (pKM101) and 3-fold for TA1535 and TA1537. The maximum concentration tested was 5000 ug/plate, the maximum concentration in accordance with current guidelines.

Ames Test study designs for nitrosamine mutagenicity testing: qualitative and quantitative analysis of key assay parameters – Supplementary Data

NDEA pre-incubation mean ratio treated/vehicle control data.

Authors:

Dean N. Thomas, John W. Wills, Helen Tracey, Sandy J. Baldwin, Mark Burman, Abbie N. Williams, Dannii S.G. Harte, Ruby A. Buckley and Anthony M. Lynch

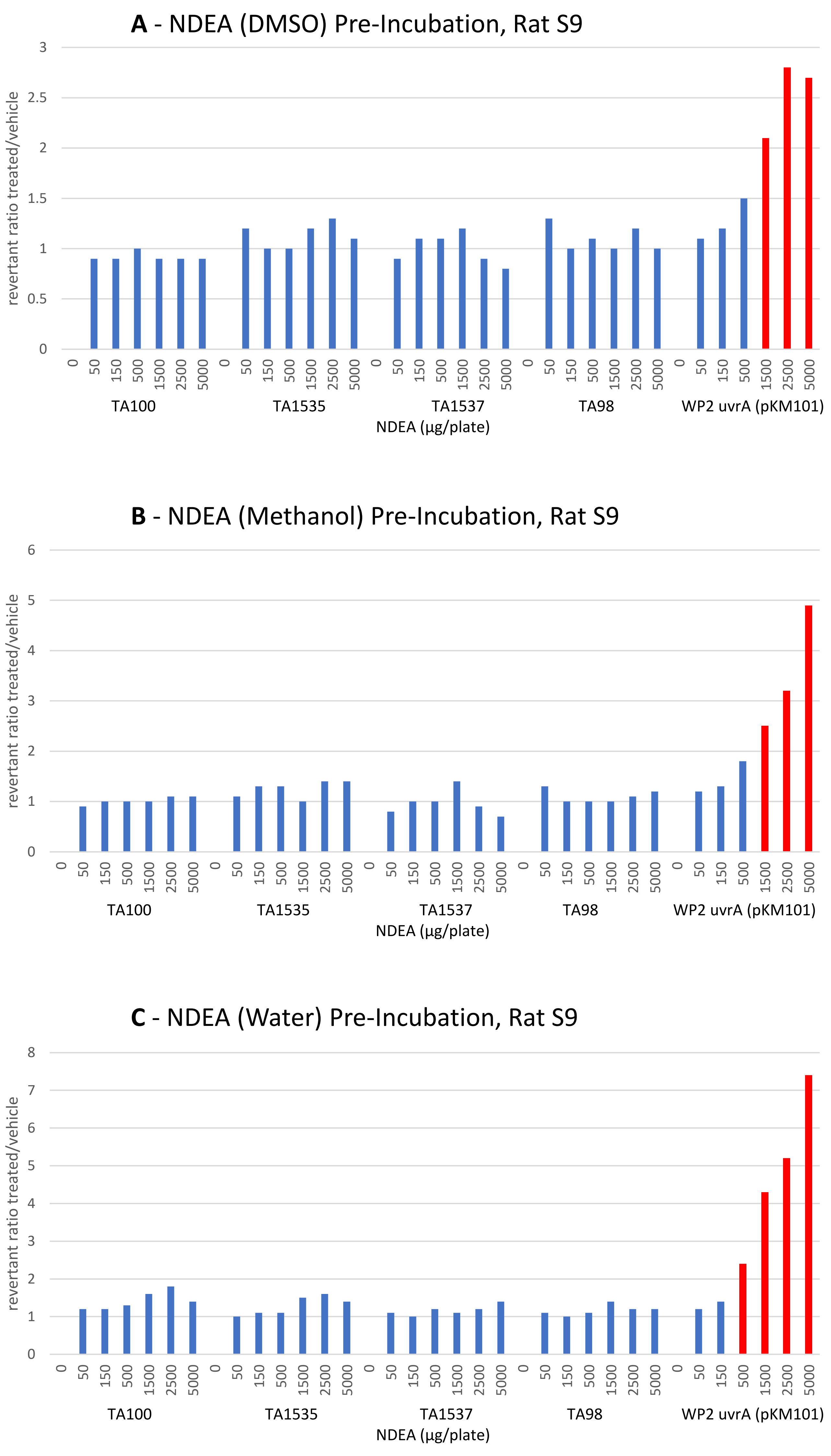
A - NDEA (DMSO) Pre-Incubation, Rat S9
revertant ratio treated/vehicle
0
0.5
1
1.5
2
2.5
3
0 50 150 500 1500 2500 5000
TA100
TA1535
TA1537
TA98
WP2 uvrA (pKM101)
NDEA (µg/plate)
B - NDEA (Methanol) Pre-Incubation, Rat S9
revertant ratio treated/vehicle
0
1
2
3
4
5
6
0 50 150 500 1500 2500 5000
TA100
TA1535
TA1537
TA98
WP2 uvrA (pKM101)
NDEA (µg/plate)
C - NDEA (Water) Pre-Incubation, Rat S9
revertant ratio treated/vehicle
0
1
2
3
4
5
6
7
8
0 50 150 500 1500 2500 5000
TA100
TA1535
TA1537
TA98
WP2 uvrA (pKM101)
NDEA (µg/plate)

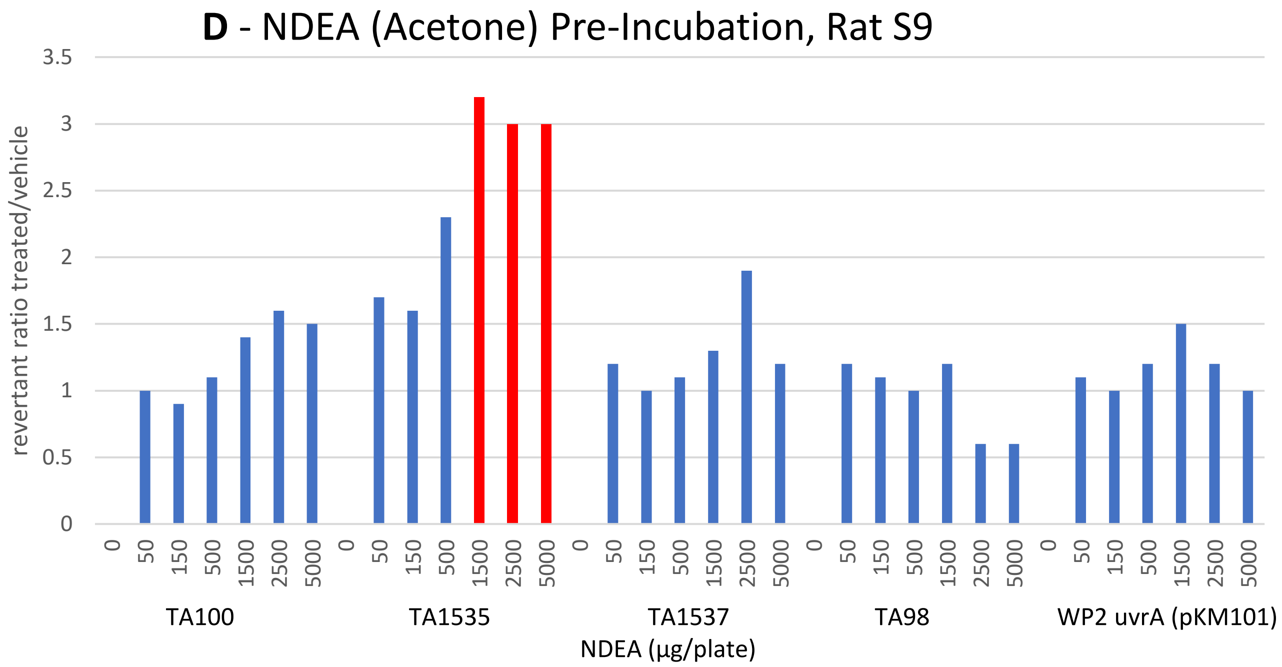
D - NDEA (Acetone) Pre-Incubation, Rat S9
revertant ratio treated/vehicle
3.5
3
2.5
2
1.5
1
0.5
0
0 50 150 500 1500 2500 5000 0 50 150 500 1500 2500 5000 0 50 150 500 1500 2500 5000 0 50 150 500 1500 2500 5000 0 50 150 500 1500 2500 5000
TA100
TA1535
TA1537
TA98
WP2 uvrA (pKM101)
NDEA (µg/plate)

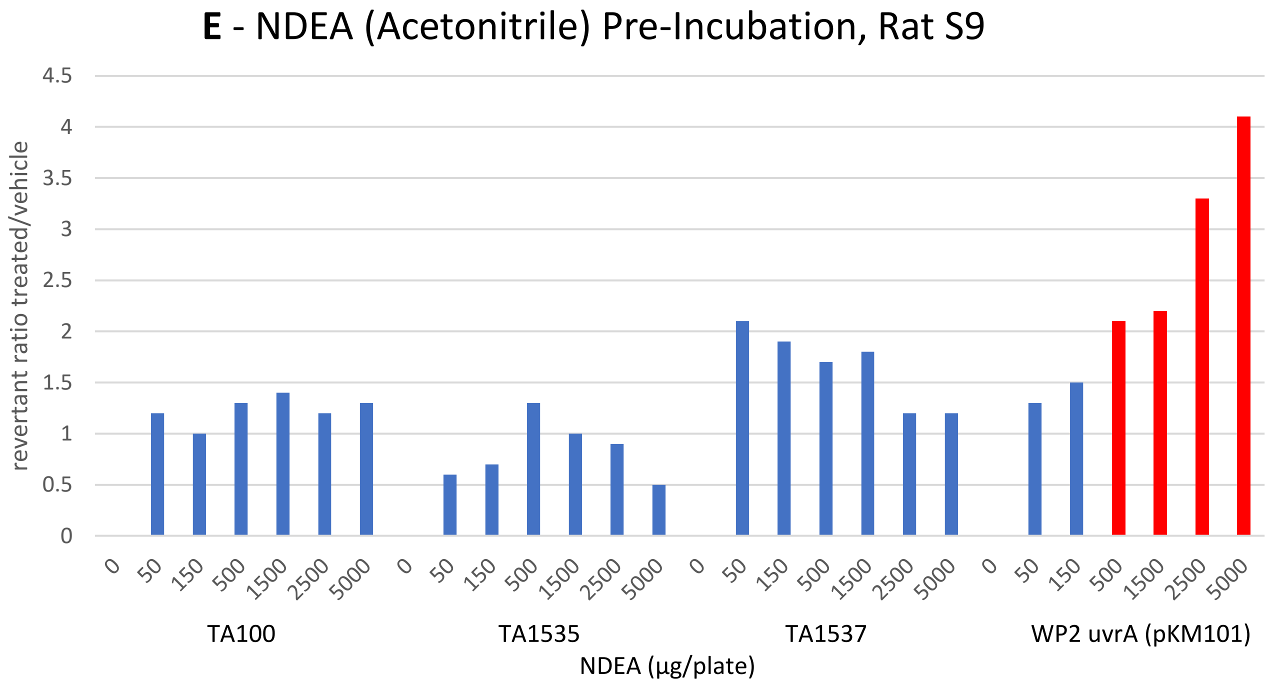
E - NDEA (Acetonitrile) Pre-Incubation, Rat S9
revertant ratio treated/vehicle
4.5
4
3.5
3
2.5
2
1.5
1
0.5
0
0 50 150 500 1500 2500 5000 0 50 150 500 1500 2500 5000 0 50 150 500 1500 2500 5000 0 50 150 500 1500 2500 5000
TA100
TA1535
TA1537
WP2 uvrA (pKM101)
NDEA (µg/plate)

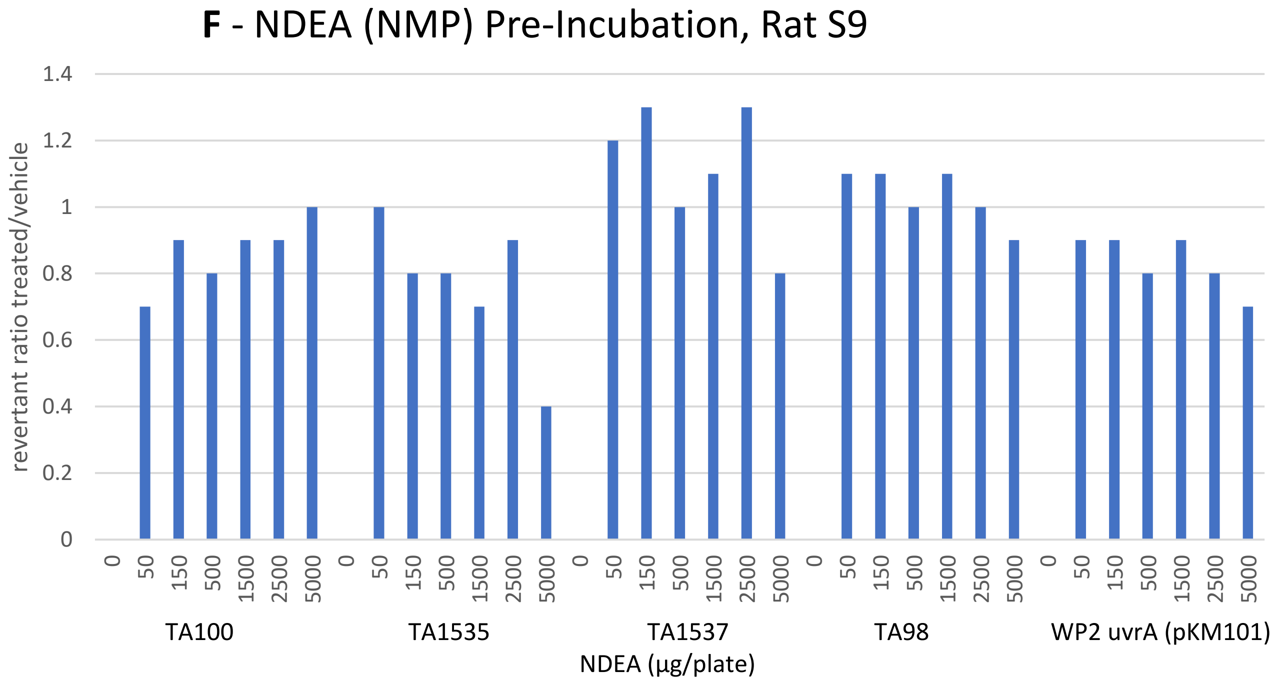
F - NDEA (NMP) Pre-Incubation, Rat S9
revertant ratio treated/vehicle
1.4
1.2
1
0.8
0.6
0.4
0.2
0
0 50 150 500 1500 2500 5000 0 50 150 500 1500 2500 5000 0 50 150 500 1500 2500 5000 0 50 150 500 1500 2500 5000 0 50 150 500 1500 2500 5000
TA100
TA1535
TA1537
TA98
WP2 uvrA (pKM101)
NDEA (µg/plate)

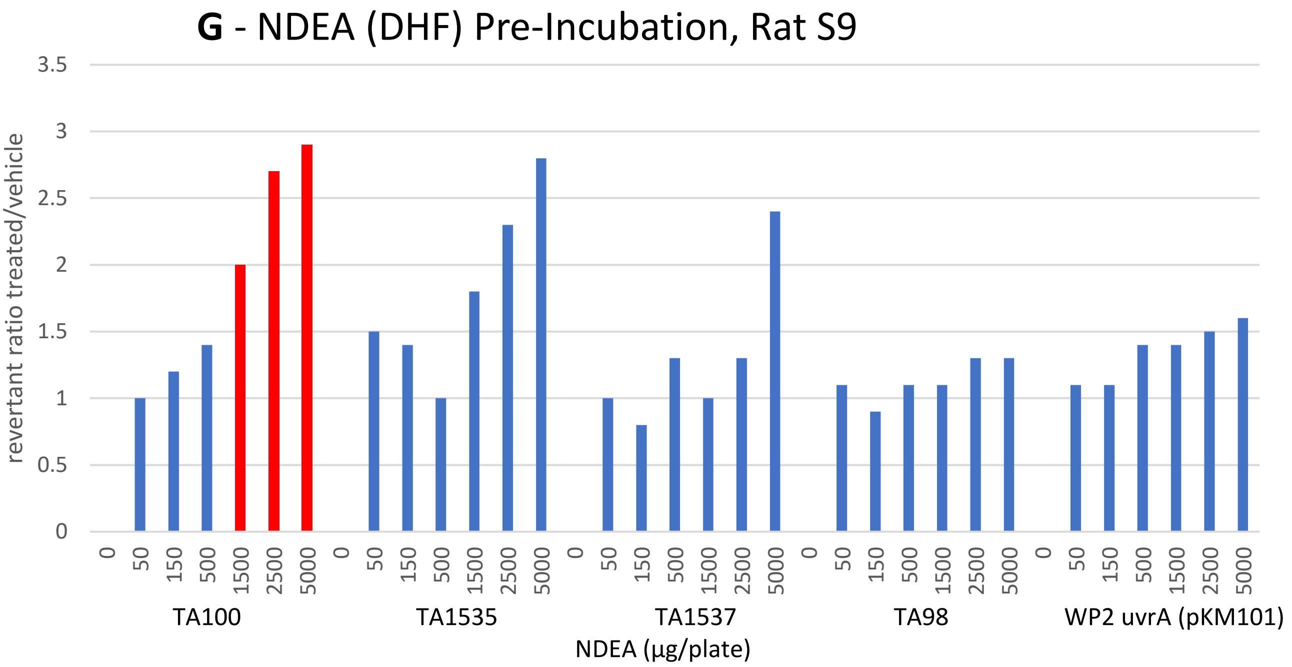

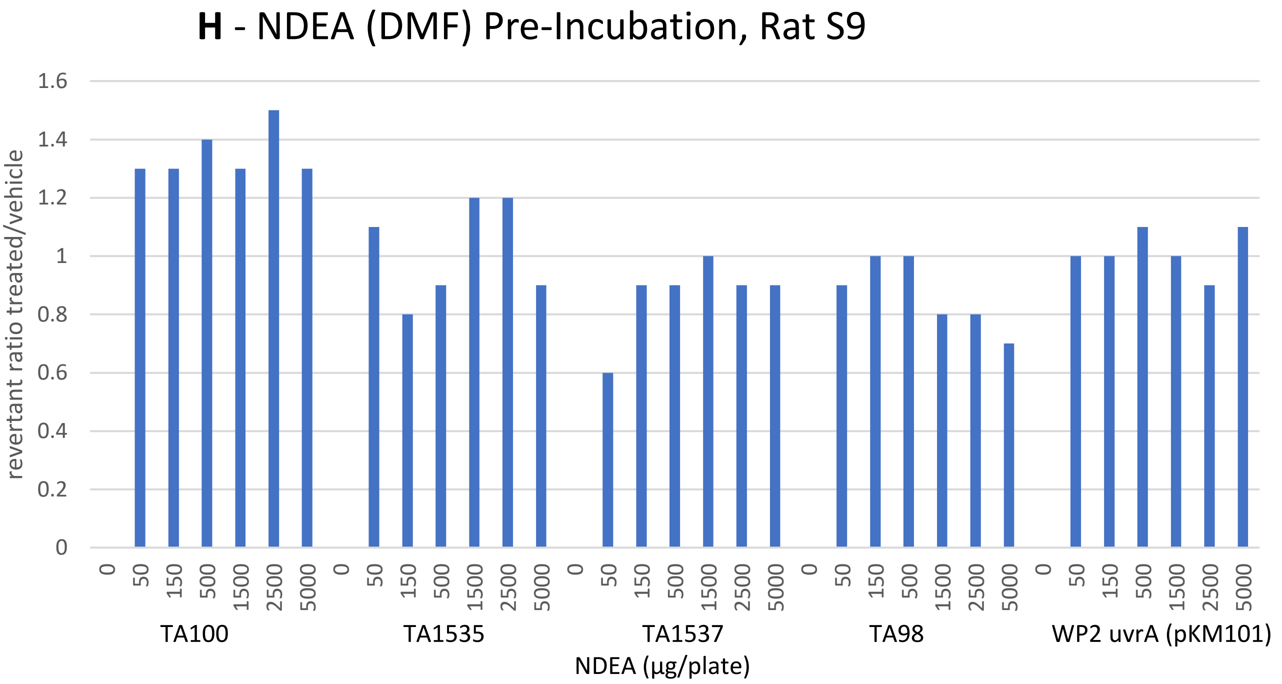

Figure 8 (Suppl.) Bacterial reverse mutation pre-incubation mean revertant ratio treated/vehicle control data with NDEA, using solvent vehicles DMSO (**A**), Methanol (**B**), Water (**C**), Acetone (**D**), Acetonitrile (**E**), NMP (**F**), DHF (**G**) and DMF (**H**), in the presence of Rat liver S9-mix (Y axis representative of mean revertant ratio treated/vehicle, X axis representative of test article concentration (µg/plate) per bacterial strain). Bars represent concentrations where mean revertant ratio treated/vehicle is less than (blue) or exceed (red) the 2-fold for TA100, TA98 and WP2uvrA (pKM101) and 3-fold for TA1535 and TA1537. The maximum concentration tested was 5000 ug/plate, the maximum concentration in accordance with current guidelines. N.b Due to toxicity, no data available for TA98 using acetonitrile as a vehicle.

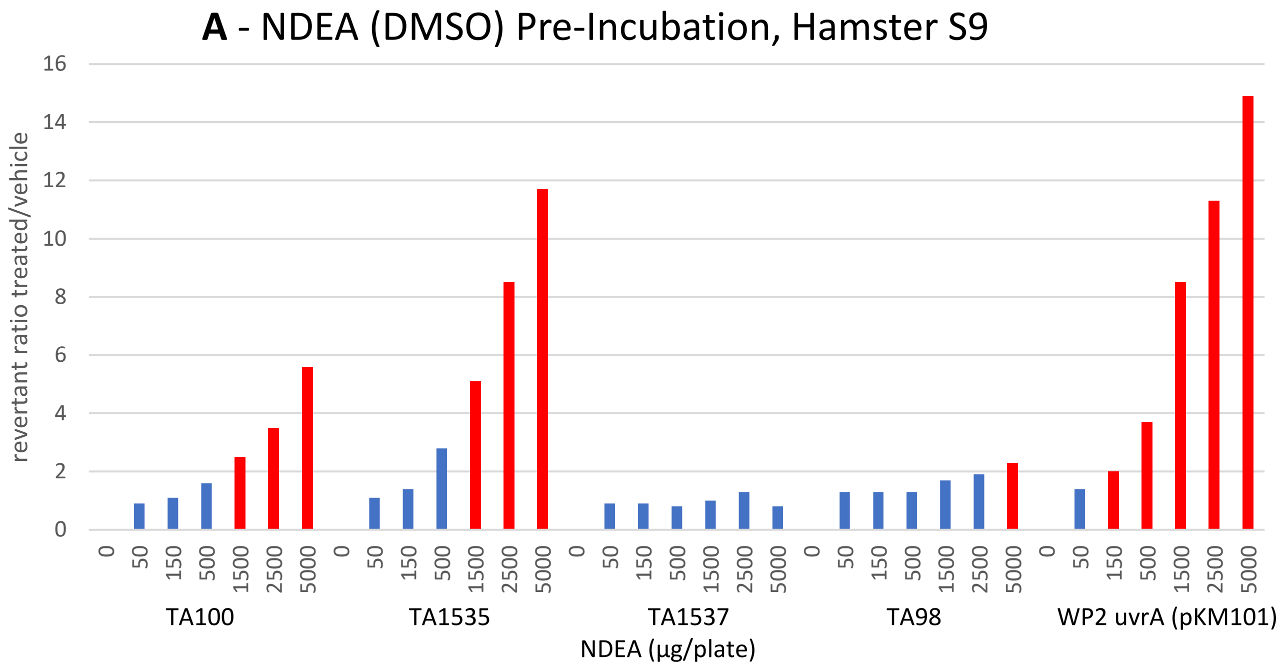
A - NDEA (DMSO) Pre-Incubation, Hamster S9
revertant ratio treated/vehicle
16
14
12
10
8
6
4
2
0
0 50 150 500 1500 2500 5000
TA100
TA1535
TA1537
TA98
WP2 uvrA (pKM101)
NDEA (µg/plate)

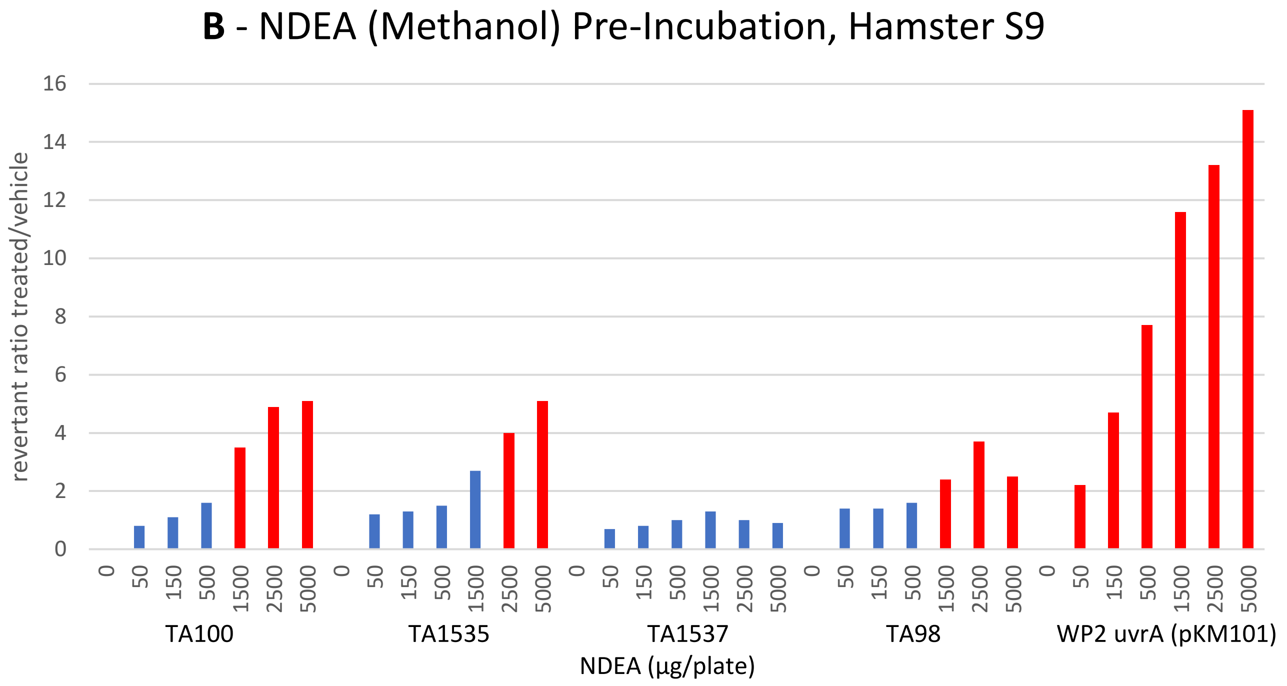
B - NDEA (Methanol) Pre-Incubation, Hamster S9
revertant ratio treated/vehicle
16
14
12
10
8
6
4
2
0
0 50 150 500 1500 2500 5000
TA100
TA1535
TA1537
TA98
WP2 uvrA (pKM101)
NDEA (µg/plate)

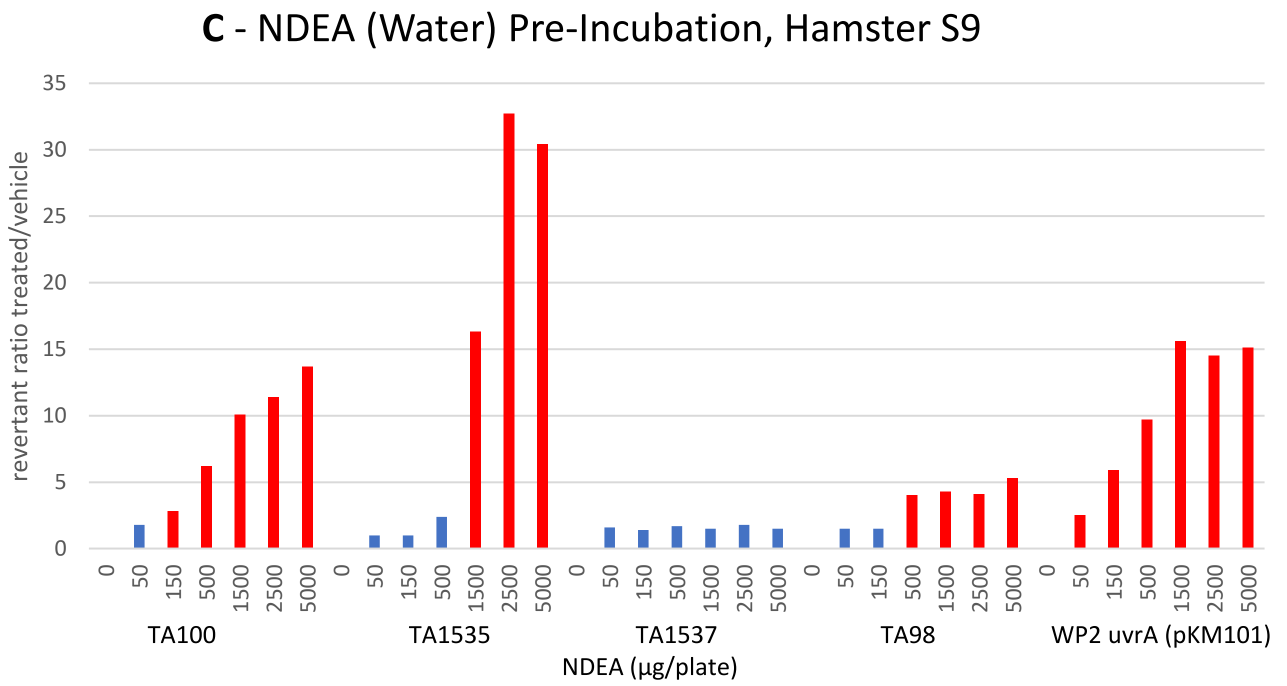
C - NDEA (Water) Pre-Incubation, Hamster S9
revertant ratio treated/vehicle
35
30
25
20
15
10
5
0
0 50 150 500 1500 2500 5000
TA100
TA1535
TA1537
TA98
WP2 uvrA (pKM101)
NDEA (µg/plate)

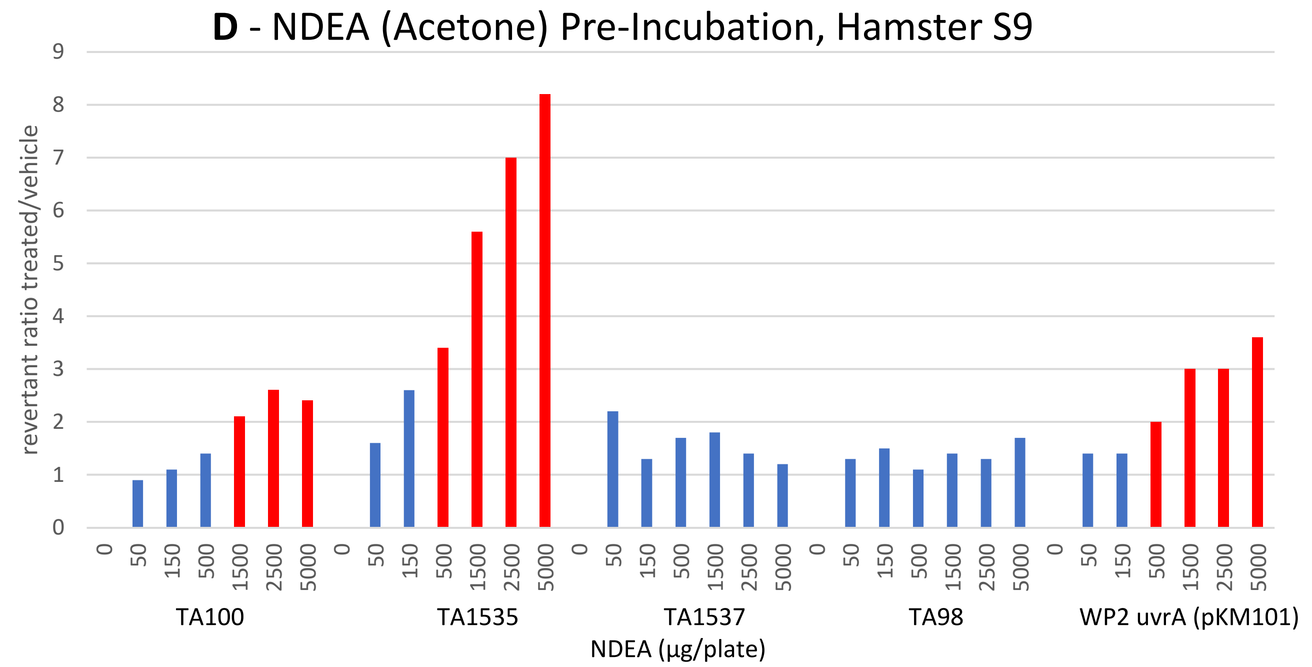
D - NDEA (Acetone) Pre-Incubation, Hamster S9
revertant ratio treated/vehicle
0 1 2 3 4 5 6 7 8 9
0 50 150 500 1500 2500 5000
TA100
TA1535
TA1537
TA98
WP2 uvrA (pKM101)
NDEA (µg/plate)

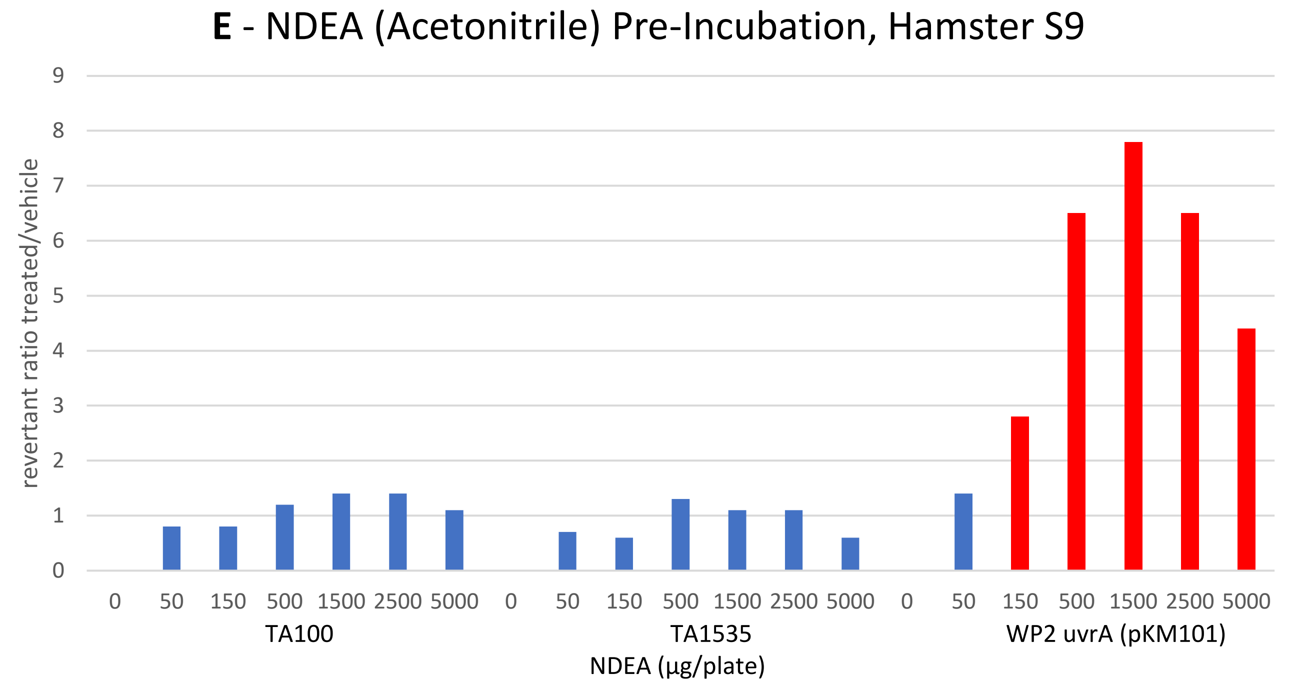
E - NDEA (Acetonitrile) Pre-Incubation, Hamster S9
revertant ratio treated/vehicle
0 1 2 3 4 5 6 7 8 9
0 50 150 500 1500 2500 5000
TA100
TA1535
WP2 uvrA (pKM101)
NDEA (µg/plate)

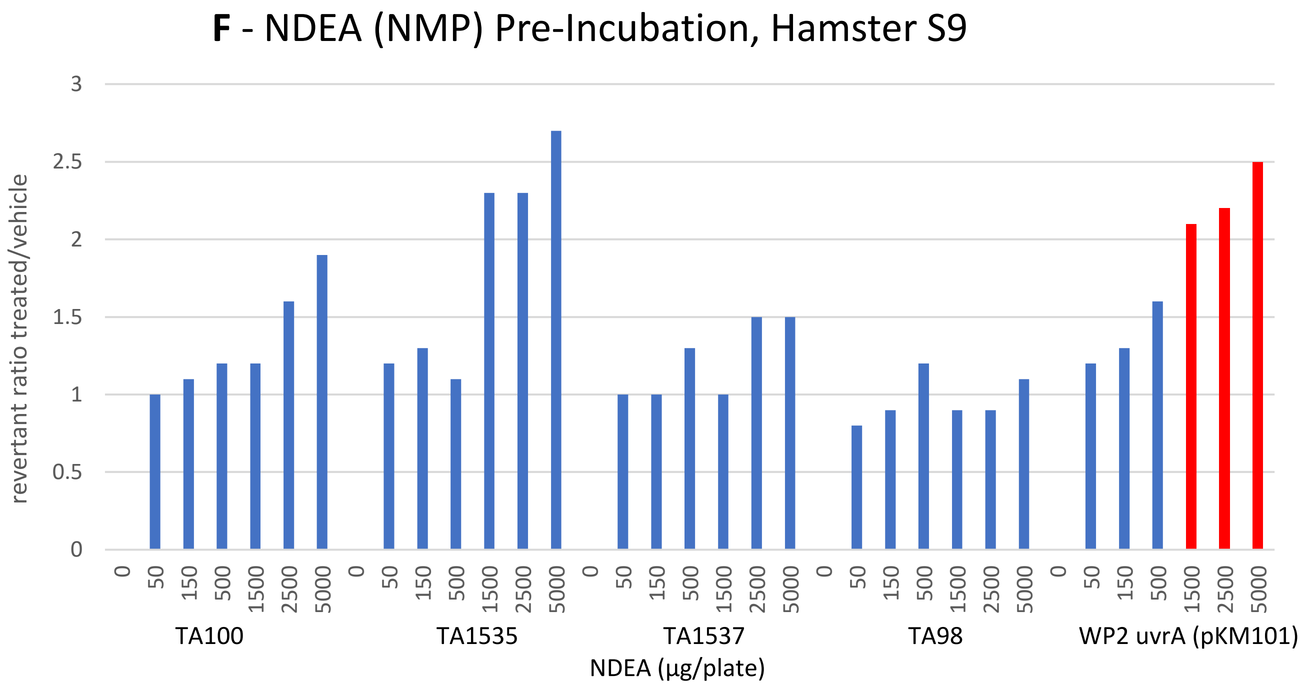
F - NDEA (NMP) Pre-Incubation, Hamster S9
revertant ratio treated/vehicle
0 0.5 1 1.5 2 2.5 3
0 50 150 500 1500 2500 5000
TA100
TA1535
TA1537
TA98
WP2 uvrA (pKM101)
NDEA (µg/plate)

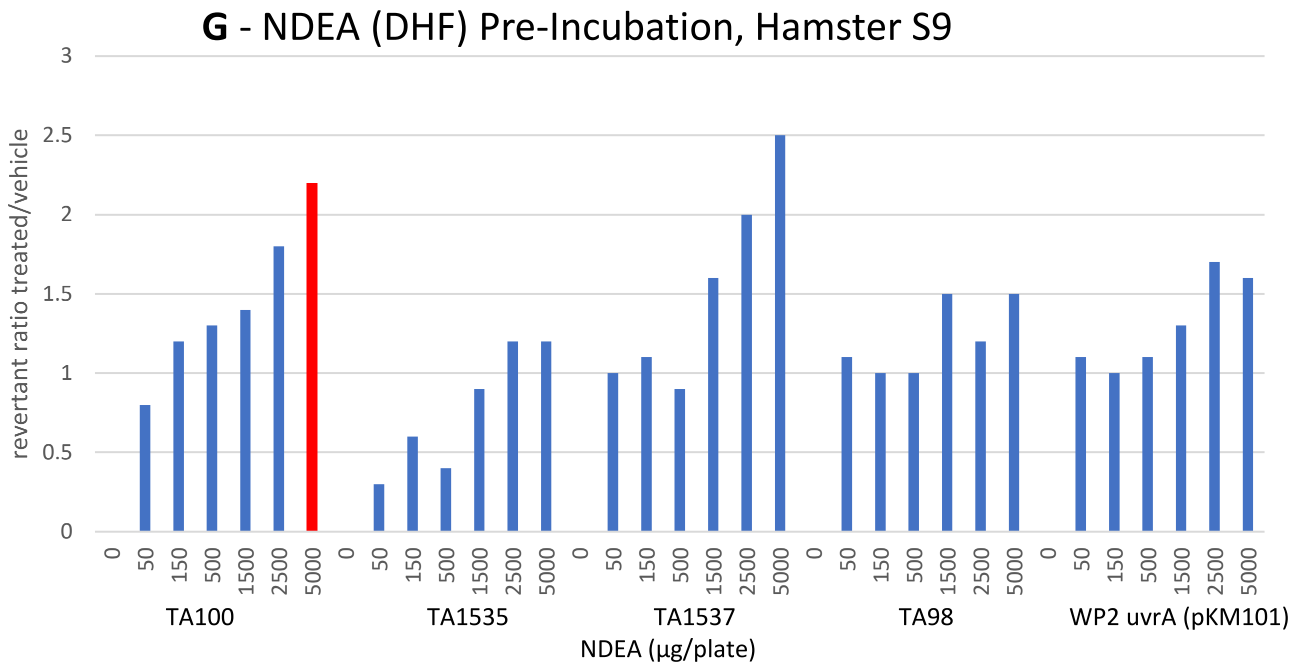

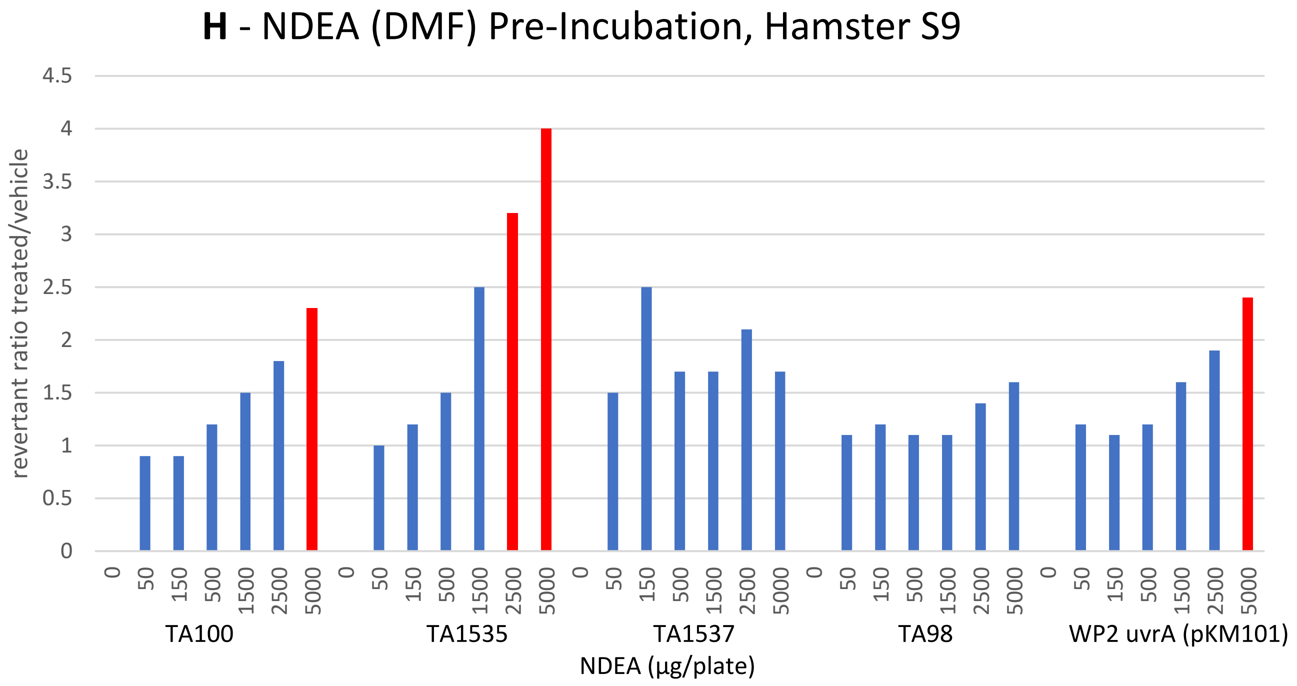

Figure 9 (Suppl.) Bacterial reverse mutation pre-incubation mean revertant ratio treated/vehicle control data with NDEA, using solvent vehicles DMSO (**A**), Methanol (**B**), Water (**C**), Acetone (**D**), Acetonitrile (**E**), NMP (**F**), DHF (**G**) and DMF (**H**), in the presence of Hamster liver S9-mix (Y axis representative of mean revertant ratio treated/vehicle, X axis representative of test article concentration (µg/plate) per bacterial strain). Bars represent concentrations where mean revertant ratio treated/vehicle is less than (blue) or exceed (red) the 2-fold for TA100, TA98 and WP2uvrA (pKM101) and 3-fold for TA1535 and TA1537. The maximum concentration tested was 5000 ug/plate, the maximum concentration in accordance with current guidelines. N.b Due to toxicity, no data available for TA98 or TA1537 using acetonitrile as a vehicle.

Ames Test study designs for nitrosamine mutagenicity testing: qualitative and quantitative analysis of key assay parameters – Supplementary Data

NDMA and NDEA Bench-Mark dose analyses.

Authors:

Dean N. Thomas, John W. Wills, Helen Tracey, Sandy J. Baldwin, Mark Burman, Abbie N. Williams, Dannii S.G. Harte, Ruby A. Buckley and Anthony M. Lynch

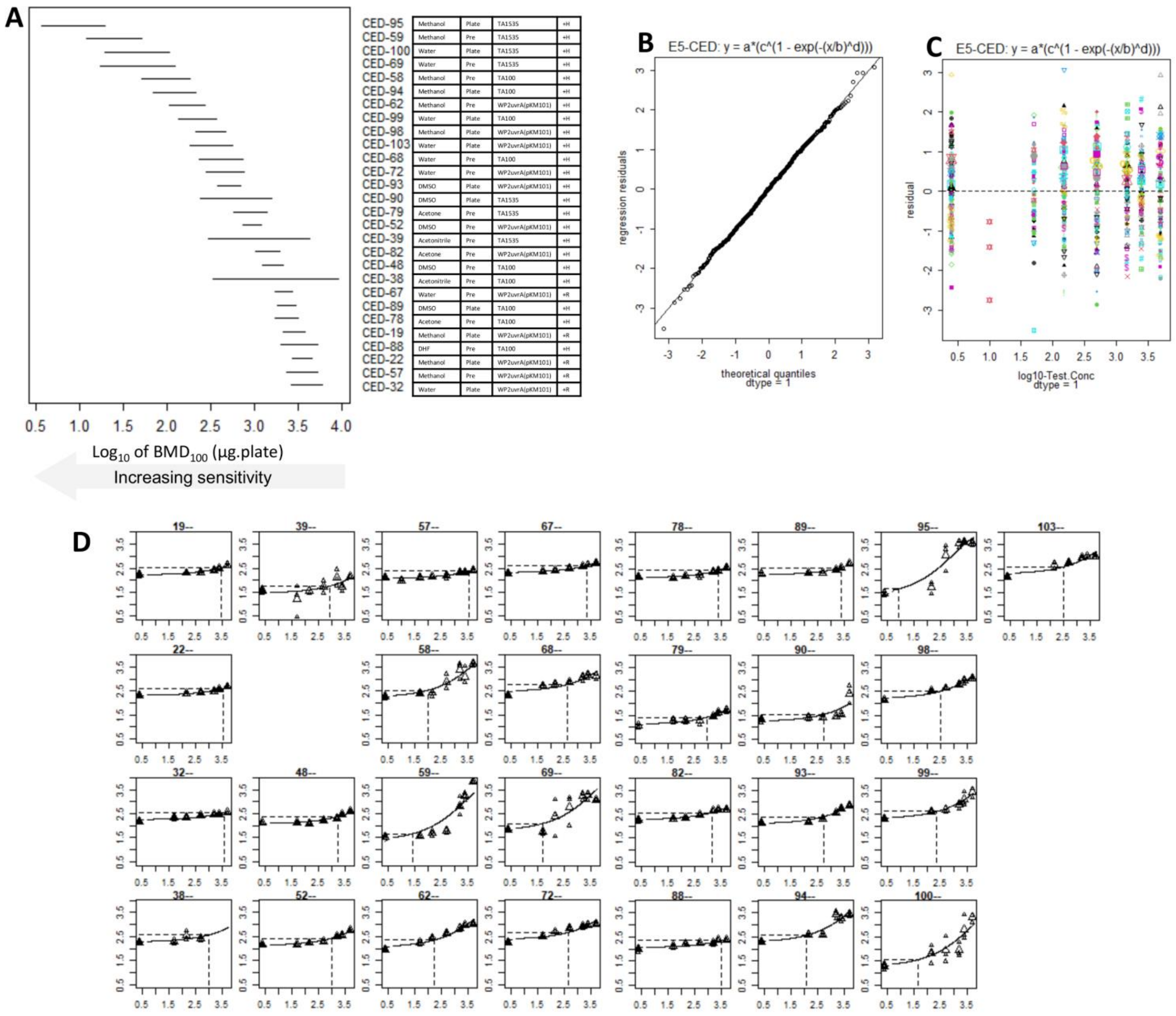

Figure 10 (Suppl.) – BMD-derived sensitivity ranking for positive NDMA studies. A, Sensitivity ranking using 90% confidence intervals of the BMD_{100} (*i.e.,* 90% confidence interval of the dose estimated to cause a two-fold increase in response relative to vehicle control.). For each confidence interval, test conditions (*i.e.,* vehicle, incubation method, strain and S9 source) are indicated in the Table on the right-side of the plot. **B/C**, quantile-quantile and residuals against dose (respectively) for the fitted dose-response data showing approximate normality and variance homogeneity on $\log_{10}$ scale. **D**, Exponential model fits to the dose-response data underlying the BMD confidence intervals shown in **A**. Horizontal and vertical dashed lines indicate interpolation at the benchmark response of 100% to define the BMD_{100} (respectively).

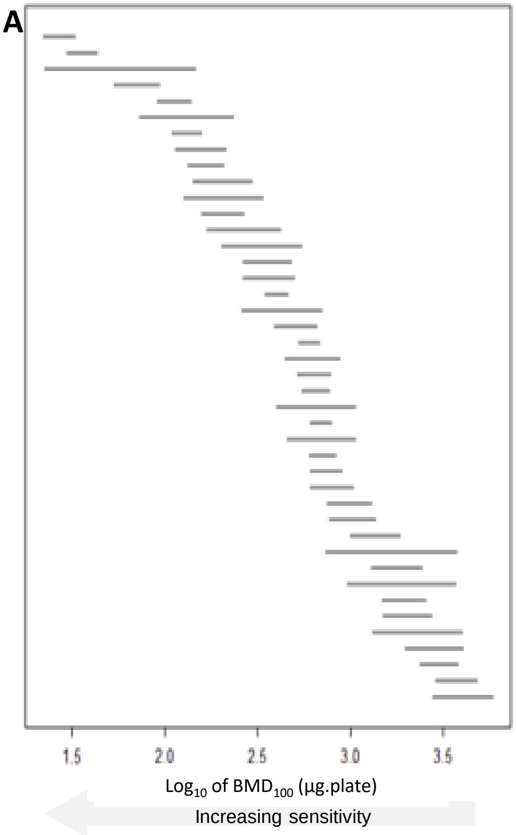

Water	Pre	WP2uvrA(pKM101)	+H
Methanol	Pre	WP2uvrA(pKM101)	+H
Acetonitrile	Pre	WP2uvrA(pKM101)	+H
Water	Pre	TA100	+H
Methanol	Plate	WP2uvrA(pKM101)	+H
Water	Pre	TA1535	+H
DMSO	Pre	WP2uvrA(pKM101)	+H
Water	Plate	WP2uvrA(pKM101)	+H
DMSO	Plate	WP2uvrA(pKM101)	+H
DMSO	Pre	TA1535	+H
Methanol	Plate	TA1535	+H
Water	Plate	TA1535	+H
DMSO	Plate	TA1535	+H
Acetone	Pre	TA1535	+H
Methanol	Plate	TA100	+H
Methanol	Plate	TA98	+H
Water	Pre	WP2uvrA(pKM101)	+R
Acetonitrile	Pre	WP2uvrA(pKM101)	+R
Methanol	Pre	TA100	+H
Water	Plate	TA100	+H
DMSO	Plate	TA98	+H
Methanol	Plate	WP2uvrA(pKM101)	+R
DMSO	Plate	TA100	+H
Water	Pre	TA98	+H
Water	Plate	WP2uvrA(pKM101)	+R
Water	Plate	TA98	+H
DMSO	Pre	TA100	+H
DMSO	Plate	WP2uvrA(pKM101)	+R
Methanol	Pre	TA1535	+H
Methanol	Pre	WP2uvrA(pKM101)	+R
DMF	Pre	TA1535	+H
Acetone	Pre	WP2uvrA(pKM101)	+H
NMP	Pre	TA1535	+H
DHF	Pre	TA100	+R
Acetone	Pre	TA1535	+R
DMSO	Pre	WP2uvrA(pKM101)	+R
Acetone	Pre	TA100	+H
Methanol	Pre	TA98	+H
NMP	Pre	WP2uvrA(pKM101)	+H
DMF	Pre	TA100	+H
DMF	Pre	WP2uvrA(pKM101)	+H
DMSO	Pre	TA98	+H

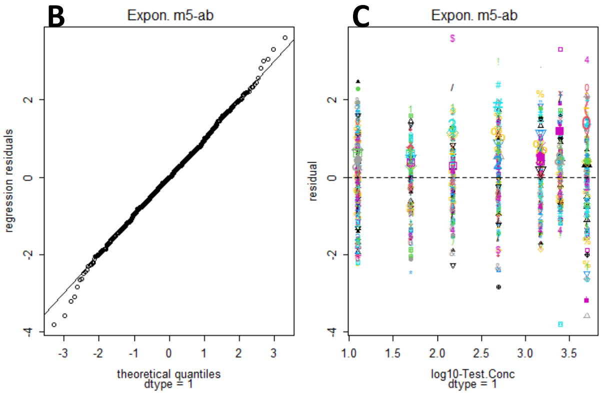

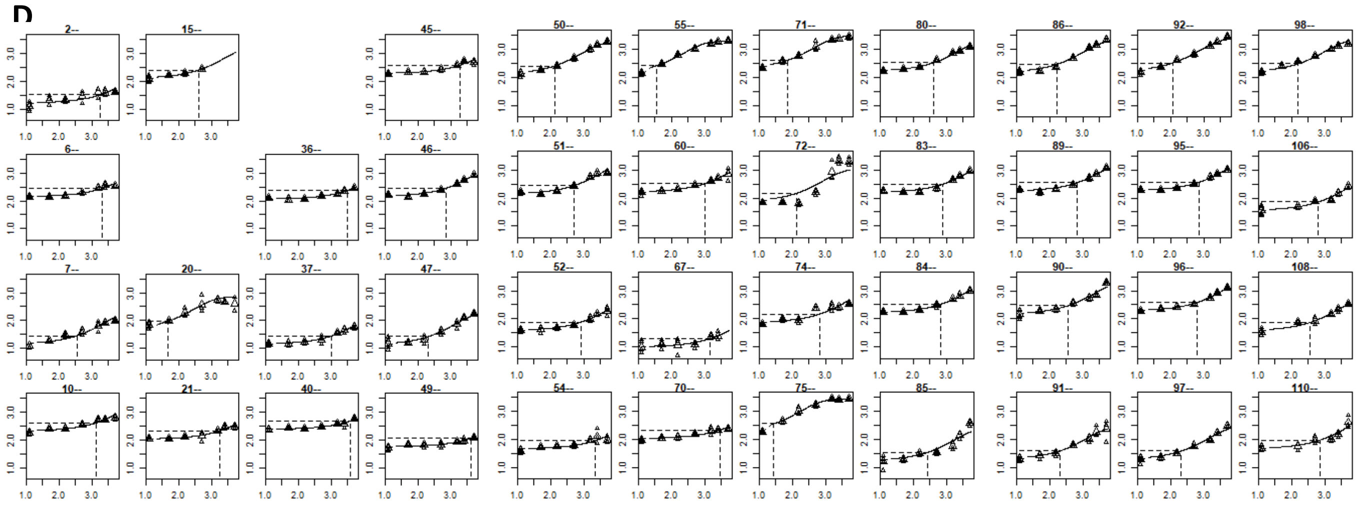

Figure 11 (Suppl.) – BMD-derived sensitivity ranking for positive NDEA studies. **A**, Sensitivity ranking using 90% confidence intervals of the BMD_{100} (*i.e.,* 90% confidence interval of the dose estimated to cause a two-fold increase in response relative to vehicle control.). For each confidence interval, test conditions (*i.e.,* vehicle, incubation method, strain and S9 source) are indicated in the Table on the right-side of the plot. **B/C**, quantile-quantile and residuals against dose (respectively) for the fitted dose-response data showing approximate normality and variance homogeneity on log_{10} scale. **D**, Exponential model fits to the dose-response data underlying the BMD confidence intervals shown in **A**. Horizontal and vertical dashed lines indicate interpolation at the benchmark response of 100% to define the BMD_{100} (respectively).

Qualitative and quantitative assessment of Ames Test assay parameters to

Ames Test study designs for nitrosamine mutagenicity testing: qualitative and quantitative analysis of key assay parameters –

Supplementary Data

NMEA plate incorporation and Pre-Incubation mean ratio treated/vehicle control data Bench-Mark dose analyses.

Authors:

Dean N. Thomas, John W. Wills, Helen Tracey, Sandy J. Baldwin, Mark Burman, Abbie N. Williams, Dannii S.G. Harte, Ruby A. Buckley and Anthony M. Lynch

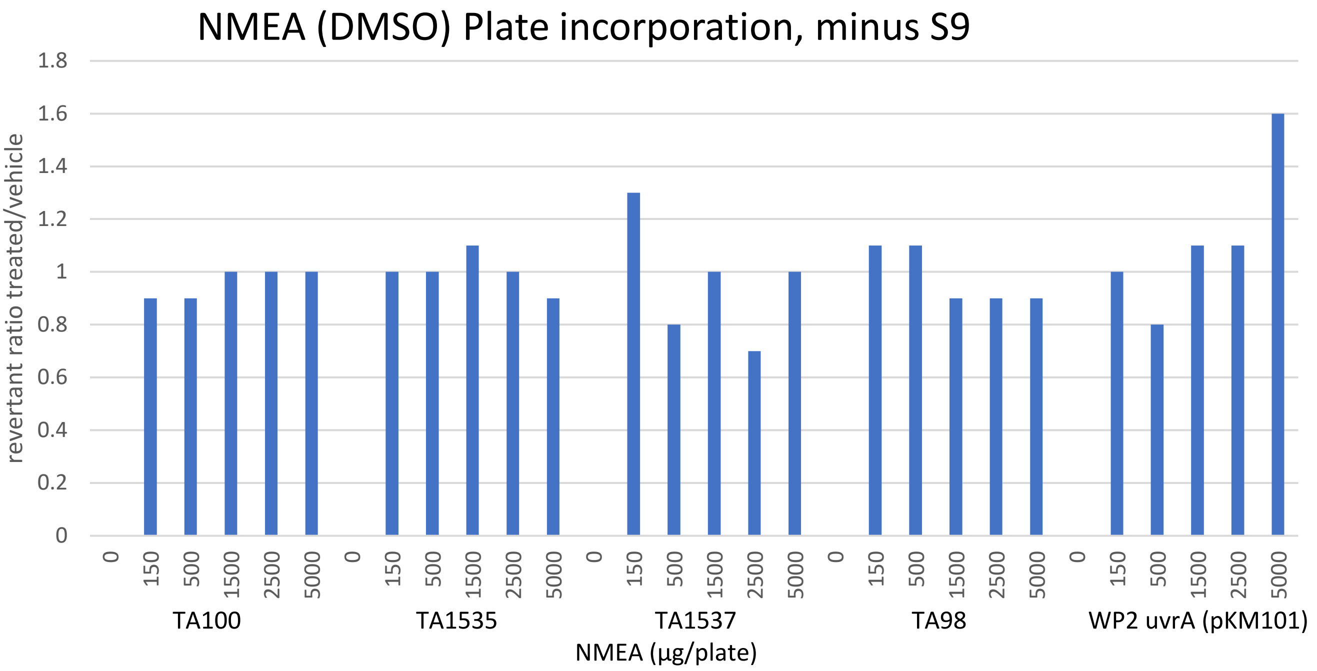

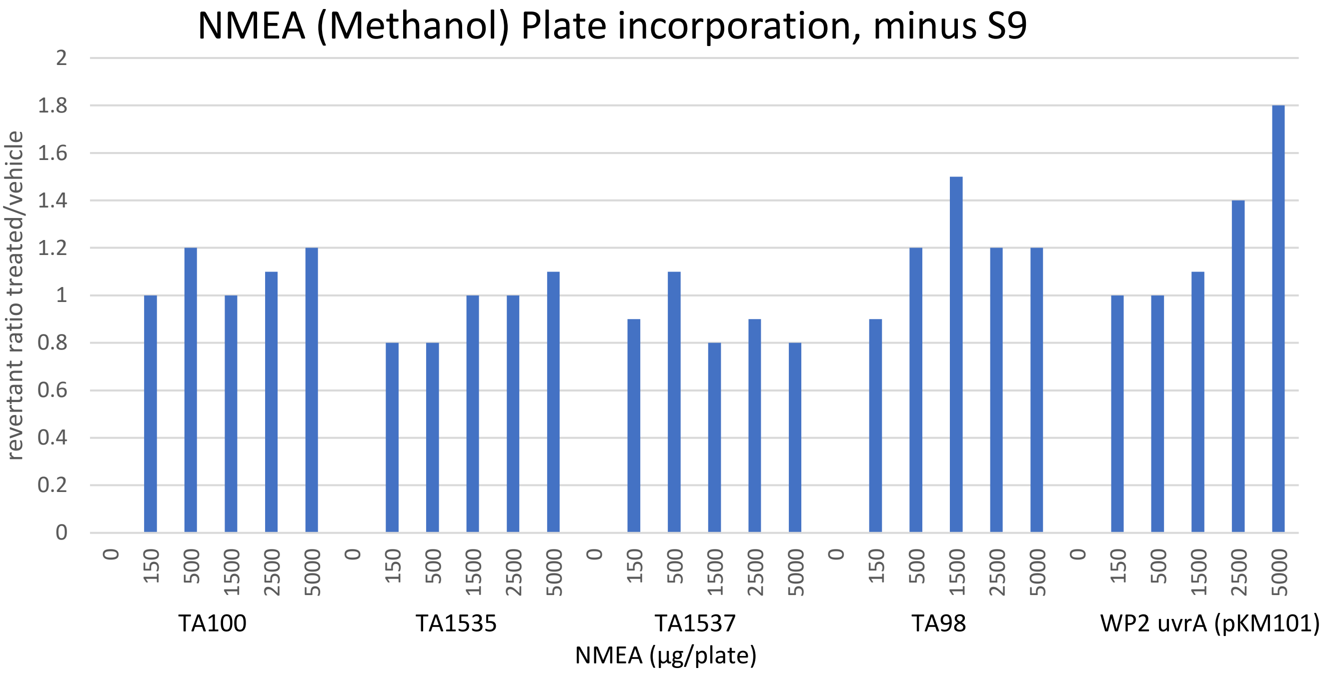

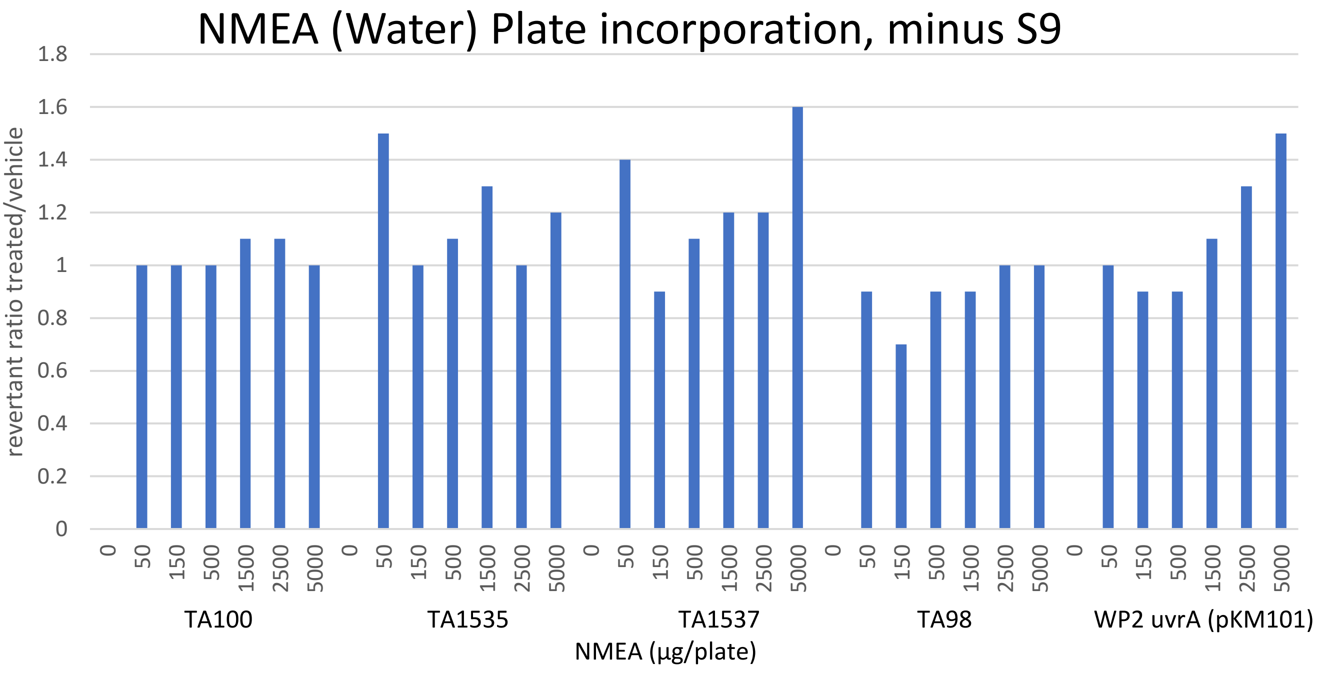

Figure 12 (Suppl.) Bacterial reverse mutation plate incorporation mean revertant ratio treated/vehicle control data with NMEA, using solvent vehicles DMSO (top), methanol (middle) and water (bottom), in the absence of S9-mix (Y axis representative of mean revertant ratio treated/vehicle, X axis representative of test article concentration (µg/plate) per bacterial strain). Blue bars refer to concentrations where mean revertant ratio treated/vehicle is lower than 2-fold for TA100, TA98 and WP2uvrA (pKM101) and 3-fold for TA1535 and TA1537. The maximum concentration tested was 5000 ug/plate, the maximum concentration in accordance with current guidelines.

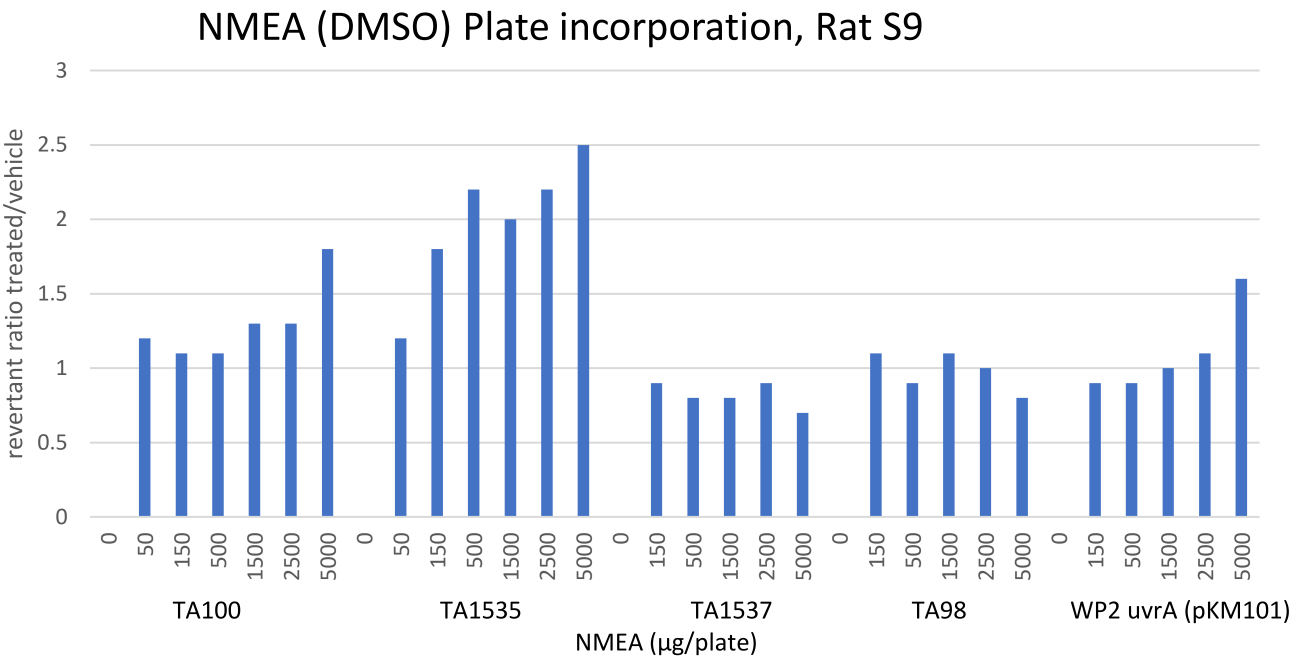

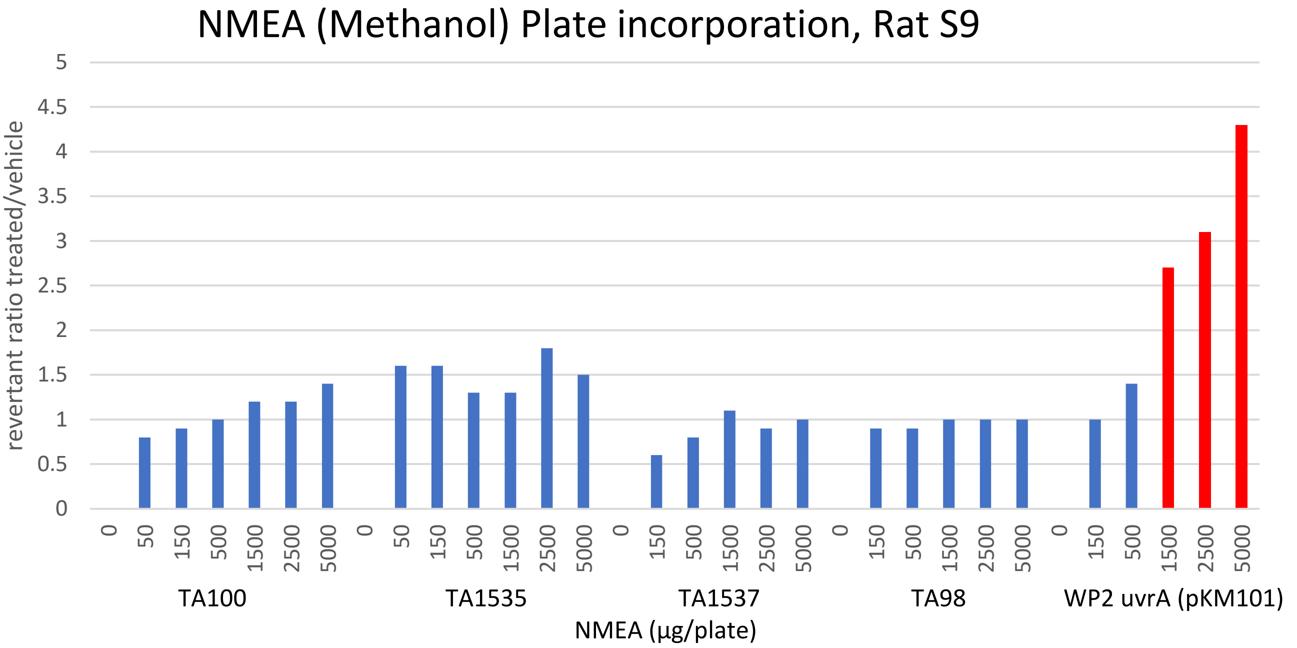

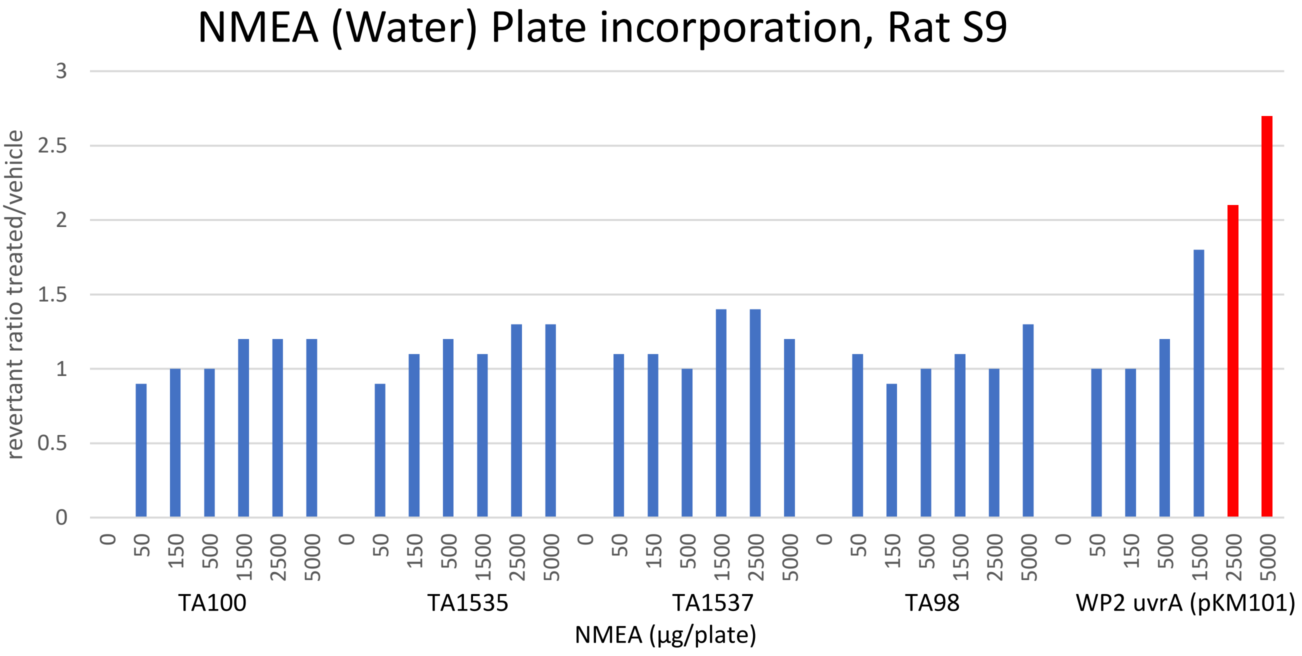

Figure 13 (Suppl.) Bacterial reverse mutation plate incorporation mean revertant ratio treated/vehicle control data with NMEA, using solvent vehicles DMSO (top), methanol (middle) and water (bottom), in the presence of Rat liver S9-mix (Y axis representative of mean revertant ratio treated/vehicle, X axis representative of test article concentration (µg/plate) per bacterial strain). Bars represent concentrations where mean revertant ratio treated/vehicle is less than (blue) or exceed (red) the 2-fold for TA100, TA98 and WP2uvrA (pKM101) and 3-fold for TA1535 and TA1537. The maximum concentration tested was 5000 ug/plate, the maximum concentration in accordance with current guidelines.

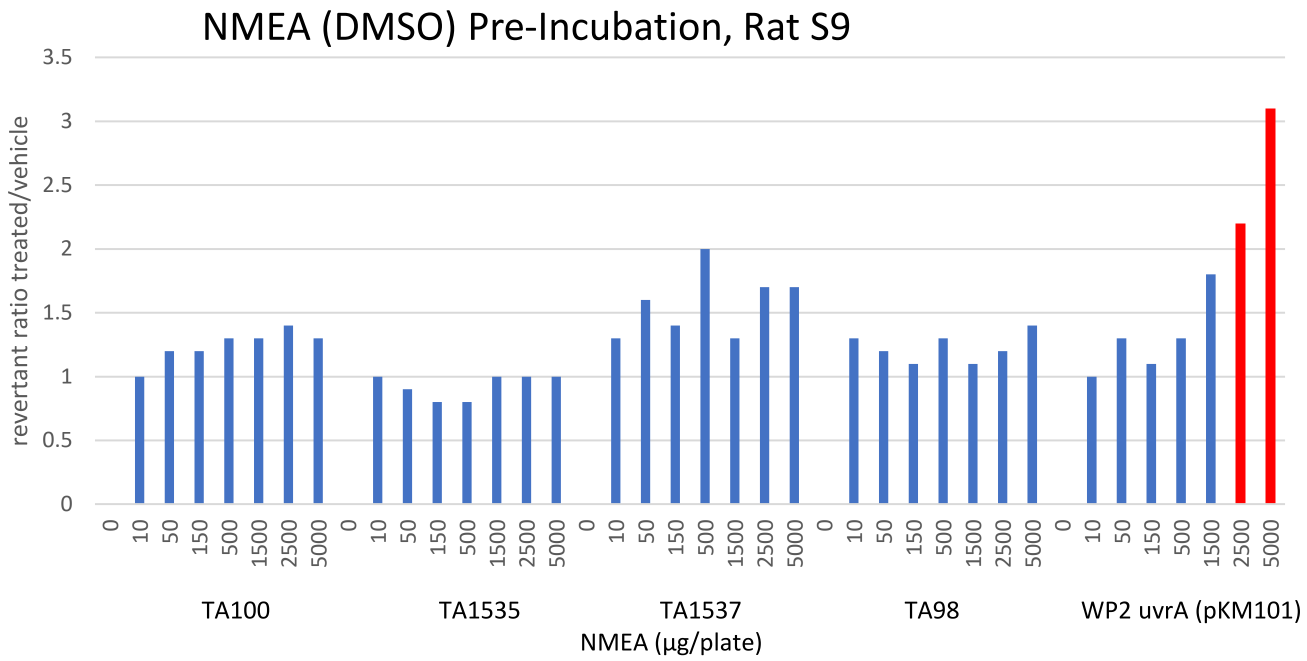

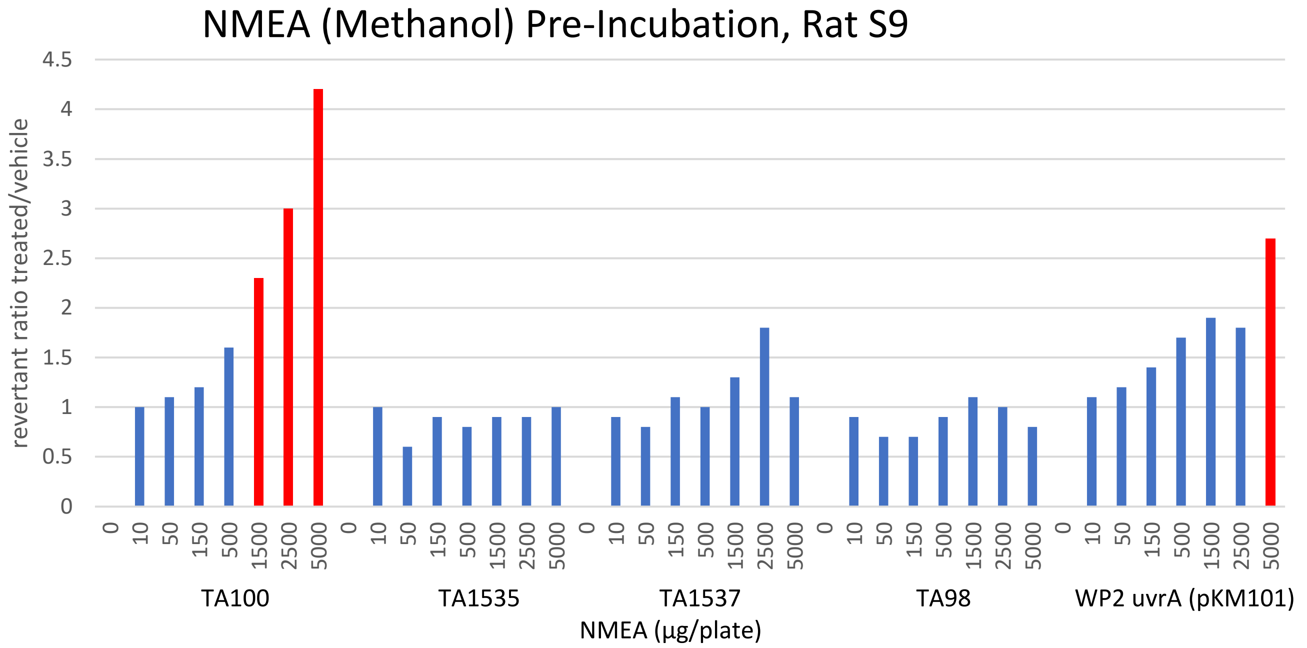

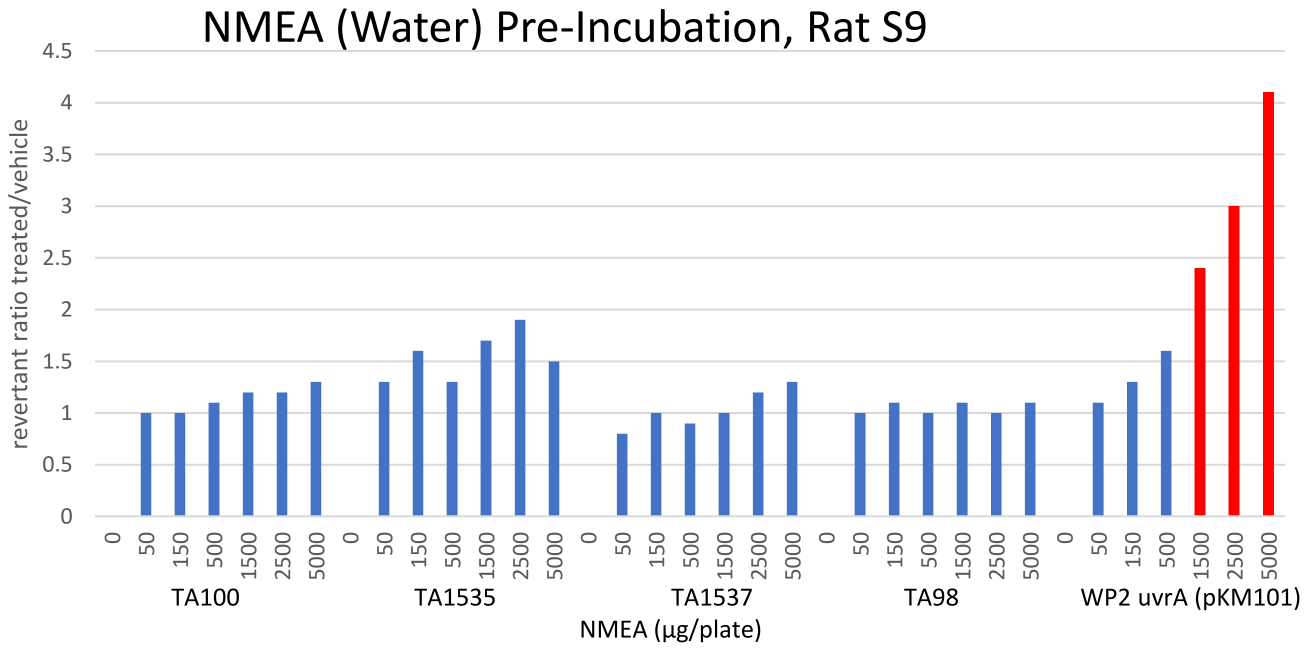

Figure 14 (Suppl.) Bacterial reverse mutation pre-incubation mean revertant ratio treated/vehicle control data with NMEA, using solvent vehicles DMSO (top), methanol (middle) and purified water (bottom), in the presence of Rat liver S9-mix (Y axis representative of mean revertant ratio treated/vehicle, X axis representative of test article concentration (µg/plate) per bacterial strain). Bars represent concentrations where mean revertant ratio treated/vehicle is less than (blue) or exceed (red) the 2-fold for TA100, TA98 and WP2uvrA (pKM101) and 3-fold for TA1535 and TA1537. The maximum concentration tested was 5000 ug/plate, the maximum concentration in accordance with current guidelines

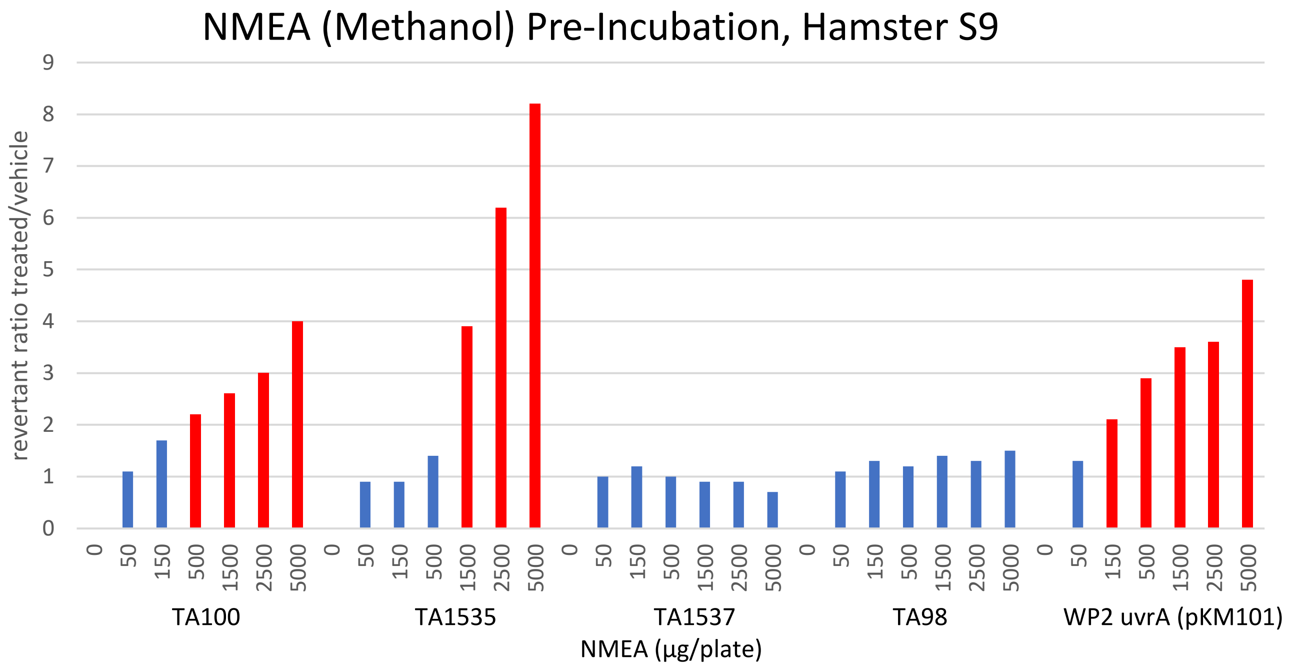

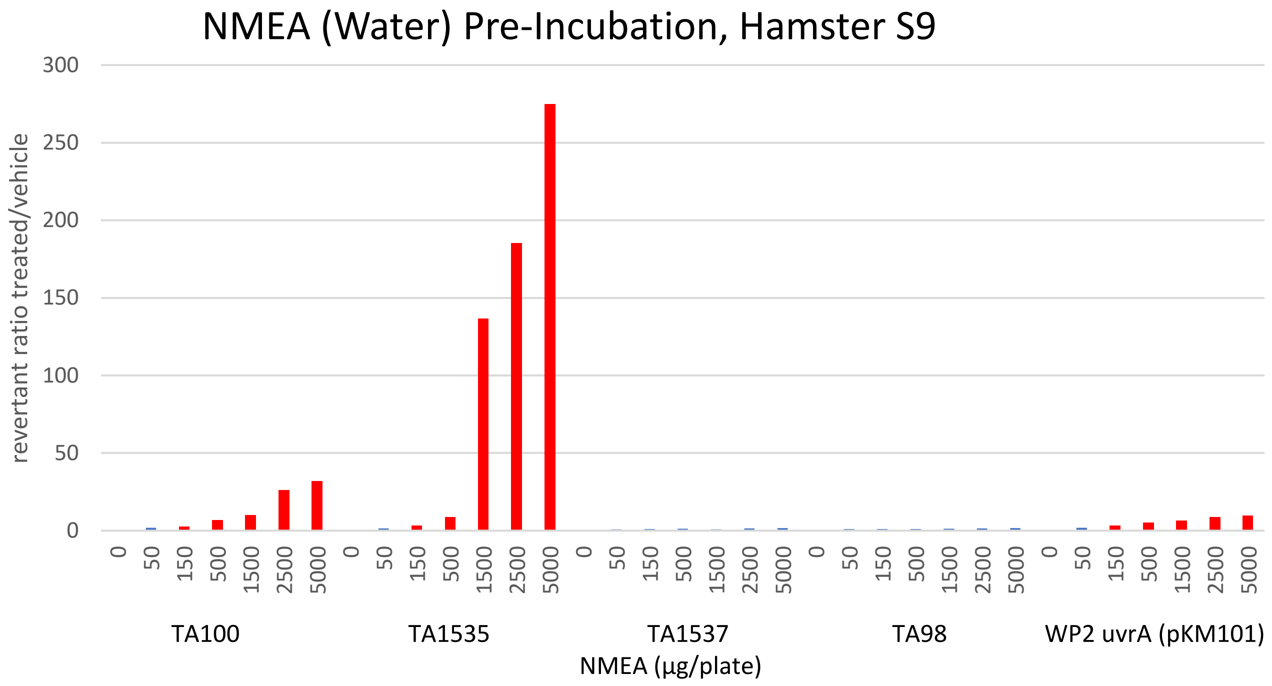

Figure 15 (Suppl.) Bacterial reverse mutation pre-incubation mean revertant ratio treated/vehicle control data with NMEA, using solvent vehicles methanol (top) and purified water (bottom), in the presence of Hamster liver S9-mix (Y axis representative of mean revertant ratio treated/vehicle, X axis representative of test article concentration (µg/plate) per bacterial strain). Bars represent concentrations where mean revertant ratio treated/vehicle is less than (blue) or exceed (red) the 2-fold for TA100, TA98 and WP2uvrA (pKM101) and 3-fold for TA1535 and TA1537. The maximum concentration tested was 5000 ug/plate, the maximum concentration in accordance with current guidelines.

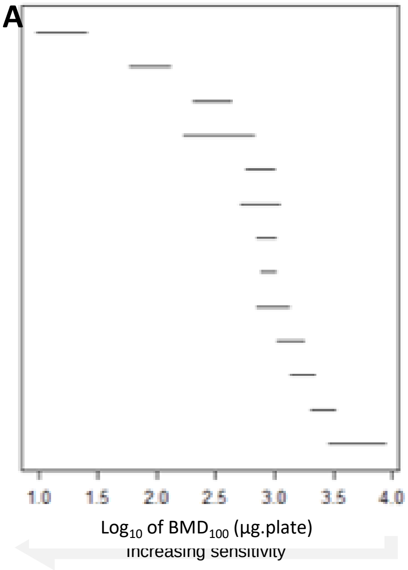

Water	Pre	TA1535	+H
Water	Pre	TA100	+H
Water	Pre	WP2uvrA(pKM101)	+H
Methanol	Pre	TA1535	+H
*Methanol	Plate	WP2uvrA(pKM101)	+R
Methanol	Pre	WP2uvrA(pKM101)	+H
Methanol	Pre	TA100	+R
Water	Pre	WP2uvrA(pKM101)	+R
Methanol	Pre	TA100	+H
Water	Plate	WP2uvrA(pKM101)	+R
DMSO	Pre	WP2uvrA(pKM101)	+R
Methanol	Pre	WP2uvrA(pKM101)	+R
Methanol	Plate	WP2uvrA(pKM101)	+R

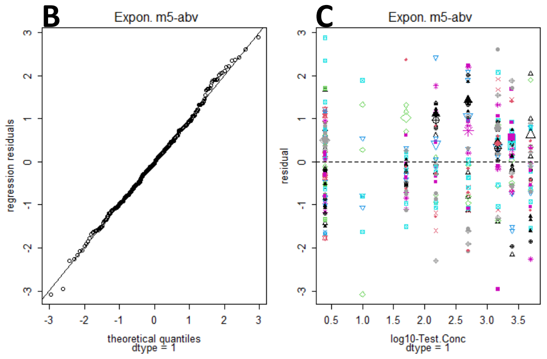

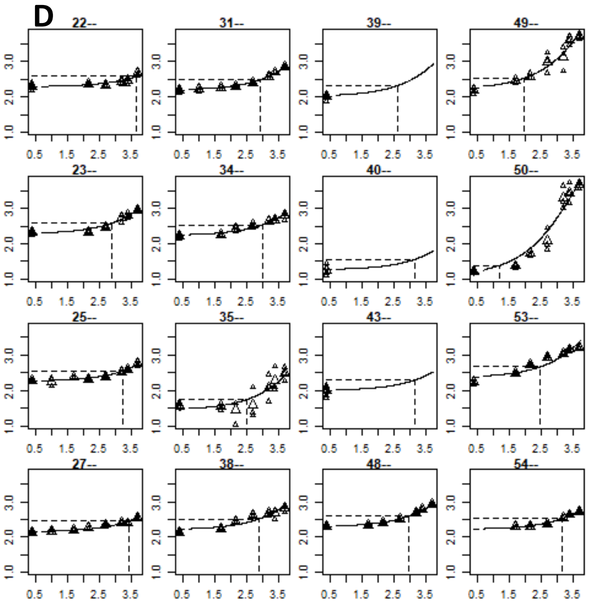

Figure 16 (Suppl.) – BMD-derived sensitivity ranking for positive NMEA studies. A, Sensitivity ranking using 90% confidence intervals of the BMD_{100} (*i.e.,* 90% confidence interval of the dose estimated to cause a two-fold increase in response relative to vehicle control.). For each confidence interval, test conditions (*i.e.,* vehicle, incubation method, strain and S9 source) are indicated in the Table on the right-side of the plot. **B/C**, quantile-quantile and residuals against dose (respectively) for the fitted dose-response data showing approximate normality and variance homogeneity on log_{10} scale. **D**, Exponential model fits to the dose-response data underlying the BMD confidence intervals shown in **A**. Horizontal and vertical dashed lines indicate interpolation at the benchmark response of 100% to define the BMD_{100} (respectively).
